# The effect of exposure to radiofrequency fields on cancer risk in the general and working population: A protocol for a systematic review of human observational studies

**DOI:** 10.1016/j.envint.2021.106828

**Published:** 2021-12

**Authors:** Susanna Lagorio, Maria Blettner, Dan Baaken, Maria Feychting, Ken Karipidis, Tom Loney, Nicola Orsini, Martin Röösli, Marilia Silva Paulo, Mark Elwood

**Affiliations:** a Department of Oncology and Molecular Medicine, National Institute of Health (Istituto Superiore di Sanità), Rome, Italy; b Institute of Medical Biostatistics, Epidemiology and Informatics (IMBEI), University of Mainz, Germany; c Institute of Environmental Medicine, Karolinska Institutet, Stockholm, Sweden; d Australian Radiation Protection and Nuclear Safety Agency (ARPANSA), Yallambie, VIC, Australia; e College of Medicine, Mohammed Bin Rashid University of Medicine and Health Sciences, Dubai, United Arab Emirates; f Department of Global Public Health, Karolinska Institutet, Stockholm, Sweden; g Swiss Tropical and Public Health Institute, Basel, Switzerland; University of Basel, Basel, Switzerland; h Institute of Public Health, College of Medicine and Health Sciences, United Arab Emirates University, Al Ain, United Arab Emirates; i University of Auckland, New Zealand

**Keywords:** Radiofrequency electromagnetic fields, Microwaves, Mobile phones, Cordless phones, Broadcast transmitters, Base stations, Occupational exposure, Neoplasms, Brain cancer, Glioma, Meningioma, Acoustic neuroma, Pituitary tumours, Salivary gland tumours, Childhood leukaemia, Leukaemia, Epidemiology, Cohort studies, Case-control studies, Systematic review protocol

## Abstract

**Background:**

The World Health Organization (WHO) has an ongoing project to assess potential health effects of exposure to radiofrequency electromagnetic fields (RF-EMF) in the general and working population. Here we present the protocol for a systematic review of the scientific literature on cancer hazards from exposure to RF-EMF in humans, commissioned by the WHO as part of that project.

**Objective:**

To assess the quality and strength of the evidence provided by human observational studies for a causal association between exposure to RF-EMF and risk of neoplastic diseases.

**Eligibility criteria:**

We will include cohort and case-control studies investigating neoplasia risks in relation to three types of exposure to RF-EMF: near-field, head-localized, exposure from wireless phone use (SR-A); far-field, whole body, environmental exposure from fixed-site transmitters (SR-B); near/far-field occupational exposures from use of handheld transceivers or RF-emitting equipment in the workplace (SR-C). While no restriction on tumour type will be applied, we will focus on selected neoplasms of the central nervous system (brain, meninges, pituitary gland, acoustic nerve) and salivary gland tumours (SR-A); brain tumours and leukaemias (SR-B, SR-C).

**Information sources:**

Eligible studies will be identified through Medline, Embase, and EMF-Portal.

**Risk-of-bias assessment:**

We will use a tailored version of the OHAT's tool to evaluate the study's internal validity.

**Data synthesis:**

We will consider separately studies on different tumours, neoplasm-specific risks from different exposure sources, and a given exposure-outcome pair in adults and children. When a quantitative synthesis of findings can be envisaged, the main aims of the *meta*-analysis will be to assess the strength of association and the shape of the exposure–response relationship; to quantify the degree of heterogeneity across studies; and explore the sources of inconsistency (if any). When a *meta*-analysis is judged inappropriate, we will perform a narrative synthesis, complemented by a structured tabulation of results and appropriate visual displays.

**Evidence assessment:**

Confidence in evidence will be assessed in line with the GRADE approach.

**Funding:**

This project is supported by the World Health Organization. Co-financing was provided by the New Zealand Ministry of Health; the Istituto Superiore di Sanità in its capacity as a WHO Collaborating Centre for Radiation and Health; ARPANSA as a WHO Collaborating Centre for Radiation Protection.

**Registration:**

PROSPERO CRD42021236798.

## Highlights

• RF-EMF was classified by IARC as possibly carcinogenic to humans (2B) in May 2011
• A systematic review of all subject-relevant epidemiological studies is now needed.
• A detailed protocol ensures the review's transparency, utility and credibility.
• Original study validity will be evaluated with a customized OHAT risk of bias tool.
• Internal coherence and external plausibility will inform conclusions.

## 1 Introduction

### 1.1 Background

The technological applications of radiofrequency electromagnetic fields (RF-EMF; frequencies 100 kHz to 300 GHz) have been steadily increasing since the 1950s. RF-EMF are used in medicine (e.g. magnetic resonance imaging, diathermy, radiofrequency ablation), industry (e.g. heating and welding), domestic appliances (e.g. baby monitor, WiFi), security and navigation (e.g. radar and RFID) and especially in telecommunications (e.g. radio and TV broadcasting, mobile telephony). These developments mean that large parts of the global population are now exposed to RF-EMF and more will be exposed in the future. Concern has been raised regarding public health consequences from RF-EMF, and it is therefore crucial to perform a health risk assessment to support decision-makers and the general public.

The World Health Organization (WHO) has an ongoing project to assess potential health effects of exposure to RF-EMF in the general and working population. To prioritize potential adverse health outcomes from exposure to these fields, WHO conducted a broad international survey amongst RF experts in 2018 (Verbeek et al. 2021). Six major topics were identified (cancer, adverse reproductive outcomes, cognitive impairment, symptoms, oxidative stress, and heat-related effects) for which WHO has commissioned systematic reviews of observational and experimental studies to analyse and synthesize the available evidence. In this paper, we present the protocol for a systematic review of human observational studies on exposure to radiofrequency fields and risk of neoplastic diseases.

### 1.2 Description of the exposure

Human exposure to RF-EMF arises from a wide range of sources that use RF energy. Within the range of 100 kHz to 300 GHz, specific frequency bands are assigned to different applications (Table 1).

**Table 1:** Radiofrequency spectrum allocation (ITU 2020) and main applications.

Frequency band	Main applications
100–3000 kHz	Amplitude modulated (AM) radio broadcast, radionavigation, 160 m amateur radio, induction heaters, electrosurgical units
3–30 MHz	International broadcast, amateur and citizens band (CB) radio, dielectric heaters, shortwave diathermy
30–300 MHz	Frequency modulated (FM) radio broadcast, VHF television broadcast, mobile and handheld transmitters, cordless phones
300–3000 MHz	UHF television broadcast, G1-G5 mobile (cellular) phones and base stations, Digital Enhanced Cordless Telephones (DECT), Terrestrial Trunked Radio (TETRA), walkie-talkie, microwave ovens, microwave diathermy, air traffic radars, WiFi
3–30 GHz	Microwave relays, satellite uplinks, aircraft on-board radar, police radar, 5G mobile telephony, WiFi
30–300 GHz	Radio-astronomy, space-research, satellite, radionavigation

RF-emitting equipment or devices can operate either far or close to the body, resulting in far-field or near-field exposure conditions. RF-EMF can penetrate into the body and the absorption of RF energy may concern the whole body, or selected body parts. The level of human exposure to RF-EMF is affected by the strength of the incident field (which in turn depends on the source output power, the signal propagation pattern, and the distance from the source); the signal frequency (the higher the frequency, the lower the penetration depth), modulation and polarization; the exposure duration; and receiver factors such as body dimension, water content and dielectric characteristics of exposed tissues (Frei and Röösli 2014). The field strength decreases rapidly with distance (d) from a source, according to the inverse or the inverse square law (1/d or 1/d^2^), depending on the type of dispersion (directional or non-directional). In far-field conditions (occurring at a distance of about one wavelength from the source), compliance with safety limits is usually assessed through measurements of RF environmental levels, as electric field strength (E, in volt per meter, V/m) or, to sum up the strengths of RF signals at different frequencies, as incident power density (S, in watt per square meter, W/m^2^); in near-field conditions coupling into the human body occurs, and the specific energy absorption rate (SAR, W/kg) is the most relevant dosimetric unit (Wood 2017a). It is not possible to measure the SAR directly. The combined assessment of the total absorbed RF energy from far- and near-field sources requires dosimetric calculations. Few attempts to develop an integrated RF exposure index for epidemiological research have been undertaken to date (Lauer et al., 2013, Liorni et al., 2020, Roser et al., 2015). Modelled integrated “doses” of RF-EMF, along with the relative contribution of distinct sources to the whole body or brain RF energy absorption, have been calculated in children/adolescents (Birks et al., 2021, Cabré-Riera et al., 2020, Roser et al., 2017), and adults (van Wel et al., 2021). These estimates are context-specific, depending on use habits of wireless devices in the study population, and mobile network features in the study area during the observation period (Laurier and Röösli 2020). Nevertheless, the research conducted so far indicates that near-field sources are dominant contributors to both the brain and whole-body doses, with the largest contribution from mobile phone calls on GSM (2G) networks (Birks et al., 2021, van Wel et al., 2021).

For studies on RF-EMF and neoplasm risk, the ideal exposure assessment should capture all major sources of exposure for the pertinent part of the body, over the entire induction period and by susceptible time windows, taking into account time-related variations in source-specific exposure levels (SCENIHR 2015).

### 1.3 Description of the outcome

Neoplasia is a large family of diverse diseases with a common underlying pathology characterized by uncontrolled cellular growth and division (Dean and Moitra, 2018). Based on behaviour, primary neoplasms can be classified in two major groups: benign (or non-malignant) and malignant. Compared to benign tumours, malignant neoplasms (*syn*. cancer) show a greater degree of anaplasia and have the properties of invasion and metastasis. The current version of the International Classification of Diseases for Oncology (ICD-O, v 3.1) includes 77 major tumour sites and 47 major histologic types (Fritz et al. 2013). The International Classification of Childhood Cancer (ICC3) includes 12 main histologic groups and 47 subgroups (Steliarova-Foucher et al. 2005). The expanding knowledge on the molecular profile of cancer has led to novel classifications (Sherman et al. 2018).

Due to this heterogeneity, aetiological research focuses on distinct tumour types/subtypes.

Cancer is a major public health issue at the global level, with 24.5 million incident cases in 2017 (Global Burden of Disease Cancer Collaboration et al. 2019). It is the first or second leading cause of premature death (at ages 30–69 years) in 134 of 183 countries (Cao et al. 2020). Established lifestyle and environmental causes of cancer comprise tobacco smoke, alcohol consumption, physical inactivity, dietary factors including consumption of red and processed meat, obesity/overweight, cancer-causing infectious agents, ionizing radiation, UV radiation, exogenous hormones, and several occupational exposures (Wild et al. 2020). Up to 2019, the International Agency for Research on Cancer (IARC) Monograph Programme has classified 50 occupational agents or exposure circumstances as established (group 1) human carcinogens (Siemiatycki and Rushton 2020); cancers of the lung and other respiratory sites, followed by the skin, account for the largest proportion of neoplasms causally associated with these agents (Loomis et al., 2018; World Cancer Report, 2020).

### 1.4 Rationale for a systematic review

RF radiation is part of the non-ionizing region of the electromagnetic spectrum, which means that there is not sufficient energy in a single quantum of RF energy to ionize an atom or a molecule (Barnes et al. 2019). There is currently no established mechanism underpinning the potential carcinogenicity of RF-EMF at exposure levels below international standards (ICNIRP, 2020a, IEEE, 2019). The capacity of RF radiation to induce genetic damage or other cancer-related effects (Smith and Guyton 2020) has been assessed in several hundred experimental studies. The results of these studies are inconsistent (Miyakoshi, 2019, Wood, 2017b), with no dose–response and inverse correlations between effect size and study quality detected in two *meta*-analyses (Vijayalaxmi, 2019, Wood et al., 2021).

Independently of the pathogenesis, if exposure to RF-EMF increased the risk of cancer, then this would have serious public health consequences and require population-level preventive strategies, including a revision of the threshold-based limitation principle currently applied to non-ionizing radiation in the radiofrequency range (ICNIRP 2020b).

RF-EMF was classified by IARC as possibly carcinogenic to humans (group 2B), based on limited evidence in humans, limited evidence in experimental animals, and weak support from mechanistic studies (IARC 2013). The evaluation was driven by two large case-control studies showing positive associations between glioma and acoustic neuroma and wireless phone use (Baan et al. 2011). The IARC panel also examined studies of brain tumours, leukaemia/lymphoma, or other malignancies in relation to occupational or environmental RF exposure, and judged this evidence inadequate to formulate conclusions (IARC 2013).

The IARC Monograph on RF-EMF covers the literature issued by mid-2011. New relevant studies have been made available since then. Several expert panels performed updated reviews of this body of evidence (AGNIR, 2012, ANSES, 2013, ANSES, 2016, ARPANSA, 2014, CCARS, 2017, Demers et al., 2014, FDA, 2020, HCN, 2016, ICHENF, 2018, SCENIHR, 2015, SSM, 2013, SSM, 2014, SSM, 2015, SSM, 2016, SSM, 2017, SSM, 2018, SSM, 2019). Eleven *meta*-analyses addressing mobile phone use and head tumour risks were published since 2012 (Bortkiewicz et al., 2017, Choi et al., 2020, de Siqueira et al., 2017, Gong et al., 2014, Hardell et al., 2013, Lagorio and Röösli, 2014, Prasad et al., 2017, Repacholi et al., 2012, Röösli et al., 2019, Wang and Guo, 2016, Yang et al., 2017), often arriving at conflicting conclusions (Ioannidis 2018).

None of these evidence syntheses complies in full with the recommendations for the conduct of systematic reviews in toxicology and environmental health research (COSTER) (Whaley et al. 2020), and only one protocol (Mao et al. 2013) of a *meta*-analysis later published in Chinese (Gong et al. 2014) was preregistered in PROSPERO.

The need for a structured updated appraisal of this body of evidence is widely recognised. Non-ionising radiation (radiofrequency) is among the agents recommended with high priority for re-evaluation by the Advisory Group for the IARC Monographs during 2020–2024 (Marques et al. 2019). Two registered systematic reviews of epidemiological studies on RF-EMF and cancer are underway, focusing on exposures experienced by the general population (Farhat et al. 2020) and workers (Modenese et al. 2020). As systematic reviews cannot remedy limitations of the original studies, those (and our) syntheses are unlike to produce conclusive evidence. Nonetheless, all will advance knowledge on the topic to the extent they will transparently document the choices made at the critical stages of study selection, risk-of-bias assessment, data synthesis, and confidence in evidence rating.

## 2 Objectives

The overall aim of the planned systematic review is to assess the quality and strength of the evidence provided by human observational studies for a causal association between exposure to RF-EMF and risk of neoplastic diseases. The specific objectives are: (i) identify the relevant epidemiological literature; (ii) assess risk-of-bias for individual studies; (iii) synthesize the evidence on the exposure-outcome relationship (in terms of magnitude of effects and shape of exposure–response gradients), and evaluate heterogeneity in results across studies; (iv) rate confidence in the body of evidence.

No epidemiological study to date has investigated risk of neoplastic diseases in relation to individual exposure to RF-EMF from all exposure sources and settings (AGNIR, 2012, ARPANSA, 2014, IARC, 2013).

Therefore, we will separately review three bodies of evidence, addressing neoplasia risk in the general population in relation to RF exposure from near-field (SR-A) or far-field (SR-B) sources, and in working age individuals in relation to occupational RF exposures (SR-C). The scientific questions expressed as PECO statements (Morgan et al. 2018) are reported in Table 2.

**Table 2:** PECO statements.

**SR-A. Systematic review of studies on RF-EMF exposure from wireless phone use**	
**P**opulation	Members of the general population, without restriction on sex, age, or other individual characteristics.
**E**xposure	*Definition*: Near-field RF exposure from personal use of mobile or cordless phones, occurring prior to outcome, and based on indirect measures (subscriber status, self-reported history of mobile phone or cordless phone use), traffic data, or modelling.
	*Classification*: Ever exposed; time since first exposure; cumulative exposure level.
**C**omparator	Never or non-regular users of wireless phones.
**O**utcomes	*Critical***:** Glioma/brain cancer in adults; paediatric brain tumours; meningioma; acoustic neuroma; pituitary gland tumours; salivary gland tumours.
	*Important***:** Any other neoplasm.


**SR-B. Systematic review of studies on environmental exposures to RF-EMF**	

**P**opulation	Members of the general population, without restriction on sex, age, or other individual characteristics.
**E**xposure	*Definition****:*** Far-field RF exposure from radio-television transmitters, base stations or any other fixed-site transmitter, occurring prior to outcome, and based on measurements, modelling, or geocoded distance to the sources (the latter limited to broadcast transmitters).
	*Classification***:** Ever exposed; duration of exposure or time since first exposure; average or cumulative exposure level.
**C**omparator	No or low-level exposure from environmental sources of RF-EMF.
**O**utcomes	*Critical***:** Childhood leukaemia, paediatric brain tumours, glioma/brain cancer in adults, and leukaemia in adults.
	*Important***:** Any other neoplasm.


**SR-C. Systematic review of studies on occupational exposures to RF-EMF**	

**P**opulation	Occupationally active individuals, with no further restriction on sex, age, or other individual characteristics.
**E**xposure	*Definition***:** Near- or far-field RF exposure from professional use of hand-held transceivers or RF-emitting equipment in the workplaces, occurring prior to outcome, and based on measurements, estimates of exposure level from job- or source-exposure matrices (JEM, SEM), or indirect measures such job title or task (option limited to studies explicitly aimed at assessing the effect of exposure to well-characterized sources and types of RF-EMF).
	*Classification***:** Ever exposed; exposure frequency; exposure duration or time since first exposure; average or cumulative exposure level.
**C**omparator	No or low-level occupational exposure to RF-EMF.
**O**utcomes	*Critical***:** Glioma/brain cancer, leukaemia.
	*Important***:** Any other neoplasm.

## 3 Methods

We will follow the WHO approach to guideline development (WHO, 2014a), complemented by recent guidance in systematic reviews of observational studies of aetiology and environmental hazards (Dekkers et al., 2019, NTP-OHAT, 2019). COSTER was referred to in planning the systematic review (Whaley et al. 2020). The review team includes members trained in systematic review methodology; competence in RF-EMF exposure assessment; expertise in epidemiological, statistical and *meta*-analytical methods; as well as long-term experience in conducting epidemiological studies of carcinogenic hazards from RF-exposure sources relevant to the general population and to workers. The literature search strategies were refined by an expert in information science (JE, see Acknowledgments). The protocol has been registered in PROSPERO (CRD42021236798). Findings from the systematic review will be reported in accordance with the updated PRISMA guidelines for reporting systematic reviews (Page et al. 2021b), and this protocol conforms to the PRISMA-P guidelines for systematic review protocols (Shamseer et al. 2015). The L3-PRISMA Report for systematic review protocols submitted to *Environment International* is enclosed (**Annex** **1**). In case any amendments to this protocol are made during the review process, changes and related reasons will be reported in the final article.

### 3.1 Eligibility criteria

#### 3.1.1 Types of populations

The SR-A and SR-B will focus on members of the general populations, and SR-C on occupationally active individuals. We will not apply restrictions on sex, age, or other individual characteristics.

#### 3.1.2 Types of exposures

Given the lack of a known biological mechanism for a potential carcinogenic effect of RF-EMF, it is unknown which aspect of the exposure may be biologically relevant. Therefore, the choice of the exposure metrics of priority interest is informed by contextual evidence relevant for the types of RF exposure considered in each component of the systematic review, summarized below.

##### 3.1.2.1 Wireless phones

Mobile phones are the most common type of wireless phones and their use is now universal, with 8.3 billion subscriptions in 2019 (ITU 2019).

So called bag-phones and car-phones were introduced at the beginning of the 1980s, but RF exposure to the head from these devices was very low, and it is considered irrelevant to the aim of SR-A.

Handheld mobile phones (analog, 450 MHz or 800/900 MHz) have first been available in 1984 in the Unites States, and since 1987 in the Nordic countries. Subsequent generations of digital mobile phones were introduced approximately every tenth year: 2G (GSM 900/1800 MHz) in early 1990s, 3G (UMTS, 1900  MHz) in early 2000s, 4G (LTE, 800/2600 MHz) in early 2010s.

Mobile phone-related exposure to RF-EMF above 6 GHz will occur at full deployment of 5G networks. Given the short time period since the introduction of this technology, we do not expect to identify studies addressing the association between 5G mobile phone use and neoplasia risk. However, epidemiological studies of radar workers exposed to RF-EMF > 6 GHz have been conducted (Karipidis et al. 2021) and will be considered for inclusion in SR-C (see § 3.1.2.3).

The exposure of interest for tumours in the head region consists of RF energy emitted by handheld mobile phones during voice calls, with the device in contact with the head. Communication and data transfer from/to devices is established and regulated by base stations. The periodic signals for location update and possible traffic occurring when the device is in stand-by mode (Hansson Mild et al., 2012, Urbinello and Röösli, 2013) are not relevant for exposure to the head because the phone would usually not be held next to it (AGNIR 2012).

Mobile phones, when held to the ear, are typical sources of near-field exposure to RF-EMF, highly localized to the tissues nearest the transceiver, with a marked attenuation through the head (Cardis et al., 2008, Dimbylow and Mann, 1994, Dimbylow and Mann, 1999). The local (brain) SAR during each call depends on several parameters, including features of the phone, factors affecting the device transmission power, user’s physical characteristics, and usage modalities (how the phone is held towards the ear; use of hands-free devices). Most of these parameters cannot be measured over the long exposure time window of interest for epidemiological studies investigating the effect of mobile phone use on neoplasia risks; thus, it is not possible to arrive at an estimate of the exposure in terms of SAR. Actually, all studies relevant for the current systematic review have used indirect measures of exposure, based on self-reported histories of mobile phone use or on mobile phone subscriber lists (Deltour and Schüz 2014). Estimates of absorbed RF energy were used in two studies (Cardis et al., 2011a; Takebayashi et al., 2008); the modelled brain absorption was based on various input variables, but the self-reported call time was the dominating parameter, while other factors gave a very small contribution to variation in estimated values (Cardis et al., 2011b).

This systematic review will summarize the evidence for the exposure variables most commonly used in the scientific literature: ever use of mobile phones, time since start of mobile phone use, cumulative hours of mobile phone use, and cumulative number of calls. Each of these exposure indicators has advantages and disadvantages, and will complement each other in the overall assessment of an effect.

The variable “time since start of mobile phone use” (also called “time since first use”) is a crude measure, but it takes into consideration the tumour latency (which may vary between tumour types), and allows an appropriate assessment of the external validity when comparing results of the analytical studies with incidence time-trend studies of the investigated tumours (see § 3.1.5.3).

In addition, measurement errors accumulate. Therefore, time since start use (based on date of first use, a single event occurring once) is likely to be affected by information bias to a lesser extent than cumulative exposure indices usually derived from multiple time-varying self-reported variables. There was little evidence of differential recall errors in validation studies of date of start/time since start use reported by cases and controls (Aydin et al., 2011a, Aydin et al., 2011b, Pettersson et al., 2015).

The variables “cumulative hours of mobile phone use”, and “cumulative number of calls” provide better estimates of the total amount of mobile phone use, but are more greatly affected by recall bias (Aydin et al., 2011b, Vrijheid et al., 2009a) because past intensity of use is more difficult to recall than current use, especially as mobile phone habits have changed considerably over time.

The adequacy of amount of mobile phone use as an indicator of exposure to RF energy emitted by the device has decreased over time, due to several factors. In early 2000s, call time and measured output power of the phone were fairly well correlated (Berg et al. 2005). This occurred because GSM (2G) phones operated at the maximum power for about 40% of call time, likely due to sub-optimal efficiency of the mobile networks (Vrijheid et al. 2009b). The adaptive power control in response to the network quality has notably improved in 3G and 4G systems, the density of base stations has increased, and the average output power per call has decreased. Based on data reviewed in (Joshi et al. 2020), the average output power per call of mobile phones in 2G networks is around 50 times higher than with 3G/4G technologies (50% *vs* 1% of maximum power). A similar ratio between the contributions of 2G and 3G phone calls to the total whole brain dose (50% *vs* 0.8%) was obtained in a multi-country European study, combining source-specific SAR estimates (Liorni et al. 2020) with population data on usage patterns (time and emitted output power) per source (van Wel et al., 2021). Using country-specific estimates of comparative output power levels (Persson et al. 2012), a higher relative weight (150:1) was assigned to the prospectively recorded fractions of total call time on 2G *vs* 3G networks in a recent analysis of data from the Swedish and Finn COSMOS cohorts (Auvinen et al. 2019). Drawing upon the above estimates, a user of a 3G/4G phone would have to achieve a call time 50 to 150 times as long as a 2G phone user to get the same accumulated energy deposition. However, old and new networks coexist for some time, new generation devices can usually connect to previous generation base stations, and stopping use of mobile phone is uncommon. Without recorded data on actual network used during phone calls, it is impossible to develop cumulative exposure indices properly accounting for system-specific exposure levels. Multiple counting of individual data is an issue in subgroup analyses based on device features (make and model). Analyses by cumulative amount of use stratified by recency of start use may be informative in cohort studies with prospective exposure assessment but not in case-control studies, where the greater proneness to information bias would hamper the interpretation of results.

The preferred side of the head for mobile phone use is an important exposure determinant but, when assessed retrospectively through self-report, is affected by substantial misclassification.

Consistent support to this claim comes from several validation studies that used software applications to record the side of mobile phone use (Goedhart et al., 2018, Goedhart et al., 2015, Inyang et al., 2010, Kiyohara et al., 2018, Kiyohara et al., 2016). In a sample of over 200 volunteers from 12 countries, the agreement between self-reported and recorded laterality was very poor (concordance = 59% for declared mainly right-side users, and 43% among left-side users), and there was evidence of systematic errors, with a laterality agreement odds ratio of 0.48 for users in the > 80^th^ percentile of recorded amount of use compared to users < 20^th^ percentile (Goedhart et al. 2018). A progressive deterioration of the agreement between self-reported and recorded laterality over time (weighted k = 0.667 at the first interview, 0.527 after 10–12 months, and 0.437 after 48–55 months) was detected in a similar validation study from Japan (Kiyohara et al. 2018).

Self-reported laterality is also considerably affected by recall bias, as indicated by concurrent observations of increased risk for ipsilateral mobile phone use and protective effect for contralateral use (Schüz, 2009). Due to such a poor validity, self-reported laterality of mobile phone use is not included among the exposure metrics and contrasts examined in SR-A (Table 2).

Cordless phones are another source of near-field exposure to RF-EMF. The most common technology is Digital Enhanced Cordless Communication (DECT), which uses time sharing and pulse modulated signals. DECT phones have a peak power of 250 mW, operate with 400 μs bursts every 10 ms (4% duty factor), and have an average output power of 10 mW (SCENIHR 2015). It is worth noting that the transmission power of cordless phones is 1–2 orders of magnitude lower than that of 1G-2G mobile phones (Lauer et al. 2013). RF-exposure from cordless phones can only be assessed based on indirect measures (prevalence, amount and duration of use), and there are no objective sources of data against which self-reported information can be validated.

##### 3.1.2.2 Environmental sources

In SR-B, we will include studies addressing neoplasm risks in relation to RF exposure from radio and television masts, base stations or any other fixed-site transmitter. In principle, the average or cumulative whole body SAR is the exposure measure of interest. As the SAR cannot be directly measured, epidemiological studies have usually relied on measured or modelled levels of electric fields, magnetic fields or power density at the subjects’ residence (less often also at schools), or on crude exposure proxies such as distance to the exposure source.

For a given transmitter, the electric field decreases in the beam with 1/distance from the source. Provided that the distance is objectively recorded (e.g., derived from geocodes), distance from the source may be informative for antennas with a roughly isotropic transmission pattern. This is usually the case for large broadcast transmitters, although special care must be taken when different transmitters are included in the same study (Schmiedel et al. 2009). On the contrary, distance from a base station is a poor indicator of exposure to RF-EMF indoors, due to the complex propagation characteristics of emissions from base station antennas, including shielding effects and multiple reflections from house walls and other buildings (Frei et al. 2010).

We will restrict eligibility for inclusion to studies based on objective exposure indicators, such as measurements, modelling, or geocoded distance to a broadcast transmitter (but not to a mobile phone base station). Studies based on self-estimated distance to an antenna will not be included, as self-reported distance to transmitters is strongly affected by risk perception (Martens et al. 2017) and cannot be considered a reliable exposure indicator.

The preferred exposure index will be the E field strength in V/m, which is the unit used by the International Commission on Non-Ionizing Radiation Protection to express reference values (ICNIRP 2020a). Other exposure units such as the magnetic field strength in ampere per metre (H, A/m) or the incident power density (S, in W/m^2^) can be easily converted to V/m applying the plane-wave model (S = EH = E^2^/377 = 377H^2^), which is valid for far field exposure situations.

We will focus on differences in exposure level (using categorical or continuous exposure data), and according to exposure duration.

##### 3.1.2.3 Occupational exposures

Previous reviews of epidemiological studies of cancer risk in relation to occupational RF exposure have considered the evidence uninformative due to inconsistent results across studies affected by severe limitations in exposure assessment, and uncontrolled confounding (AGNIR, 2012, Feychting et al., 2005, IARC, 2013, Swerdlow, 1999). Most studies conducted so far used job-titles as exposure surrogates. Therefore, bias in study identification due to selective mention of RF exposures for occupations found at increased cancer risk, was an additional concern in these reviews.

Some studies improved on exposure characterization by using expert assessment and job- or source-exposure matrices (JEM, SEM). Existing JEMs of occupational RF exposure (Karipidis et al., 2008, Kauppinen et al., 1998, Migault et al., 2019, Siemiatycki and Lavoué, 2018), provide exposure estimates often based on a small number of measurements per source and/or job, and may not be informative about the probability of exposure per occupation, the typical exposure of workers in specific jobs, and the variability of exposure levels by task, working practices, and over time. These drawbacks also apply to the most recent RF-JEM (Migault et al. 2019), comprising 282 occupational titles, and built by combining source-based measurements from the literature (Vila et al. 2016) with occupational data collected in the INTEROCC case-control study (≈ 9,300 participants from seven countries).

Actually, wide variations in exposure levels across and within jobs entailing use, operation or maintenance of RF-emitting equipment and devices, were observed in a large survey (≈ 4,300 measurements from ≈ 900 RF sources in over 200 workplaces) carried out in Israel in 1995–2005 (Hareuveny et al. 2015).

A consequential option would be to restrict inclusion in the current review to occupational studies with measurement-based assessment of exposure to RF-EMF at the individual level. This would drastically reduce the size of the available dataset. It might also result in excluding potentially informative longitudinal studies of occupational groups with high probability and/or intensity of RF-exposure, and limited co-exposures to established carcinogens. To identify occupations meeting these requirements, we selected the activities with a yearly cumulative exposure ≥ 250 W/m^2^ hour from the Israeli measurement survey (Hareuveny et al. 2015), and the job titles with an exposure probability > 20% from the INTEROCC JEM (Migault et al. 2019).

The resulting occupation-source matches (Table 3) are tentative and possibly inaccurate.

**Table 3:** Variability and cumulative level of exposure to RF-EMF in working activities with/nearby specific sources from the Israeli measurement survey (Hareuveny et al. 2015), and probability of exposure to RF-EMF per occupation from the INTEROCC JEM (Migault et al. 2019) tentatively matched to an exposure source assumed dominant.

ISRAELI MEASUREMENT SURVEY					INTEROCC JEM		
Exposure source (job/task)	Exposure variability			Cumulative exposure	Occupation (ISCO-88)	Exposure probability	
	N°_S_	N°_M_	GSD			E^+^	%
Walkie-talkie and other hand-held transmitters (drivers, security guards, police officers, others)	6	34	20.0	4460	5163-Prison guards	13	61.9
					5162-Police officers	66	46.8
					5169-Protective service workers	55	33.9
					5161-Firefighters	8	30.8
Dielectric heating-plastic (plastic welders)	55	143	31.6	2180	8232-Plastic products machine operators	3	3.2
Induction heating-metals (various applications)	23	63	6.3	1400	7213-Sheet metal workers	8	3.8
Heating, thawing, drying (many industries)	9	49	6.3	620	8266-Shoemaking & related machine operators	3	5.0
Diathermy (physiotherapists)	16	43	31.6	500	3226-Physiotherapists & associate professionals	16	20.5
High power transmitters (operation & maintenance)	62	197	31.6	250	3521-Broadcasting & audiovisual technicians	–	–
					7422-ICT installers & servicers	–	–
Marine radar (on board personnel)	7	35	6.3	13	3142-Ships’ deck officers & pilots	13	65.0
Ground airborne radar (operators)	39	25	3.2	4	3144-Air traffic controllers	8	44.4

**N°_S_** = number of distinct RF-emitting equipment/devices; **N°_M_** = number of measurements of the electrical (E) field level at the worker location (at the head for walkie-talkie) in typical working conditions; **GSD** = geometric standard deviation of the average incident power density (S, W/m^2^) per source; **Cumulative exposure** (W/m^2^ h/year) = average level of exposure per hour (S, W/m^2^) times the hours worked in a year (derived from publically available data and workplace interviews); **E^+^** = numbers of subjects in the index occupation who reported exposure to one or more sources of RF-EMF.

In fact, INTEROCC participants reported work with/nearby 1.3 RF-sources on average (Vila et al. 2018). An example is police officers who may be exposed to RF-EMF from hand-held transceivers, vehicle-mounted and stationary communication systems, and short range radar. Several occupational titles classified in the highest decile of RF exposure level of the JEM were not easily traceable to a particular source of exposure (e.g., Production and operations department managers in personal care, cleaning and related services; Estate agents; Travel agency and related clerks). Workers engaged in operation and maintenance of high power radio-TV antennas (ISCO-88 codes 3521, 7422) are not reported by (Migault et al. 2019), likely due to the rarity of these occupations among study participants.

We also developed additional literature search strategies to identify epidemiological studies of cancer in relation to several occupations listed in Table 3 (those with exposure probability > 20%, plus workers involved in operation and maintenance of broadcasting antennas), without mentioning radiofrequency fields or microwaves (see **Annex** **2**, **§ 4–5**). The large majority of 76 potentially relevant papers identified, concerned firefighters (n. 44) and police officers (n. 12).

We concluded that, for most occupations in Table 3, studies relying on job titles as the only exposure surrogate would be uninformative to the aim of current review, due to either a low exposure probability (e.g., occupations possibly entailing exposure from industrial heating equipment or broadcast transmitters, and physiotherapists); a low level of over-background exposure to RF-EMF (ships’ deck officers & pilots and air traffic controllers); or common and relevant co-exposure to known and suspect carcinogens, in spite of a high probability and intensity of RF exposure. The latter category includes firefighters, exposed to several carcinogens in combustion products and to diesel engine exhausts (Casjens et al., 2020, Jalilian et al., 2019). Studies of police officers without a detailed assessment of exposures to RF-EMF and potential confounders would also be problematic, because of co-exposures to traffic-related air pollutants, UV radiation and shift work (Wirth et al. 2013).

In summary, we will include studies investigating neoplasia risk in relation to exposure to RF-EMF from professional use of hand-held transceivers, or from RF-emitting equipment in the workplace, with exposure assessment based on measurements or estimates of exposure level derived from JEM or SEM. We will also consider eligible for inclusion studies with indirect measures of exposure (job title or task), provided that the assessment of the effect of RF-EMF exposure was a predefined research objective, the exposure is well characterized in terms of source and type (equipment/device, frequency band, power), and the requirements concerning the exposure contrasts (§ 3.1.3) are met.

We will exclude studies based on self-reported exposure only (i.e., without information on job, task and/or exposure source). We will also exclude studies addressing occupations where exposures to electric and magnetic fields between 0 Hz and 10 MHz are dominant compared to the co-occurring exposure to RF-EMF (e.g., MRI machine operators, arc-welders, or electricity production and distribution workers), or with dominant exposures to established carcinogens (e.g., firefighters).

The priority exposure classifications will be ever *vs* never exposed, exposure frequency, exposure duration or time since first exposure, average or cumulative exposure level.

#### 3.1.3 Types of comparators

To be eligible for inclusion, studies must allow comparing the occurrence of the outcome between exposed and unexposed subjects, or between at least two groups with different exposure frequency, intensity, duration, time since first exposure, average or cumulative exposure level.

#### 3.1.4 Types of outcomes

##### 3.1.4.1 Critical and important outcomes

According to COSTER recommendation 1.3.4, the eligibility criteria for outcomes should clearly “define as relevant to review objectives the primary and secondary outcomes of interest (including defining which are apical and which are intermediate), what will be acceptable outcome measures (e.g. diagnostic criteria, scales) and the timing of the outcome measurement.” (Whaley et al. 2020).

Distinguishing outcomes of primary and secondary relevance to the objectives of this systematic review is challenging. Any neoplasm is in principle important in the assessment of carcinogenic hazards. However, the multiplicity and diversity of tumour types precludes the possibility of setting exhaustive eligibility criteria at the required level of detail, and hinders the identification of the critical confounders for all exposure-outcome pairs of potential interest. Moreover, large numbers of outcomes can make reviews unfocussed, unmanageable for the user, and prone to selective outcome reporting bias. On these grounds, the Cochrane collaboration maintains that the predefined critical outcomes should be as few as possible; additional important outcomes may be specified, but up to seven outcomes will form the basis of the GRADE assessment [MECIR standards C14 (Higgins et al. 2020)].

While no eligibility restriction on tumour type will be applied, we will focus on six neoplasms: brain tumours (including gliomas and other histotypes); meningioma; acoustic neuroma; pituitary tumours; salivary gland tumours; and leukaemias (including several subtypes). In the lack of guiding biological hypotheses, the choice of these “critical” outcomes relied on contextual evidence: type of exposure (near-field, far-field), knowledge about exogenous risk factors for specific neoplasms (favouring tumours with poorly understood aetiology), and available study data (prioritizing tumours most commonly investigated in relation to RF-EMF, based on previous reviews).

Table 4 reports the standard nomenclature and codes of these tumours according to the ICD-10 and ICD-O-3 classifications (Fritz et al., 2013, WHO, 2016). These details are given for illustrative purposes, reminding that clinical and aetiological disease definitions often diverge (Olsen 2012).

**Table 4:** Neoplasms of primary interest: ICD-10 and ICD-O-3 codes.

Neoplasm		ICD-10*	ICD-O-3	
			Site	Histology / behaviour
Brain, malignant^†^ (*syn*. brain cancer)		C71	C71	8020/3, 8440/3, 8680/3, 8693/3, 8963/3, 9060/3, 9061/3,9064/3, 9065/3, 9070/3, 9071/3, 9072/3, 9080/3, 9081/3, 9082/3, 9083/3, 9084/3, 9085/3, 9100/3, 9101/3, 9364/3, 9380/3, 9381/3, 9382/3, 9390/3, 9391/3, 9392/3, 9393/3, 9400/3, 9401/3, 9410/3, 9411/3, 9420/3, 9421/1, 9423/3, 9424/3, 9425/3, 9430/3, 9440/3, 9441/3, 9442/3, 9450/3, 9451/3, 9460/3, 9470/3, 9471/3, 9472/3, 9473/3, 9474/3, 9480/3, 9490/3, 9500/3, 9501/3, 9502/3, 9505/3, 9508/3, 9522/3, 9523/3
Brain, non-malignant† (*syn*. brain tumours)		D33.0-D33.2		8440/0, 8680/1, 8681/1, 8690/1, 8693/1, 9080/0,1, 9084/0, 9363/0, 9390/1, 9383/1, 9384/1, 9394/1, 9412/1, 9413/0, 9444/1, 9442/1, 9490/0, 9492/0, 9493/0, 9505/1, 9506/1, 9509/1
Brain, uncertain behaviour		D43.0-D43.2		–
Gliomas§		C71	C71	9380–9384, 9391–9460
Astrocytomas, low-grade (I-II)				9384, 9400, 9421, 9424, 9425
Astrocytoma, anaplastic (III)				9401
Glioblastoma (IV)				9440, 9441
Oligoastrocytomas (II-III)				9382
Oligodendroglioma (II-III)				9450, 9451
Other gliomas (I-II)				9431, 9444
Glioma, malignant NOS				9380
Meningioma, malignant (rare)		C70	C70	9530/3, 9538/3
Meningioma, non-malignant†		D32.0		9530/0, 9530/1, 9531/0, 9532/0, 9533/0, 9534/0, 9535/0, 9537/0, 9538/1, 9539/1
Cerebral Meninges, uncertain behaviour		D42.0		–
Acoustic neuroma (*syn*. vestibular schwannoma)		D33.3	C72.4	9560
Pituitary gland, malignant (rare)		C75.1	C75.1	8272/3
Pituitary gland, benign		D35.2		8272/0
Salivary glands (*incl*. Parotid), malignant		C07-C08	C07-C08	8272/0, 8561/0 (major types)
Salivary glands (*incl*. Parotid), benign		D11		8272/3, 8430/3 (major types)
Leukaemias		C91-C95	C42.1	9800–9948
Lymphoid		C91		981–983
Myeloid		C92		984–993
Other of specified cell type		C93-94		994
Unspecified cell type		C95		980

*The ICD-10 classification of neoplasms is based on site and behaviour categories: malignant (C00-C97), in situ (D00-D09), benign (D10-D36), uncertain/unknown behaviour (D37-D48). The ICD-10 terms D42.0, D43.0-D43.2 have no equivalent codes in ICD-O-3.

† Paediatric brain tumours include histotypes uncommon in adults, such as germ cell tumours (8020, 8440, 9060–9061, 9064, 9065, 9070–9072, 9080–9085, 9100–9101), pilocytic astrocytoma (9421, 9425), ependymal tumours (9383, 9391–9394), embryonal tumours (8963, 9364, 9470–9474, 9480, 9490, 9500–9502, 9508), medulloblastoma (9470–9472, 9474), and primitive neuroectodermal tumours (9473).

§ The main subtypes of gliomas are reported below, with the WHO grade for neoplasms of the central nervous system (Louis et al. 2007) in brackets. Grade I are the least aggressive and grade IV the most aggressive tumours.

##### 3.1.4.2 Diagnostic methods and measures of occurrence

Eligibility for inclusion in the critical outcome subset is restricted to studies including newly diagnosed (incident) cases of the diseases of interest, either histology-confirmed or based on unequivocal diagnostic imaging (the latter criterion only applies to CNS tumours), ascertained through cancer registries, hospitals, or other sources with adequate coverage of the study base during the observation period. We will exclude studies based on self-reported outcomes, as well as on hospital admissions only (due to uncertainties about the date of diagnosis). Information from death certificates only is considered the least valid basis of diagnosis for neoplasms (Jensen et al. 1991). Studies based on cancer-related causes of death are eligible for inclusion in the “important” outcome subset, conditionally on the study design (see § 3.1.5 and § 3.1.6).

#### 3.1.5 Types of studies

##### 3.1.5.1 Inclusion criteria

Eligibility for inclusion is restricted to aetiological studies of cohort and case-control design, comprising all typologies considered by (Gail et al. 2019). We will assess compliance with the eligibility criteria based on standard definitions (Elwood, 2017, Porta, 2016), rather than on the terminology used by the publication authors. If the measures of effect are based on cancer mortality, eligibility for inclusion is further restricted to cohort and cohort-nested case-control studies; population-based case-control studies with deceased cases and controls will not be included, because this study design renders the identification of the study base difficult when not impossible.

##### 3.1.5.2 Exclusion criteria

Case reports and case series are ineligible for inclusion due the lack of a control group. We will also exclude comparative studies such as ecological studies (geographical correlation and time-trend analyses), cross-sectional studies, and case-case analyses of case-control studies, because these study designs do not allow calculating the intended measures of effect (see § 3.1.6). For example, case-only studies are ineligible for inclusion because in this study design there are no study subjects (exposed or unexposed) who have not experienced the outcome, and the resulting measure of effect is not an estimate of the disease incidence rate ratio in the source population.

##### 3.1.5.3 Complementary evidence

In line with the triangulation approach (Arroyave et al., 2021, Lawlor et al., 2016, Steenland et al., 2020), we will systematically search for and include three categories of complementary evidence: (a) exposure validation and other bias studies conducted in the framework of included studies, or directly relevant to the investigated exposure-outcome pairs; (b) source-specific RF dose-modelling; and (c) simulation studies based on incidence time trends of specific types of CNS tumours.

Findings from the first study group will be considered in risk of bias assessment (see **Annex 4**, **§ II.4** and § II.5), and those from the second group at the stage of quality of evidence rating (see **Annex 6**, p. 7). The intended uses of data from the third group, in line with COSTER recommendation 7.8 to interpret the external validity of the overall body of evidence (Whaley et al. 2020), is described below.

Monitoring of incidence rates over time allows investigating changes in disease patterns that affect specific birth cohorts, vary with age, or exhibit calendar effects (which can occur if exposures are localized in time and affect large segments in the population at once), and has substantially contributed to current knowledge about environmental causes of cancer (Olsen 2012).

Regarding the possible carcinogenicity of RF-radiation at exposure levels below international guidelines, analyses of cancer incidence time trends are considered informative owing to the steep increase in mobile phone use (and related changes in prevalence and level of RF exposure to the head) since mid-1990s, along with the limited number of known competing environmental risk factors for glioma and other intracranial tumours (Olsen, 2012, Röösli et al., 2019, WHO, 2010).

The availability of high quality registry data with virtually complete tumour registration over long time periods, is a prerequisite for conducting these studies.

Time-trend analyses of CNS tumours are prone to bias. “Apparent” changes in incidence rates over time (i.e., not reflecting true changes in incidence) may result from demographic changes, and/or changes in sensitivity and accessibility of imaging techniques, in histologic classification, and in registration procedures (Ostrom et al., 2020). The latter is especially applicable to the collection of non-malignant brain tumours, meningioma and other benign CNS tumours (Dolecek et al., 2015, Withrow et al., 2021). Detection bias is an additional concern in time-trend analyses of acoustic neuroma incidence rates (Reznitsky et al. 2019). On these grounds, we will only consider “simulation studies”, purposely planned to assess the external plausibility of findings from analytical studies of specific CNS tumour risks in relation to mobile phone use, by comparing predicted and observed time-trends of incidence rates. To date, studies of this type have been conducted for malignant brain tumours in the whole (Chapman et al., 2016, Sato et al., 2019); for gliomas (de Vocht, 2016, de Vocht, 2017, de Vocht, 2019, Deltour et al., 2012, Karipidis et al., 2018, Karipidis et al., 2019, Little et al., 2012, Villeneuve et al., 2021); for glioma subtypes [astrocytoma (Little et al. 2012); glioblastoma multiforme (de Vocht, 2016, de Vocht, 2019)]; and for multiple histotypes of malignant and benign tumours in the temporal lobe (de Vocht 2019). We intend using findings from these studies to set a range of “implausible sizes” for the measures of effect reported by the glioma/brain cancer studies considered in SR-A. These “credibility benchmarks” would be defined for RR estimates either above or below the null, at increasing intervals of time since first use and at increasing amount of use, overall and within specific time‐windows.

We will assess comparability of findings across simulation studies in terms of:

• Setting (country, population demographics, time period);
• Risk scenarios (measures of effect; effect size; latency periods; effect modifiers);
• Exposure (data used to model changes in mobile phone use in the target population);
• Outcome (anatomical site, histology, grade);
• Statistical methods;
• Predicted events (number of cases, incidence rates, percent rate changes, others).

If feasible, the results of multiple simulation studies per brain tumour category/type/subtype will be standardized to a common metric and *meta*-analysed. The study classification based on the external plausibility of the observed RR point estimate, will serve three purposes: (i) to validate the capacity of our customized RoB to distinguish studies at high and low risk of directional biases (§ 3.5.1); (ii) to assess the influence of studies reporting implausible measures of effect on the main meta-analyses’ results (§ 3.7.2); (iii) to inform the appraisal of the evidence strength (§ 3.9; **Annex 6**, **§ 3**).

##### 3.1.5.4 Years considered

No filter on publication date will be applied (see § 3.2 for time coverage of the search strategy).

##### 3.1.5.5 Publication language

We expect a large English literature base for the topic of the current review. We will not exclude any article based on language, but the search queries will include English terms only. When screening articles for inclusion, publications in languages other than the ones spoken by the reviewers (Greek, English, French, German, Italian, Portuguese) will be translated into English using Google Translate (https://translate. google.com/). All potentially relevant papers where we are in doubt about inclusion after automatic translation, will be translated to English by a human translator.

##### 3.1.5.6 Publication types

We will include peer-reviewed journal articles reporting original data from eligible study types. We will consider indexing in Medline as evidence of peer-review status. We will exclude reviews, *meta*-analyses, conference papers and proceedings, editorials, comments and letters, with the exception of correspondence related to the included studies (such as letters by the authors reporting errors in the published analysis, providing more detailed or extended data analyses, or discussing study strength and biases).

#### 3.1.6 Types of effect measures

We will focus on studies reporting incidence-based estimates of the relative risk of disease conditional on the exposure: rate ratio (RR) or hazard ratio (HR) in cohort studies and odds ratios (OR) in case-control studies. Because of the rarity of the neoplasms of interest, the HR and the OR can be considered equivalent to a RR (Higgins et al., 2021a). Moreover, possible *meta*-analyses will be performed on log-transformed measures of effect and confidence limits (CLs).

As anticipated, cohort (and cohort-nested case-control) studies with mortality-based estimates of relative risk (e.g., standardized mortality ratios – SMR), regardless the type of tumour investigated, will be included in the “important” outcome subset of the systematic review.

### 3.2 Information source and search strategy

Eligible studies will be identified by literature searches through Medline and Embase. We will also consult the EMF Portal (https://www.emf-portal.org/en), a dedicated database of the scientific literature on the health effects of exposure to electromagnetic fields, with documented high coverage of the topic (Drießen et al. 2017). The search timeframe (as in-print publication) will extend from the database inception dates (1946 for Medline; 1947 for Embase) to 31 December 2020 or to the date of the actual literature searches, whichever comes later. To comply with the MECIR requirement and COSTER recommendation 2.7 to update the searches within 12 months before publication of the review (Higgins et al., 2020, Whaley et al., 2020), we will re-run all searches shortly before the final analyses, and any further relevant studies identified will be retrieved for inclusion.

The Medline and Embase queries are reported in **Annex** **2, § 3**–**4**. The search on EMF-Portal will take advantages of the in-built facilities; to identify cohort, case-control and simulation studies, we will toggle “Epidemiological studies” (as Topic), and “Radio frequency (≥10 MHz)” or “Mobile communications” (as Frequency range), with “cancer” OR “tumour” as keywords; for exposure validation and dosimetry studies, we will select “Technical/dosimetric studies” and the above frequency ranges. As an additional source, we will use a library of over 400 “seed” studies (see **Annex 2, § 1,** Table 1), taken from the reference lists of 19 recent comprehensive reviews (AGNIR 2012; ANSES 2013; 2016; ARPANSA 2014; CCARS 2017; Demers et al., 2014, FDA, 2020, HCN, 2016, IARC, 2013, ICHENF, 2018, SCENIHR, 2015, SSM, 2013; 2014; 2015; 2016; 2018; 2019; WHO, 2014b).

We used this library to calibrate and assess the performance of draft Medline queries, intentionally designed to privilege sensitivity over precision (0.89 *vs* 0.09, in the final draft; **Annex 2, § 1,** Table 2).

The reference lists of included studies will be hand-searched for unidentified relevant publications.

Unpublished studies will not be sought. We will not search grey literature, defined as “all types of material not published commercially” (Alberani et al., 1990, The New York Academy of Medicine, 2016). We acknowledge that this might result in a “grey literature bias”, whereas studies yielding smaller and/or statistically nonsignificant effects might be less likely to be published and only available in PhD theses, conference proceedings, books, personal communications, and other forms of grey literature (Song et al., 2010). By definition, it is doubtful that systematic reviews can ever get a complete or representative set of this literature. This is especially applicable to aetiological studies, as most dedicated collections focus on health interventions (UIC University Library, 2021). More importantly, while the common occurrence of grey literature bias was fully supported by a *meta*-research study of over 3,000 *meta*-analyses from a wide range of scientific disciplines, the estimated effect size was very small [−0.092 (95% CI −0.143, −0.041)], and far below the impact of the “small study effects” [0.197 (95% CI 0133, 0.264)], acting in the opposite direction (Fanelli et al. 2017).

Eventually, part of the possibly relevant grey literature will be covered by the literature search through Embase, that includes over 3.6 million conference abstracts (Elsevier 2020).

### 3.3 Study selection

We will use the EndNote 20 software for the assemblage of the results of the literature searches, duplicate removal, and data management during the study selection process (Bramer et al., 2017, Peters, 2017). According to a recent comparative study of systematic review automation software packages, EndNote ranked highest for usability and acceptability (Cleo et al. 2019).

We will categorize the identified records by coherence with the subject of the systematic review and other features relevant to assess compliance with the predefined inclusion/exclusion criteria. This categorization will occur at the title/abstract or full-text levels of the review, as appropriate.

Two reviewers (DB, MSP) will independently assess the relevance of the identified articles, and their eligibility for inclusion in any of three systematic reviews (§ 3.3.1). Then, both reviewers will share their EndNote libraries with two other team members (KK, SL) who will revise and finalize the selection of studies (§ 3.3.2) for the three components of the systematic review.

All four reviewers, provided with written instructions on categorization scheme, variable coding, and treatment of multiple publications per study, will participate in a pilot testing of the study selection procedures undertaken on a small subset of the references retrieved.

#### 3.3.1 Selection of eligible articles

At the title/abstract screening step, all records will be classified in four categories of relevance (0 = Irrelevant; 1 = Relevant 8 = Complementary evidence; 9 = Unclear relevance). Examples of records amenable to exclusion at this stage include instances when the title/abstract/keywords fields provide clear evidence that the publication addresses: topics other than adverse health effects of RF fields (e.g., RF-ablation or telemedicine/mobile-health applications); experimental animal studies; epidemiological studies on cancer and exposure to extremely low frequency (ELF) fields, or on RF-EMF exposure and non-neoplastic diseases. Ineligible publication types (review/*meta*-analysis/editorial/comment) will also be screened at this stage and classified as irrelevant (0) or potentially relevant (9), depending on the topic.

Full-text articles will be retrieved for all records classified as certainly or possibly relevant (codes 1, 8 and 9), and examined to substantiate or modify the classification by relevance. All records with confirmed codes 1, 8, or 9 will be first classified by publication type. Eligible article types (original studies and related correspondence) will be further categorized by study design, setting/source of exposure to RF-EMF (wireless phone use; environmental sources; occupational sources), and investigated neoplasm(s). Eligibility for inclusion will then be assessed based on compliance with the predefined inclusion/exclusion criteria (§ 3.1). At completion of this stage, all identified articles will be divided into four non-overlapping groups: (i) irrelevant; (ii) relevant but ineligible for inclusion, with reason(s) for exclusion specified (recording main, if more than one applies); (iii) relevant and eligible for inclusion in one of the three systematic reviews (or in more than one, if multiple types of RF-EMF exposure are investigated); (iv) relevant as complementary evidence.

The list of studies excluded at full text (group ii) will be provided in the completed review paper.

#### 3.3.2 Selection of eligible studies

For papers addressing risk of multiple tumours and/or multiple exposure types, we will consider the analyses related to each specific exposure-neoplasm pair as separate studies.

##### 3.3.2.1 Multiple publications per study

Multiple publications with overlapping data from the same study will be identified by examining study acronym, author affiliations, study design, enrolment criteria, and enrolment dates. We will include all articles on the study providing information relevant for each neoplasm and exposure contrast prioritized for our systematic reviews, select one to use as the primary record for data extraction and risk of bias assessment, and consider the others as secondary publications with annotation as being related to the primary record. We will consider as primary records the latest published follow-up/update for cohort and nested case-control studies, and the earliest paper for case-control studies. We emphasize that more than one paper per study can qualify for the role of primary record, depending on availability of information relevant for the various exposure-outcome pairs of interest. In the presumably rare cases where exactly the same set of data from an original study is reported in multiple papers (duplicate data), we will keep the first publication and exclude subsequent articles.

##### 3.3.2.2 Pooled analyses of primary studies

Pooled analyses of individual data from relevant primary studies (not to be confused with *meta*-analyses, which use published risk estimates as input data) are eligible for inclusion in our review. If a quantitative synthesis of results is feasible, we will avoid combining results from primary studies and pooled analyses with overlapping populations. That is, we will create more than one dataset per neoplasm (e.g., one including primary studies only and other(s) made of pooled analyses plus all other non-overlapping primary studies). The main neoplasm-specific *meta*-analyses will be based on one of these datasets (see § 3.7.1), while the others will be used in sensitivity analyses aimed at evaluating the robustness of the findings to changes in the dataset composition (see § 3.7.2).

#### 3.3.3 Disagreement between reviewers

Possible disagreements between reviewers involved in article and study selection (including decisions on between-study overlap) will be resolved by discussion; if no consensus can be reached, a final decision will be made by the two reviewers in charge of the study selection for each line of evidence.

#### 3.3.4 Reporting of information flow

We will document the selection process in a study flow diagram according to the PRISMA reporting guidelines (Page et al., 2021b).

### 3.4 Data extraction

For each included study, a standard set of details will be extracted from the relevant publications (Table 5). The study design is reported in brackets when data refer to either cohort or case-control studies (including variants thereof); lack of specification means relevance for both main study designs.

**Table 5:** Data extraction elements.

Topic	Items
Article	First author and publication year, full reference

Study	Study design: cohort; nested case-control study; population-based case-control study hospital-based case-control study; other design variants (specify)
	Study acronym (if any)
Subjects	Study population (description)
	Geography (country, region, state, etc.)
	Dates of study and sampling time frame (period of case ascertainment)
	Demographics (sex; age or lifestage at exposure and at outcome assessment)
	Number of subjects (target, enrolled, number per group in analysis)
	Person-years of observations, length of follow-up and follow-up rates per exposure group [cohort]
	Participation rates of cases and controls (possibly for exposed and unexposed separately, in each series) [case-control]
Methods	Inclusion/exclusion criteria and recruitment strategy
	Case ascertainment: cancer register; hospital-based; other source (specify)
	Case type: incident cases; cases alive at enrolment; deceased cases
	Reference group description [cohort]
	Control type: population based (source and sampling method); hospital based (type of diagnoses); other types (specify) [case-control]
	Proportion of proxies interviewed among cases and controls [case-control]
	Outcome type(s): one or more of the following: glioma, brain tumours (when only topography available), paediatric brain tumours (age 0–19 years), meningioma, acoustic neuroma, pituitary tumour, salivary gland tumours; childhood leukaemias (age 0–14 years); adult leukaemias; other type (specify)
	Outcome assessment: diagnostic methods (histology-based, %; imaging-based, %; cause of death only; not given)
	Exposure assessment timing: prospective *vs* retrospective (i.e., before *vs* after outcome occurrence, diagnosis or ascertainment)
	Exposure assessment methods (self-administered questionnaire, personal interview; computer assisted personal interview, network-operator customer lists; measurements, modelling, geocoded distance to a broadcast transmitter; JEM, SEM; occupational sector, job title, task)
	Exposure variables used in the analyses (e.g., ever *vs* never exposed; length of exposure; time since first exposure; exposure frequency; exposure level; cumulative exposure; others – specifying the variable unit and type: dichotomous/categorical/continuous)
	Statistical methods (specify)
Results	Mean/median exposure value within each exposure interval (for all relevant metrics)
	Number of cases and persons-years or total number of subjects per exposure level, including unexposed [cohort];
	Number of cases and controls per exposure level, including unexposed [case-control];
	Type of relative risk estimate (OR, HR, IRR, SMR)
	Measures of effect and confidence limits (for each prioritized exposure contrast
	Confounders or modifying factors and how they were considered in analysis (i.e., list of factors included in final model, or considered for inclusion but found to have little or no impact on the measures of effect and therefore not included in the final model)
Funding	Funding source

For all prioritized exposure contrasts, we will extract from each neoplasm-specific study the most (appropriately) adjusted measure of effect and 95% confidence limits per exposure category.

From the entire dataset of included studies, three subsets of equivalent size will be assigned to three reviewers (DB, KK, MSP) who will extract and record the relevant data. Three other reviewers (MB, MR, SL) will check the extracted information for completeness and accuracy as a quality control measure. The structure of the datasets that will be created is described in **Annex** **3**. Information inferred, converted, or estimated during data extraction will be marked by brackets, and corrections made after quality control will be annotated with a rationale.

#### 3.4.1 Dealing with missing data

We will request missing data considered important for the review (e.g., data to conduct a *meta*-analysis) from the corresponding author by email or phone, using the contact details available from the study report. We envisage two attempts of contact, two weeks apart. In case of no response within one month of the second, we will consider the attempt unsuccessful.

The scientific literature relevant to the planned systematic review spans four decades. Recency of publication is likely to be a strong determinant of both the quality of reporting, and the possibility to get unpublished information. For early studies, we expect the chance of obtaining missing data to be low for substantive reasons, regardless of the number of contact attempts.

### 3.5 Risk of bias assessment

#### 3.5.1 Risk of bias in studies

To assess the study’s internal validity, or risk of bias (RoB), we will follow the method developed by the National Toxicology Program - Office of Health Assessment and Translation (NTP-OHAT, 2019).

The OHAT approach was chosen because of its versatility. Consisting of a cohesive framework applicable to different evidence streams, study designs and topics, it allowed a harmonization of RoB assessment procedures across all systematic reviews commissioned by the WHO.

We developed a tailored version of the RoB tool (NTP-OHAT 2015), focussing on the bias questions applicable to the study designs eligible for inclusion in our reviews. These include: confounding; selection bias; attrition/exclusion/missing data bias; confidence in the exposure characterization; confidence in the outcome assessment; selective reporting; and appropriateness of statistical methods. In the sections addressing selection and outcome-information biases, the RoB tool developed by the Office of the Report on Carcinogens (NTP-ORoC, 2015) was also referred to.

Detailed information on the customization process, along with the tailored bias rating instructions and answer option forms, are provided in the annexed RoB protocol (**Annex 4**).

We will perform the RoB assessment at the exposure-outcome level, as studies eligible for inclusion in the current review may report on different neoplasms and multiple types/sources/settings of exposure to RF-EMF. This is in line with the Cochrane approach (Higgins et al., 2021b, Sterne et al., 2021), COSTER recommendation 5.2 (Whaley et al. 2020), and other guidance on conducting systematic reviews of observational studies of aetiology and risks from environmental or occupational exposures (Arroyave et al., 2021, Dekkers et al., 2019, Radke et al., 2019).

Depending on the number of included studies, up to six team members (DB, MB, KK, ME, MR, TL) will be involved in the RoB assessment, and one will coordinate the process (SL). The potential for bias of each neoplasm-specific study and related exposure-outcome contrasts will be rated in duplicate by two assessors. No assessor will evaluate studies that they co-authored. The subsets of studies assigned to each assessor pair will reflect the composition of the entire dataset of included studies in term of exposure type and study design. Rating conflicts will be resolved by consensus or arbitration by a third person (SL). First and modified ratings will be recorded securely, and we will study consistency and reason for variation.

We will also undertake a small-scale validation study of our customized RoB tool, as applied to studies on mobile phone use and brain tumour risk, to assess the agreement between the study classifications by potential for upward/downward bias and by plausibility of the measures of effect size (§ 3.1.5.3).

All assessors will be trained in a pilot-study undertaken right after completion of the study selection, rather than at the protocol stage as suggested by COSTER recommendation 1.4.7 (Whaley et al. 2020), to be able to select a sample of studies representative of the review datasets. However, during protocol development, all assessors participated in a pre-pilot aimed at testing and thereby improving the comprehensibility and ease of application of a preliminary version of the tailored RoB tool (see **Annex** **4, § I.6** for details).

The RoB assessment process will be managed using the Health Assessment Workplace Collaborative (HAWC) platform (Shapiro et al. 2018).

Major revisions of the RoB protocol occurring after the pilot-study or later during the review, will be documented with justification in the paper reporting on the completed review.

#### 3.5.2 Summary assessments of risks of bias

We will apply the OHAT’s 3-level tiering of the quality of individual studies, based on summary assessments of risk of bias for the domains most relevant to the specific systematic review (NTP-OHAT, 2019). This tiering differs from scaling, and is consistent with the Cochrane’s overall risk-of-bias judgement (Higgins et al., 2021b; Sterne et al. 2021). We will focus on selection/attrition biases, and exposure/outcome information biases*.* Tier-1 will comprise studies with definitely or probably low risk of bias for all key-items and most of other items; tier-3 will include studies with definitely or probably high risk of bias for all key-items and most of other items; and studies non-compliant with the above criteria will be classified as tier-2. We will use this ranking to assess the overall potential for bias in the body of evidence at the stage of quality of evidence assessment (see **Annex** **6**).

We will also consider using the tiering results in data synthesis (see § 3.7), although the possibility to perform meaningful subgroup analyses by bias-tiers will depend on the variability of proneness to influential biases in the dataset, and on the possibility to isolate the impact of one bias from those of competing biases (Savitz et al. 2019).

#### 3.5.3 Assessment of reporting bias

Reporting bias [or “*meta*-bias” (Shamseer et al. 2015)], comprises several kinds of distortions due to missing data in a synthesis (Page et al., 2021a, Sedgwick, 2015). We will minimize language bias by including studies in any language. We will address possible bias in the identification of occupational studies eligible for inclusion in SR-C by performing additional literature searches (see § 3.1.2.3 and **Annex 2**, **§ 4–5**). We will use both funnel plots, and the Egger’s test to examine funnel plot asymmetry. We note that interpretation of funnel plot and Egger’s test is challenging, as it is difficult to identify whether an association between study size and reported exposure/treatment effect is due to true heterogeneity, biases in individual studies, selective reporting, publication bias, or a combination of these (Hartwig et al., 2020, Sterne et al., 2011). Treatment of multiple publications of the same study is a neglected quality item of systematic reviews (Hennessy and Johnson 2020). Multiple publication bias occurs because of the increasing likelihood of a study being identified and included in a *meta*-analysis if its results are published more than once. When studies with shared populations are included in a *meta*-analysis, multiple counting of the same individual data will result in biased *meta*-risk estimates (“study aggregation” bias). Our predefined inclusion strategy (see § 3.3.2.1) and analysis plan (see § 3.7.2) are aimed at maximizing the size of the available dataset while avoiding multiple publication and study aggregation biases.

### 3.6 Synthesis of results

We will summarize the main features of all included studies in tables grouped and ordered by exposure type/setting/source (SR-A, SR-B, and SR-C), neoplasm, and study design.

Templates of the key study characteristic tables for cohort and case-control studies are provided in **Annex** **5,** Table 1, Table 2. The results of included studies will be outlined in summary of finding tables (**Annex 5,** Table 3), visual displays, and a narrative synthesis.

The outcome, the exposure, and age at diagnosis are the most relevant factors affecting comparability between studies eligible for inclusion in our review. We will not combine studies of different tumour types (ICD-O-3 main site or histology groups), neoplasm-specific risks from different exposure types and metrics, or risk of a specific tumour in relation to a given exposure type/metric in adults and children/adolescents (0–19 years).

For homogenous datasets (in terms of outcome, subjects’ lifestage, and exposure type/metric), we will not set a minimum size requirement for amenability to a *meta*-analysis. However, to address concerns about the large uncertainty in heterogeneity statistics from *meta*-analyses based on few studies (Fu et al., 2008, Ioannidis et al., 2007), we will calculate and report the confidence intervals of the I^2^ statistics. We will also preliminarily assess the heterogeneity in findings across studies (in terms of direction and magnitude of effects), to decide whether averaging individual measures of effect would produce meaningful results. Possible causes of inconsistency (e.g., design features and potential for selected types of bias) will be explored through stratified *meta*-analysis and *meta*-regression. In the presence of substantial unexplained heterogeneity, reporting of overall *meta*-risk estimates will be considered inappropriate, and confidence in the body of evidence will be reduced (see **Annex** **6**).

The synthesis of findings from the study subsets not meeting the requirements for inclusion in a *meta*-analysis will be based on a structured tabulation of results and visual displays, such as the effect direction plot in **Annex 5, Figure 1** (Anzures-Cabrera and Higgins, 2010, McKenzie and Brennan, 2021).

We present below the analysis plan of a *meta*-analysis of studies included in SR-A. A similar approach would be followed if a quantitative synthesis of data from other lines of evidence (SR-B, SR-C) is considered feasible.

### 3.7 Meta-analysis of studies on wireless phone use and risk of tumours in the head region

The aims of the *meta*-analysis will be to assess the strength of association and the shape of the exposure–response relationship; to quantify the degree of heterogeneity across studies; and to explore the source of inconsistency, if any (Dekkers et al., 2019, Greenland and Rourke, 2012, Savitz and Wellenius, 2016).

#### 3.7.1 Main analyses

The *meta*-analyses will be neoplasm- and exposure-specific, performed separately for glioma, meningioma, acoustic neuroma, pituitary tumours, and salivary gland tumours, in relation to usage of each type of wireless phone (mobile or cordless). Should the systematic review result in other tumours of the head region suitable for a *meta*-analysis, we will augment the neoplasm series.

We will use the natural logarithms of the most (appropriately) adjusted point estimates of relative risk (RR, HR, OR), and related 95% CLs, extracted from the relevant publications as input for the *meta*-analyses, focussing on the exposure metrics and contrasts below.

a. For the binary exposure variable “ever *vs* never” use, we will perform fixed- and random-effects overall and stratified *meta*-analyses, using the I^2^ statistic (Higgins et al. 2003) to assess the statistical heterogeneity in results across studies, and between cohort and case-control studies.
b. For the categorical variable “time since start of use”, the across-study variability in cutpoints will be dealt with by aligning (to the possible extent) the original categories to a “standard” classification into short-term (<5 years), mid-term (5–9 years), and long-term use (≥10 years). When needed, we will combine the original measures of effect for adjacent categories using the inverse variance weighting method (fixed effects model). We will first perform *meta*-analyses stratified on study design for each category of time since start use (short-term, mid-term-term, and long-term) *vs* no exposure. Then, we will carry out analyses of risk by increasing categories of time since start use by mixed effects *meta*-regression (Harbord and Higgins, 2008). To this purpose, the three levels of this categorical variable will be assigned increasing numerical values (short-term = 1; mid-term = 2; long-term = 3), in order to approximate an analysis of trend by latency. The aim of the *meta*-regression will be to assess the amount of overall heterogeneity in the exposure–response function attributable to differences between study groups classified by design (cohort *vs* case-control), exposure assessment method, and (if feasible) gradient of susceptibility to upward and towards null biases. In these analyses, the between-groups variance (t^2^) is estimated by the restricted maximum likelihood (REML) method, and the proportion of between-studies variance explained by the covariate/s (adjusted R^2^) is calculated by comparing the estimated between-studies variance (t^2^) with its value when no covariates are fit (t_0_^2^) (Harbord and Higgins, 2008).
c. We will perform dose–response *meta*-analyses of neoplasm risks per cumulative call time and total number of calls. We will use weighted mixed effects models suitable for table of correlated estimates (Crippa et al., 2019, Orsini, 2021). A single exposure value is assigned to each category based on what has been reported (mean, median, midpoint) within each study. In case the typical exposure value within each exposure interval is not available from the publication, it will be assigned according to its distribution. We will use regression splines of different degrees to answer specific questions about the dose–response relationships (Orsini, 2021, Orsini and Spiegelman, 2020). The heterogeneity of dose–response gradients across studies is taken into account by using random-effects for the regression coefficients of the exposure transformations. The main target of statistical inference (test of hypothesis, confidence intervals) is the pointwise dose–response relationship for the average study. To examine the magnitude of heterogeneity across studies, the best linear unbiased predictions (BLUP) of the random effects will be used. A comparison of alternative candidate dose–response models will be done using the Akaike Information Criteria, balancing goodness of fit and overall number of parameters. Stratified analyses according to relevant design or scientific factors (e.g., gradient of susceptibility to systematic and differential exposure measurement errors) will be done using weighted mixed effects model.

#### 3.7.2 Sensitivity analyses

To assess changes over time in the summary measures of effect for the neoplasms most commonly investigated (glioma, meningioma, and acoustic neuroma) in long-term users, we will perform cumulative *meta*-analyses (Sterne 2016) on the dataset of studies ordered by accrual dates of cases and publication date. The results of these analyses will be reported in cumulative forest plots (Anzures-Cabrera and Higgins 2010), where each *meta*-RR is the pooled estimate of past studies and the more recent one.

We will also assess the sensitivity of results to variations in the dataset composition. As previously noted (§ 3.3.2.2), we will include primary studies and partially or completely overlapping pooled analyses of the former, but we will create multiple datasets per neoplasm to avoid multiple counting of the same individual data. We will perform our main analyses on one dataset per tumour (e.g., that including the largest overall number of exposed cases), and sensitivity analyses on all other datasets.

Additional sensitivity analyses will be carried out excluding studies:

• classified in the tier-3 category (see § 3.5.2);
• at high risk of upward biases (recall bias, and detection bias for acoustic neuroma);
• at high risk of bias towards null (random exposure misclassification in the non-null scenario);
• reporting implausible effect sizes (see § 3.1.5.3) for RR estimates above or below the null.

Findings from all sensitivity analyses will be displayed in summary forest plots (Anzures-Cabrera and Higgins 2010).

The analyses will be performed using the *meta*-analysis software developed in Stata (Palmer and Sterne 2016), the drmeta-Stata command (Orsini 2021), and the dosresmeta-R package (Crippa and Orsini, 2016).

### 3.8 Confidence in evidence assessment

We will assess confidence that the study findings accurately reflect the true exposure-effect association (conventionally referred to as certainty of the evidence or quality of the body of evidence), using the OHAT’s approach (NTP-OHAT, 2019; Rooney et al. 2014).

The WHO endorses the Grading of Recommendations Assessment, Development and Evaluation (GRADE) method to assess the quality of evidence in systematic reviews of the scientific literature (WHO, 2014a), acknowledging that its adaptation to questions regarding environmental exposures is under development (Morgan et al., 2019, Morgan et al., 2016).

The strength of GRADE rests on the use of a structured and transparent assessment framework, and on a standard lexicon to formulate conclusions and recommendations. It specifies four levels of the quality of a body of evidence for a given outcome: high, moderate, low and very low. In the current version for systematic reviews of health care interventions, randomized control trials (RCT) and non-randomized studies (NRS) are assigned initial ratings of high and low quality, respectively; the certainty of evidence can be lowered based on five domains (risk-of-bias, inconsistency, indirectness, imprecision, publication bias); the evidence provided by NRS can be upgraded for large magnitude of effect, dose–response gradient, and opposing residual bias and confounding (Schünemann et al. 2021).

The a-priori downgrading of observational studies is considered the most challenging feature of evidence appraisal methods adapted from clinical epidemiology, because the cross-sectional, case-control or cohort designs may be the only feasible or ethical option to provide evidence on environmental causes of diseases (Arroyave et al., 2021, Morgan et al., 2016, Steenland et al., 2020).

This problem has been addressed in different ways by currently available adaptations of GRADE to studies of aetiology and risk (Héroux and Verbeek, 2018, Johnson et al., 2016; NTP-OHAT 2019; Thayer and Schünemann, 2016, Woodruff and Sutton, 2014).

Approaches relying on the best available or achievable evidence as a reference standard [e.g., (WHO 2018)] do not appear suitable to assessments of multiple health hazards from several types of exposure to RF-EMF. Changes in the rating scale across evidence lines would lead to differing certainty in decisions based on the questions asked (Schünemann et al. 2019), compromising the internal coherence of the appraisal and impairing risk communication.

The confidence rating method developed by OHAT has features that mitigate the above concerns. It conforms to GRADE in terms of definition of quality of evidence, explicit consideration of the eight GRADE assessment criteria for observational studies, and terminology used to formulate the conclusions (Schünemann et al., 2013). Compared to the original GRADE system, the major change introduced by OHAT is a four-level initial confidence rating (high, moderate, low, and very low), based on the number of favourable features of the study design (controlled exposure, exposure prior to outcome, individual outcome data, presence of a comparison group), applied to every evidence stream (human, animal and cell studies). An additional difference is a fourth upgrading factor: consistency across study designs and populations for human studies, or across multiple species and models for animal studies. This allows to fully exploit findings from analyses of the sources of heterogeneity in results across studies, with the possibility to lessen confidence in the evidence in the presence of substantial unexplained inconsistency, and increase it for consistency not attributable to bias or other dissuading concerns (NTP, 2019, NTP, 2019). The methodological congruity between two interrelated step of the systematic review (the assessments of RoB at the individual study level and across studies) was a further reason behind the choice of the OHAT’s approach to confidence rating.

At the request of WHO, the OHAT’s extra updating domain will not be considered in our assessment.

Details about the process and related decision rules, including a template of the “Evidence Profile Table”, are provided in the evidence assessment protocol (**Annex** **6**).

### 3.9 Strength of evidence assessment

The current edition of the WHO handbook for guideline development (WHO, 2014a) does not envisage any structured framework to assess the strength of evidence within and across the various evidence streams considered in health hazard assessments. At the request of WHO, we will not assess the level of evidence for health effect or for no health effect [Step 6 of the OHAT approach (NTP-OHAT, 2019)].

Our overall conclusions will be primarily based on the line of evidence with the highest confidence when considered across the multiple exposure-neoplasm pairs examined in the systematic review, taking into account the internal coherence and the external plausibility of the original study findings.

Four team members (MB, ME, MR, SL) will prepare a preliminary version of the confidence ratings and overall conclusions, submit it for revision to the other team members, and finalize the collectively agreed assessment.

## Financial support

This project is supported by the World Health Organization (grant numbers: RAD 2020/1031788–0; RAD 2020/994756–0). Co-financing was provided by the New Zealand Ministry of Health; the Istituto Superiore di Sanità in its capacity as a WHO Collaborating Centre for Radiation and Health; ARPANSA as a WHO Collaborating Centre for Radiation Protection.

## Role of funders

A strict oversight was exercised by the WHO Secretariat to ensure that all commissioned systematic reviews were planned according to a harmonized and good practice standard. The other sponsors had no role in developing the protocol.

## Conflicts of interest

Susanna Lagorio was principal investigator (April 2019 – March 2020) of the research project “BRiC 2018/06 - Systematic reviews of exposure to radiofrequency fields and cancer”, supported by the Italian Workers’ Compensation Authority, a public no-profit entity (grant code I85B19000120005). Her employment duties involve provision of advice on health hazards from exposure to RF-EMF to the Italian Ministry of Health and Higher Health Council.

Maria Feychting has a permanent position as Professor of Epidemiology at Karolinska Institutet, Stockholm, Sweden since 2005. She has served as advisor to a number of national and international public advisory and research steering groups concerning the potential health effects of exposure to non-ionizing radiation, including the WHO (ongoing), Public Health England Advisory Group on Non-ionising Radiation - AGNIR (2009–2017), the Norwegian Public Health Institute (2010–2012), the Swedish Council for Working Life and Social Research (2003–2012), the Swedish Radiation Safety Authority’s independent scientific expert group on electromagnetic fields (2003–2011). She was member of the International Commission on Non-Ionizing Radiation Protection (ICNIRP), an independent body setting guidelines for non-ionizing radiation protection (2008-May 2020), and vice chairman of the Commission (May 2016-May 2020).

Ken Karipidis as part of his employment is involved in the provision of advice to the Australian Commonwealth Government, Australian States and Territories and the general public on the risks and health effects of exposure to ionising and non-ionising radiation. He is also a member of the International Commission on Non-Ionizing Radiation Protection where he contributes in the development and dissemination of science-based advice on limiting exposure to non-ionizing radiation.

Martin Röösli’s research is entirely funded by public entities or not for profit foundations. He has served as advisor on potential health effects of exposure to non-ionizing radiation to several national and international public advisory and research steering groups, including the World Health Organization, the International Agency for Research on Cancer, the International Commission on Non-Ionizing Radiation Protection, the Swiss Government (member of the working group “mobile phone and radiation” and chair of the expert group BERENIS), the German Radiation Protection Commission (member of the committee Non-ionizing Radiation (A6) and member of the working group 5G (A630)) and the Independent Expert Group of the Swedish Radiation Safety Authority. From 2011 to 2018, M.R. was an unpaid member of the foundation board of the Swiss Research Foundation for Electricity and Mobile Communication, a non-profit research foundation at ETH Zurich. Neither industry nor nongovernmental organizations are represented on the scientific board of the foundation.

Mark Elwood has given expert advice on topics in electromagnetic fields and health, and on the objective interpretation of epidemiological and other scientific information, over many years to individuals and groups, including government ministries, environmental regulators, community groups, commercial organisations, and formal inquiries by government and professional groups including parliamentary and legal proceedings. Some of this work has been financially supported, by universities, health care organisations, research bodies, or by government, professional or commercial groups. Some work has been reported ‘blind’, with the client being unidentified.

The other authors declare that they have no known conflicts of interest.

## Guarantors of the review

Susanna Lagorio and Maria Blettner (co-principal investigators).

## CRediT authorship contribution statement

**Susanna Lagorio:** Conceptualization, Data curation, Formal analysis, Funding acquisition, Investigation, Methodology, Writing - original draft. **Maria Blettner:** Conceptualization, Funding acquisition, Investigation, Methodology, Project administration, Writing - review & editing. **Dan Baaken:** Data curation, Formal analysis, Investigation, Project administration, Writing - review & editing. **Maria Feychting:** Conceptualization, Methodology, Writing - review & editing. **Ken Karipidis:** Funding acquisition, Investigation, Methodology, Writing - review & editing. **Tom Loney:** Investigation, Methodology, Writing - review & editing. **Nicola Orsini:** Data curation, Formal analysis, Investigation, Methodology, Writing - review & editing. **Martin Röösli:** Investigation, Methodology, Writing - review & editing. **Marilia Silva Paulo:** Data curation, Investigation, Writing - review & editing. **Mark Elwood:** Conceptualization, Funding acquisition, Investigation, Methodology, Writing - review & editing.

## Declaration of Competing Interest

The authors declare that they have no known competing financial interests or personal relationships that could have appeared to influence the work reported in this paper.
