# Supplementary material for: The effect of exposure to radiofrequency fields on cancer risk in the general and working population: A protocol for a systematic review of human observational studies
Source: Environ Int. 2021 Dec;157:106828. doi: 10.1016/j.envint.2021.106828 (PMC8484862; doi:10.1016/j.envint.2021.106828)
Supplement: Supplementary data 1 [file mmc1.docx]

**L3-PRISMA Report for SR Protocols to EI, version 1.0, 15 March 2017**

**Title of submitted paper**: The effect of exposure to radiofrequency fields on cancer risk in the general and working population: a protocol for a systematic review of human observational studies

**Corresponding author:** Susanna Lagorio

| **#** | **Item** | **Guidance** | **On page #** | **Author Comments** |
| --- | --- | --- | --- | --- |
| **Title** | | | | |
| 1 | Identification | Identify the report as a systematic review, or systematic review and meta-analysis, as appropriate. | 1 | Identified as a systematic review. A meta-analysis will likely be feasible on a subset of data (see § 3.7) |
| 2 | Update | If the protocol is for an update of a previous systematic review, identify as such. | - | Not applicable |
| **Registration** | | | | |
| 3 | Registration | If registered, provide the name of the registry (e.g., PROSPERO) and registration number. | 2 | - |
| **Authors** | | | | |
| 4 | Contact | Provide name, institutional affiliation, and e-mail address of all protocol authors; provide physical mailing address of corresponding author. | 1 | - |
| 5 | Contributions | Describe contributions of protocol authors and identify the guarantor of the review. | 34 | - |
| **Amendments** | | | | |
| 6 | Amendments | If the protocol represents an amendment of a previously completed or published protocol, identify as such and list changes; otherwise, state plan for documenting important protocol amendments. | - | Not applicable |
| **Support** | | | | |
| 7 | Sources | Indicate sources of financial or other support for the review. | 2, 33 | - |
| 8 | Sponsor | Provide name for the review funder/s and/or sponsor/s. | 2, 33 | - |
| 9 | Roles | Describe roles of funder(s), sponsor(s), and/or institution(s), if any, in developing the protocol. | 4 (§1.1), 33 | - |
| **Introduction** | | | | |
| 10 | Rationale | Describe the rationale for the review in the context of what is already known | 6-7 (§1.4) | - |
| 11 | Objectives | Provide an explicit statement of the question(s) the review will address, with specific reference to:   - Participants - Interventions / Exposures (as appropriate) - Comparisons - Outcomes - Study design | 7-8 (§2) | PECO statements in Table 2. |
| **Methods** | | | | |
| 12 | Eligibility criteria | Specify the study characteristics (e.g., PICO/PECO, study design, setting, time frame) and report characteristics (e.g., years considered, language, publication status) to be used as criteria for eligibility for the review. | 9-21 (§3.1) | - |
| 13 | Information sources | Describe all intended information sources (e.g., electronic databases, contact with study authors, trial registers, or other grey literature sources) with planned dates of coverage. | 21-22 (§3.2) | - |
| 14 | Search strategy | Present draft of search strategy to be used for at least one electronic database, including planned limits, such that it could be repeated. | Annex2 | Annex2 also include a performance assessment of the draft Medline queries, intentionally designed to privilege sensitivity over precision (89% vs 9%). |
| 15 | Data management | Describe the mechanism(s) that will be used to manage records and data throughout the review. | 22-24 (§3.3);  24-25 (§3.4), and Annex3;  26-27 (§3.5), and Annex4;  28-31 (§3.6-3.7), and Annex5;  31-32 (§3.8-3.9), and Annex6. | §3.3 Study selection;  §3.4 Data extraction;  §3.5 Risk-of-bias;  §3.6 Data synthesis;  §3.7 Meta-analyses;  §3.8 Confidence in evidence  § 3.9 Strength of evidence |
| 16 | Selection process | State the process that will be used for selecting studies (e.g., two independent reviewers) through each phase of the review (i.e., screening, eligibility, and inclusion in meta-analysis). | 22-24 (§3.3) | - |
| 17 | Data collection process | Describe planned method of extracting data from reports (e.g., piloting forms, done independently, in duplicate), any processes for obtaining and confirming data from investigators. | 24-25 (§3.4), and Annex3 | - |
| 18 | Data items | List and define all variables for which data will be sought (e.g., PICO items, funding sources), any pre-planned data assumptions and simplifications | 24-25, Table 5, and Annex3 | - |
| 19 | Outcomes and prioritisation | List and define all outcomes for which data will be sought, including prioritization of main and additional outcomes, with rationale. | 16-17, Table 4 | - |
| 20 | Bias in individual studies | Describe anticipated methods for assessing risk of bias of individual studies, including whether this will be done at the outcome or study level, or both; state how this information will be used in data synthesis | 26-27 (§3.5.1 & 3.5.2), and Annex4 | - |
| 21 | Data synthesis criteria | Describe criteria under which study data will be quantitatively synthesized | 28-29 (§3.6) | - |
| 22 | Summary measures | If data are appropriate for quantitative synthesis, describe planned summary measures, methods of handling data, and methods of combining data from studies, including any planned exploration of consistency (e.g., I^2^, Kendall’s tau). | 29-31 (§3.7) | §3.7.1. includes subgroup analyses, meta-regression, and dose-response meta-analyses.  §3.7.2 includes cumulative meta-analyses and sensitivity analyses. |
| 23 | Additional analyses | Describe any proposed additional analyses (e.g., sensitivity or subgroup analyses, meta-regression) | 30-31 (§3.7.1 and 3.7.2) |  |
| 24 | Alternative synthesis | If quantitative synthesis is not appropriate, describe the type of summary planned | 28-29 (§3.6), and Annex5 | - |
| 25 | Meta-bias | Specify any planned assessment of meta-bias(es) (e.g., publication bias across studies, selective reporting within studies) | 27-28 (§3.5.3)  21-22 (§3.2)  Annex4, §2.6 | Grey literature bias is discussed in § 3.2, p. 21.  Selective reporting within studies is part of the risk-of-bias assessment (Annex4). |
| 26 | Confidence in cumulative evidence | Describe how the strength of the body of evidence will be assessed (e.g., GRADE) | 31-32 (§3.8-3.9)  Annex6 | - |

*Environment International* modified PRISMA-P report adapted from: Moher D, Shamseer L, Clarke M, Ghersi D, Liberati A, Petticrew M, Shekelle P, Stewart LA. Preferred Reporting Items for Systematic Review and Meta-Analysis Protocols (PRISMA-P) 2015 statement. Syst Rev. 2015;4(1):1. (Changes are minor, with text edits to accommodate the subject matter of the journal and formatting to fit page.)
