# Supplementary material for: The effect of exposure to radiofrequency fields on cancer risk in the general and working population: A protocol for a systematic review of human observational studies
Source: Environ Int. 2021 Dec;157:106828. doi: 10.1016/j.envint.2021.106828 (PMC8484862; doi:10.1016/j.envint.2021.106828)
Supplement: Supplementary data 2 [file mmc2.docx]

Systematic review of human observational studies on the effect of exposure
to radiofrequency fields and cancer risk in the general and working population

**Literature search strategy**

**Table of content**

[1. Calibration and performance assessment of preliminary query versions 1](#_Toc74835286)

[2. Medline search strategy 6](#_Toc74835287)

[3. Embase search strategy 7](#_Toc74835288)

[4. Medline search strategy for cancer studies in selected occupational groups 8](#_Toc74835289)

[5. Embase search strategy for cancer studies in selected occupational groups 9](#_Toc74835290)

[6. References 10](#_Toc74835291)

1. **Calibration and performance assessment of preliminary query versions**

We conducted a calibration study of the search strategy for Medline through PubMed, following the “empirical validation approach” described by (Hausner et al. 2012), consisting of three steps: generation of a test set; validation of the search output against the test set, and standardized documentation of the search strategy.

We were provided by the WHO Secretariat with a draft PubMed query tailored for the SR-A (Exposure to RF-EMF from mobile phone use, and risks of tumours in the head region), reported below.

#1 ("cell phone"[MeSH Terms] OR "radiofrequency field"[Text Word] OR "radiofrequency electromagnetic field"[Text Word] OR "radiofrequency exposure"[Text Word] OR "mobile phone"[Text Word] OR "mobile telephone"[Text Word] OR "cell phone”[Text Word] OR "cellular phone"[Text Word] OR "cellular telephone"[Text Word] OR "radiofrequency fields"[Text Word] OR "radiofrequency electromagnetic fields"[Text Word] OR "mobile phones"[Text Word] OR "mobile telephones"[Text Word] OR "cellular phones"[Text Word] OR "cellular telephones"[Text Word] OR "radio transmitter"[Text Word] OR "radio transmitters"[Text Word] OR "base station"[Text Word] OR "base stations"[Text Word] OR "radar"[Text Word] OR "plastic sealers"[Text Word])

#2 ("etiology"[MeSH Subheading] OR "etiology"[Title/Abstract] OR "etiological"[Title/Abstract] OR "epidemiologic studies"[MeSH Terms] OR "comparative study"[Publication Type] OR "case reference"[Title/Abstract] OR "cross sectional"[Title/Abstract] OR "risk factors"[MeSH Terms] OR "case control study"[Title/Abstract] OR "cohort study"[Title/Abstract] OR "prospective study"[Title/Abstract]OR "retrospective study"[Title/Abstract] OR "confounding"[Title/Abstract] OR "confounders"[Title/Abstract] OR "questionnaire"[Title/Abstract])

#3 ("occupational exposure"[MeSH Terms] AND "radio waves"[MeSH Terms])

#4 ("epidemiologic studies"[MeSH Terms] AND "radio waves"[MeSH Terms] NOT "ablation"[Text Word])

#5 (“cancer”[Text Word] OR “tumour”[Text Word] OR “tumours”[Text Word] OR “tumor”[Text Word] OR “tumors”[Text Word] OR “glioma”[Text Word] OR “meningioma”[Text Word] OR “acoustic neuroma”[Text Word] OR “vestibular schwannoma”[Text Word] OR "Brain Neoplasms/epidemiology"[MeSH Terms] OR "Neoplasms"[MeSH Terms])

((#1 AND #2) OR #3 OR #4) AND #5

We revised this query including additional terms (TextWord or MeSH) relevant for the SR1-B (Exposure to RF-EMF from fixed-sire transmitters and risks of brain tumours and leukaemias), and SR1-C (Occupational exposure to RF-EMF and risks of brain cancer and leukaemias).

We went through five extensions of the original query, arriving at the 6^th^ version below.

((“Cell Phone Use/adverse effects”[Mesh] OR “Cell Phone Use/statistics and numerical data”[Mesh] OR “Cell Phone Use/trends”[Mesh] OR “Microwaves/adverse effects”[Mesh] OR "mobile phone"[Text Word] OR "mobile telephone"[Text Word] OR "cell phone"[Text Word] OR "cellular phone"[Text Word] OR "cellular telephone"[Text Word] OR "radiofrequency fields"[Text Word] OR "radiofrequency electromagnetic fields"[Text Word] OR "mobile phones"[Text Word] OR "mobile telephones"[Text Word] OR "cellular phones"[Text Word] OR "cellular telephones"[Text Word] OR "radiofrequency field"[Text Word] OR "radiofrequency electromagnetic field"[Text Word] OR "radiofrequency exposure"[Text Word] OR "radiofrequency radiation"[Text Word] OR "high-frequency electromagnetic fields"[Text Word] OR “microwave radiation"[Text Word] OR "radio transmitter"[Text Word] OR "radio transmitters"[Text Word] OR "broadcast transmitter"[Text Word] OR "broadcast transmitters"[Text Word] OR "broadcasting"[Text Word] OR “radio operators”[Text Word] OR “telegraph operators”[Text Word] OR “radar operators”[Text Word] OR "base station"[Text Word] OR "base stations"[Text Word] OR "radar"[Text Word] OR "plastic sealers"[Text Word]) AND ("etiology"[MeSH Subheading] OR "etiology"[Title/Abstract] OR "etiological"[Title/Abstract] OR "epidemiologic studies"[MeSH Terms] OR "comparative study"[Publication Type] OR "case reference"[Title/Abstract] OR "cross sectional"[Title/Abstract] OR "risk factors"[MeSH Terms] OR "case control study"[Title/Abstract] OR "cohort study"[Title/Abstract] OR "case control"[Title/Abstract] OR "case-control"[Title/Abstract] OR "cohort"[Title/Abstract] OR "prospective study"[Title/Abstract] OR "retrospective study"[Title/Abstract] OR "confounding"[Title/Abstract] OR "confounders"[Title/Abstract] OR "questionnaire"[Title/Abstract] OR “Cohort Studies”[MeSH Terms] OR “Case-Control Studies”[MeSH Terms] OR “Surveys and Questionnaires”[MeSH Terms] OR "Retrospective Studies"[Mesh] OR "Prospective Studies"[Mesh]) OR ("occupational exposure"[MeSH Terms] AND "radio waves"[MeSH Terms]) OR ("occupational exposure"[MeSH Terms] AND "microwaves"[MeSH Terms]) OR ("occupational exposure"[Text Word] AND "radio waves"[Text Word]) OR ("occupational exposure"[Text Word] AND "microwave radiation"[Text Word]) OR ("occupational exposure"[Text Word] AND "microwaves"[Text Word]) OR ("occupational exposure"[Text Word] AND "radio waves"[Text Word]) OR ("occupational exposure"[Text Word] AND "non-ionising radiation"[Text Word]) OR ("occupational exposure"[Text Word] AND "non-ionizing radiation"[Text Word]) OR ("occupational exposure"[Text Word] AND "electromagnetic fields"[Text Word]) OR ("residential exposure"[Text Word] AND "electromagnetic fields"[Text Word] OR ("epidemiologic studies "[MeSH Terms] AND "radio waves"[MeSH Terms] NOT "ablation"[Text Word] NOT "radiofrequency ablation"[MeSH Major Topic] NOT "therapy"[MeSH Subheading]) OR ("epidemiologic studies "[MeSH Terms] AND "microwaves"[MeSH Terms] NOT "ablation"[Text Word] NOT "radiofrequency ablation"[MeSH Major Topic] NOT "therapy"[MeSH Subheading]) OR ("epidemiologic studies "[MeSH Terms] AND "microwaves/adverse effects"[Mesh] NOT "ablation"[Text Word] NOT "radiofrequency ablation"[MeSH Major Topic] NOT "therapy"[MeSH Subheading])) AND (“cancer”[Text Word] OR “tumour”[Text Word] OR “tumours”[Text Word] OR “tumor”[Text Word] OR “tumors”[Text Word] OR “glioma”[Text Word] OR “meningioma”[Text Word] OR “acoustic neuroma”[Text Word] OR “vestibular schwannoma”[Text Word] OR "Brain Neoplasms/epidemiology"[MeSH Terms] OR “leukemia”[Text Word] OR “lymphoma”[Text Word] OR “non-Hodgkin lymphoma”[Text Word] OR “non-Hodgkin’s lymphoma”[Text Word] OR “melanoma”[Text Word] OR “testicular cancer”[Text Word] OR “breast cancer”[Text Word] OR “cancer mortality”[Text Word] OR “cancer risks”[Text Word] OR “cancer risk”[Text Word] OR “cause-specific mortality” OR “cancer mortality”[Text Word] OR “Neoplasms/analysis”[MeSH Terms] OR “Neoplasms/epidemiology”[MeSH Terms] OR “Neoplasms/etiology”[MeSH Terms] OR “Neoplasms/mortality”[MeSH Terms] OR “Neoplasms/prevention and control”[MeSH Terms] OR “Neoplasms/statistics and numerical data”[MeSH Terms])

As the test set (gold standard), we used a library of seed studies created from the reference lists of 19 comprehensive reviews of the epidemiological literature on RF-EMF and cancer risk, including the IARC Monograph on RF-EMF and subsequent expert panel reports published up to February 2020 (AGNIR 2012; ANSES 2013; 2016; ARPANSA 2014; CCARS 2017; Demers et al. 2014; FDA 2020; HCN 2016; IARC 2013; ICHENF 2018; ICNIRP 2020; SCENIHR 2015; SSM 2013; 2014; 2015; 2016; 2018; 2019; WHO 2014). To extend the time coverage of these reviews (1978-early 2018), we recorded 13 additional records identified through a bibliographic search on EMF-Portal with time limits January 2018-July 2019.

The library consists of 417 unique records, all of which are classified by exposure type and other study features, so that it is easy to create subsets using the EndNote “smart group” function (Table 1).

The subset of **150** articles qualifying for inclusion in any of our three systematic reviews (SR-A, SR-B, or SR-C) is highlighted in blue cells in Table 1.

Note that the sum of papers relevant for each systematic review (SR-A=**105**+SR-B=**12**+SR-C=**35** = 152) does not coincides with the overall total due to two paper eligible for inclusion in more than one review.

We first validated the original query (#1), and iteratively checked titles, key-words and index terms of unidentified relevant papers, including new relevant terms (as TextWord, or MeSH terms/subheadings) in the revised versions (#2 to #6). The six queries were then re-launched on the same day (2020/04/21), to avoid differences across outputs due to changes in records indexed in PubMed.

The record-linkage between the search results and the seed study library was performed as described below. We created a structured EndNote library for each query. We run the searches and exported the output to citation manager. The resulting NBIB formatted files were imported in each query-specific library. All references retrieved were stored in a folder called “Q_i_”. Then, the 150 relevant records from the seed studies library were imported in each query-specific library, with the duplicate option “Discard duplicates”. The imported records, consisting of relevant papers not identified by the search, were stored in a separate folder “Imported seed studies”. Last, based on the values of the variables study-type and exposure-type, smart groups were created to distinguish the unidentified papers relevant for each systematic review (SR-A, SR-B, SR-C).

To assess and compare the query performance, we calculated the indicators “sensitivity” (number of relevant records identified / total number of “gold standard” papers), and “precision” [1-(number of irrelevant records / total number of retrieved records)] (Hausner et al. 2012).

The results of the performance assessment are reported in Table 2. The overall sensitivity varied between 0.78 and 0.89 across queries. For all queries, the PECO-specific sensitivity was always higher for papers relevant to SR-A, than for articles relevant to SR-B and SR-C. All queries have low overall precision, ranging between 0.09 and 0.13. The query #6 (no filters), with the highest sensitivity for all SRs in spite of the poorest overall precision, was chosen as the preferred search strategy and the finalized searches for Medline and Embase (§ 2 and § 3 below) were developed from this query.

**Table 1. Library of epidemiologic seed studies (**417 records)**: paper distribution by evidence stream, exposure type, study type, study design, and article content** (eligible articles in blue cells)

| **Exposure** | | | **Study** | | **Article** | **N°** | |
| --- | --- | --- | --- | --- | --- | --- | --- |
| **Setting** | **Type** | **Sources** | **Type** | **Design** |  |  |  |
|  | Near-field exposure sources | Wireless phones | Analytical | Cohort | Full study report | 8 |  |
|  |  |  |  | Case-Control |  | 78 |  |
|  |  |  |  | Case-Case |  | 6 |  |
|  |  |  |  | Survival |  | 3 |  |
|  |  |  |  |  | Methods | 5 |  |
|  |  |  |  |  | Correspondence | 8 |  |
|  |  |  |  |  | | | *106* |
|  |  |  | Descriptive | Case-Report | Full study report | 2 |  |
|  |  |  |  | Spatial correlation \| Cluster |  | 4 |  |
|  |  |  |  | TimeTrend and Simulation |  | 60 |  |
|  |  |  |  | Exposure assessment \|Dosimetry |  | 14 |  |
|  |  |  |  |  | Correspondence | 9 |  |
|  |  |  |  |  | | | *89* |
|  |  |  | Bias Analysis | Exposure validation |  | 10 |  |
|  |  |  |  | Selection bias assessment |  | 3 |  |
|  |  |  |  | Multiple bias modelling |  | 2 |  |
|  |  |  |  |  | | | *15* |
|  |  |  | **Subtotal** | | | | **212** |
|  | Far-field exposure sources | Radio-TV \| Base Station | Analytical | Cohort |  | 1 |  |
|  |  |  |  | Case-Control |  | 7 |  |
|  |  | MW (2.5-4 GHz) |  | Cohort |  | 2 |  |
|  |  | DECT Base Station |  | Case-Control |  | 1 |  |
|  |  |  |  |  | Correspondence | 1 |  |
|  |  |  |  | | | | *12* |
|  |  | Radio-TV \| Base Station | Descriptive | Spatial correlation \| Cluster |  | 29 |  |
|  |  | Radio-TV \| Base Station |  | Exposure assessment \|Dosimetry |  | 10 |  |
|  |  | Wi-Fi |  |  |  | 8 |  |
|  |  | Smart meters |  |  |  | 4 |  |
|  |  | RFID |  |  |  | 1 |  |
|  |  | Multiple sources |  |  |  | 5 |  |
|  |  |  |  |  | Correspondence | 1 |  |
|  |  |  |  | | | | *58* |
|  |  | Radio-TV \| Base Station | Bias Analysis | Exposure validation |  | *2* | |
|  |  |  | **Subtotal** | | | | **72** |
| Workplace | Near- or Far-field | Occupational (incl. 2 cohort studies of amateur radio operators) | Analytical | Cohort |  | 19 |  |
|  |  |  |  | Case-Control |  | 15 |  |
|  |  |  |  | Nested Case-Control |  | 1 |  |
|  |  |  |  |  | | | *35* |
|  |  |  | Descriptive | Case Report |  | 2 |  |
|  |  |  |  | Exposure assessment \|Dosimetry |  | 9 |  |
|  |  |  |  |  | | | *11* |
|  |  | **Subtotal** | | | | | **46** |
| Medical | | Cochlear Implant | Analytical | Cohort |  | 1 |  |
| Not applicable | | | Synthesis | Reviews |  | 58 |  |
|  |  |  |  | Meta-analyses |  | 16 |  |
|  |  |  |  | Others |  | 5 |  |
|  |  |  | Miscellaneous topic and design | |  | 7 |  |
|  |  |  | **Subtotal** | | | | **87** |
| **TOTAL (unique)** | | | | | | | **417** |

**Table 2. Results of the query performance assessment**

| **ID** | **Note** | **Records** | **Relevant Identified** | | | | **Sensitivity** | | | | **Precision** |
| --- | --- | --- | --- | --- | --- | --- | --- | --- | --- | --- | --- |
|  |  |  | **SR-A** | **SR-B** | **SR-C** | **Overall** | **SR-A** | **SR-B** | **SR-C** | **Overall** | **Overall** |
| 1 | WHO draft | 1,034 | 96 | 9 | 13 | 117 | 0.91 | 0.75 | 0.37 | 0.78 | 0.11 |
| 2 | No filters | 919 | 96 | 9 | 14 | 118 | 0.91 | 0.75 | 0.40 | 0.79 | 0.13 |
| 3 | No filters | 923 | 98 | 9 | 14 | 120 | 0.93 | 0.75 | 0.40 | 0.80 | 0.13 |
| 4 | No filters | 1,327 | 98 | 9 | 16 | 121 | 0.93 | 0.75 | 0.46 | 0.81 | 0.09 |
| 5 | No filters | 923 | 98 | 9 | 14 | 120 | 0.93 | 0.75 | 0.40 | 0.80 | 0.13 |
| 6 | No filters | 1,473 | 101 | 9 | 26 | 134 | 0.96 | 0.75 | 0.74 | 0.89 | 0.09 |
|  | Filter Humans | 1,329 | 97 | 9 | 26 | 130 | 0.92 | 0.75 | 0.74 | 0.87 | 0.10 |

1. **Medline search strategy**

Ovid MEDLINE(R) and Epub Ahead of Print, In-Process & Other Non-Indexed Citations, Daily and Versions(R) <1946 to October 15, 2020> - Searched 16^th^ October 2020

| **#** | **String** | **Records** |
| --- | --- | --- |
| 1 | "Cell Phone Use"/ae, sn, td [Adverse Effects, Statistics & Numerical Data, Trends] | (112) |
| 2 | electromagnetic fields/ae or electromagnetic radiation/ or radio waves/ae or microwaves/ae | (6812) |
| 3 | (((mobile or cell*) adj1 (phone* or telephone* or technolog* or device*)) or smartphone* or smart-phone* or cellphone* or ((radiofrequency or high-frequency or microwave*) adj3 (field* or electromagnetic or expos* or radiation or mast or masts or antenna*)) or RF-EMF or ((radio or broadcast* or telegraph) adj2 (transmitter* or mast or masts or antenna* or operator*)) or broadcasting or "base station*" or radar or "plastic sealer*").ti,ab,kw. | (45233) |
| 4 | or/1-3 | (49847) |
| 5 | etiology.fs. or (etiolog* or aetiolog*).ti,ab,kw. | (2755072) |
| 6 | epidemiologic studies/ or case-control studies/ or retrospective studies/ or cohort studies/ or follow-up studies/ or longitudinal studies/ or prospective studies/ or historically controlled study/ | (2270427) |
| 7 | "surveys and questionnaires"/ or health surveys/ or population surveillance/ or public health surveillance/ or sentinel surveillance/ or patient health questionnaire/ or self report/ or risk factors/ | (1370878) |
| 8 | comparative study.pt. | (1872891) |
| 9 | ("case reference" or "risk factor*" or "cohort study" or "case control" or case-control or cohort or ((prospective or retrospective or follow-up or longitudinal) adj2 (study or analysis)) or confounding or confounder* or questionnaire*).ti,ab,kw. | (2121756) |
| 10 | or/5-9 | (7340984) |
| 11 | (occupational exposure/ or radiation exposure/) and (radio waves/ or microwaves/ or radiation, nonionizing/ae) | (333) |
| 12 | ((occupational or domestic or residential or environmental) adj1 exposure adj6 (radio wave* or radiowave* or microwave* or non-ionising radiation or non-ionizing radiation or electromagnetic field*)).ti,ab,kw. | (188) |
| 13 | or/11-12 | (494) |
| 14 | exp Ablation Techniques/ or ablation*.ti,ab,kw. | (174116) |
| 15 | 13 not 14 | (488) |
| 16 | Neoplasms/an, ep, et, mo, pc, sn [Analysis, Epidemiology, Etiology, Mortality, Prevention & Control, Statistics & Numerical Data] | (72162) |
| 17 | brain neoplasms/ or cerebral ventricle neoplasms/ or choroid plexus neoplasms/ or papilloma, choroid plexus/ or infratentorial neoplasms/ or brain stem neoplasms/ or diffuse intrinsic pontine glioma/ or cerebellar neoplasms/ or neurocytoma/ or pinealoma/ or supratentorial neoplasms/ or hypothalamic neoplasms/ or pituitary neoplasms/ or meningeal neoplasms/ or meningeal carcinomatosis/ or meningioma/ or cranial nerve neoplasms/ or neuroma, acoustic/ or optic nerve neoplasms/ or optic nerve glioma/ | (178146) |
| 18 | leukemia/ or leukemia, hairy cell/ or exp leukemia, lymphoid/ or leukemia, mast-cell/ or exp leukemia, myeloid/ or leukemia, plasma cell/ | (211504) |
| 19 | exp Lymphoma, Non-Hodgkin/ | (102408) |
| 20 | Melanoma/ | (85037) |
| 21 | Testicular Neoplasms/ | (24338) |
| 22 | (Neoplasms, Radiation-Induced/ or leukemia, radiation-induced/) and radiation, nonionizing/ | (38) |
| 23 | exp Eye Neoplasms/ | (40580) |
| 24 | (cancer* or neoplasm* or tumour or tumours or tumor or tumors or glioma or meningioma or acoustic neuroma or vestibular schwannoma or melanoma or leukemia or leukaemia or non-hodgkin lymphoma* or (cause-specific adj1 (mortality or death*))).ti,ab,kw. | (3146722) |
| 25 | or/16-24 | (3337830) |
| **26** | **((4 and 10) or 15) and 25** | **(2010)** |

1. **Embase search strategy**

Embase Classic+Embase (Ovid) <1947 to 2020 October 15> - Searched 19^th^ October 2020

| **#** | **String** | **Records** |
| --- | --- | --- |
| 1 | "cell phone use"/ or electromagnetism/ or electromagnetic radiation/ or microwave radiation/ or radiofrequency radiation/ | (41687) |
| 2 | (((mobile or cell*) adj1 (phone* or telephone* or technolog* or device*)) or smartphone* or smart-phone* or cellphone* or ((radiofrequency or high-frequency or microwave*) adj3 (field* or electromagnetic or expos* or radiation or mast or masts or antenna*)) or RF-EMF or ((radio or broadcast* or telegraph) adj2 (transmitter* or mast or masts or antenna* or operator*)) or broadcasting or "base station*" or radar or "plastic sealer*").ti,ab,kw. | (57128) |
| 3 | or/1-2 | (91768) |
| 4 | etiology.fs. or (etiolog* or aetiolog*).ti,ab,kw. | (2889279) |
| 5 | cancer epidemiology/ or case control study/ or hospital based case control study/ or population based case control study/ or longitudinal study/ or prospective study/ or retrospective study/ or "evaluation and follow up"/ or follow up/ or cohort analysis/ or health survey/ or questionnaire/ or self report/ or sentinel surveillance/ or attributable risk/ or risk factor/ or comparative study/ | (5557585) |
| 6 | ("case reference" or "risk factor*" or "cohort study" or "case control" or case-control or cohort or ((prospective or retrospective or follow-up or longitudinal or comparative) adj2 (study or analysis)) or confounding or confounder* or questionnaire*).ti,ab,kw. | (3444821) |
| 7 | or/4-6 | (8769865) |
| 8 | (occupational exposure/et, pc or radiation exposure/) and (electromagnetic radiation/ or radio waves/ae or microwaves/ae) | (1040) |
| 9 | ((occupational or domestic or residential or environmental) adj1 exposure adj6 (radio wave* or radiowave* or microwave* or non-ionising radiation or non-ionizing radiation or electromagnetic field*)).ti,ab,kw. | (253) |
| 10 | or/8-9 | (1283) |
| 11 | radiofrequency ablation/ or catheter ablation/ or radiofrequency ablation device/ or tumor ablation/ or radiofrequency catheter ablation/ or ablation therapy/ or endometrium ablation/ or ablation catheter/ | (87222) |
| 12 | ablation*.ti,ab,kw. | (152093) |
| 13 | or/11-12 | (173518) |
| 14 | 10 not 13 | (1275) |
| 15 | brain cancer/ or brain leukemia/ or brain lymphoma/ | (13466) |
| 16 | childhood cancer/ or childhood leukemia/ | (47235) |
| 17 | malignant neurilemoma/ | (749) |
| 18 | exp nonhodgkin lymphoma/ | (181386) |
| 19 | melanoma skin cancer/ or amelanotic melanoma/ or malignant lentigo/ | (4254) |
| 20 | choroid plexus papilloma/ | (1329) |
| 21 | cerebellum tumor/ | (4624) |
| 22 | pontine glioma/ or glioma/ or optic nerve glioma/ | (64340) |
| 23 | neuroepithelioma/ or olfactory neuroepithelioma/ | (3780) |
| 24 | pineal body tumor/ | (3364) |
| 25 | meningioma/ | (34403) |
| 26 | acoustic neuroma/ | (2315) |
| 27 | exp leukemia/ | (342486) |
| 28 | exp testis cancer/ | (22549) |
| 29 | radiation induced neoplasm/ and (nonionising or non-ionising or nonionizing or non-ionizing).ti,ab,kw. | (10) |
| 30 | neoplasm/ep, et, pc | (20627) |
| 31 | malignant neoplasm/ep, et, pc | (3893) |
| 32 | (cancer* or neoplasm* or tumour or tumours or tumor or tumors or glioma or meningioma or acoustic neuroma or vestibular schwannoma or melanoma or pinealoma or neurocytoma or leukemia or leukaemia or non-hodgkin* lymphoma* or (cause-specific adj1 (mortality or death*))).ti,ab,kw. | (4553442) |
| 33 | or/15-32 | (4716609) |
| **34** | **((3 and 7) or 14) and 33** | **(2652)** |

1. **Medline search strategy for cancer studies in selected occupational groups**

Ovid MEDLINE(R) and Epub Ahead of Print, In-Process & Other Non-Indexed Citations, Daily and Versions(R) <1946 to December 14, 2020> Searched 15^th^ December 2020

| 1 | etiology.fs. or (etiolog* or aetiolog*).ti,ab,kw. | (2773455) |
| --- | --- | --- |
| 2 | epidemiologic studies/ or case-control studies/ or retrospective studies/ or cohort studies/ or follow-up studies/ or longitudinal studies/ or prospective studies/ or historically controlled study/ | (2298469) |
| 3 | "surveys and questionnaires"/ or health surveys/ or population surveillance/ or public health surveillance/ or sentinel surveillance/ or patient health questionnaire/ or self report/ or risk factors/ | (1387409) |
| 4 | comparative study.pt. | (1878361) |
| 5 | ("case reference" or "risk factor*" or "cohort study" or "case control" or case-control or cohort or ((prospective or retrospective or follow-up or longitudinal) adj2 (study or analysis)) or confounding or confounder* or questionnaire*).ti,ab,kw. | (2168199) |
| 6 | or/1-5 | (7418774) |
| 7 | Neoplasms/an, ep, et, mo, pc, sn [Analysis, Epidemiology, Etiology, Mortality, Prevention & Control, Statistics & Numerical Data] | (72762) |
| 8 | brain neoplasms/ or cerebral ventricle neoplasms/ or choroid plexus neoplasms/ or papilloma, choroid plexus/ or infratentorial neoplasms/ or brain stem neoplasms/ or diffuse intrinsic pontine glioma/ or cerebellar neoplasms/ or neurocytoma/ or pinealoma/ or supratentorial neoplasms/ or hypothalamic neoplasms/ or pituitary neoplasms/ or meningeal neoplasms/ or meningeal carcinomatosis/ or meningioma/ or cranial nerve neoplasms/ or neuroma, acoustic/ or optic nerve neoplasms/ or optic nerve glioma/ | (179339) |
| 9 | leukemia/ or leukemia, hairy cell/ or exp leukemia, lymphoid/ or leukemia, mast-cell/ or exp leukemia, myeloid/ or leukemia, plasma cell/ | (212438) |
| 10 | ((brain adj3 (cancer* or neoplasm* or tumour or tumours or tumor or tumors )) or glioma or meningioma or acoustic neuroma or vestibular schwannoma or leukemi* or leukaemi*).ti,ab,kw. | (379969) |
| 11 | or/7-10 | (604257) |
| 12 | firefighters/ or police/ or physical therapist assistants/ or physical therapists/ | (8452) |
| 13 | Pilots/ and (ship* or marine or maritime or coastal or sea or ocean or waterway* or naval or navy).ti,ab,kw. | (32) |
| 14 | (pilot* adj3 (ship* or marine or maritime or coastal or sea or ocean or waterway* or naval or navy)).ti,ab,kw. | (138) |
| 15 | (prison* adj2 (guard* or warden* or jailer* or gaoler* or officer*)).ti,ab,kw. | (216) |
| 16 | ((police adj2 (officer* or man or men or woman or women or constable*)) or policeman or policemen or policewoman or policewomen).ti,ab,kw. | (3140) |
| 17 | ((fire adj2 (fighter* or man or men or woman or women or officer*)) or fireman or firemen or firewoman or firewomen).ti,ab,kw. | (736) |
| 18 | (((ship* or sea or deck or naval or navy or maritime) adj2 (officer* or captain*)) or sailor*).ti,ab,kw. | (1272) |
| 19 | (air adj2 ("traffic controller*" or "traffic operator*")).ti,ab,kw. | (272) |
| 20 | ((physical adj2 therapist*) or physiotherapist*).ti,ab,kw. | (14737) |
| 21 | ((broadcast* or audiovisual or "audio visual" or av or "information and communication* technology" or ICT or television* or tv or radio) adj3 (technician* or installer* or servic*)).ti,ab,kw. | (466) |
| 22 | or/12-21 | (26169) |
| **23** | **6 and 11 and 22** | **(103)** |

1. **Embase search strategy for cancer studies in selected occupational groups**

Embase (Ovid) <1980 to 2020 Week 50> Searched 15^th^ December 2020

| 1 | etiology.fs. or (etiolog* or aetiolog*).ti,ab,kw. | (2574690) |
| --- | --- | --- |
| 2 | cancer epidemiology/ or case control study/ or hospital based case control study/ or population based case control study/ or longitudinal study/ or prospective study/ or retrospective study/ or "evaluation and follow up"/ or follow up/ or cohort analysis/ or health survey/ or questionnaire/ or self report/ or sentinel surveillance/ or attributable risk/ or risk factor/ or comparative study/ | (5471966) |
| 3 | ("case reference" or "risk factor*" or "cohort study" or "case control" or case-control or cohort or ((prospective or retrospective or follow-up or longitudinal or comparative) adj2 (study or analysis)) or confounding or confounder* or questionnaire*).ti,ab,kw. | (3429243) |
| 4 | or/1-3 | (8353799) |
| 5 | neoplasm/di, ep, et, pc [Diagnosis, Epidemiology, Etiology, Prevention] | (24971) |
| 6 | brain cancer/ or brain leukemia/ or brain lymphoma/ or childhood leukemia/ or malignant neurilemoma/ or choroid plexus papilloma/ or cerebellum tumor/ or pontine glioma/ or glioma/ or optic nerve glioma/ or neuroepithelioma/ or olfactory neuroepithelioma/ or pineal body tumor/ or meningioma/ or acoustic neuroma/ or exp leukemia/ | (393239) |
| 7 | ((brain adj3 (cancer* or neoplasm* or tumour or tumours or tumor or tumors)) or glioma or meningioma or acoustic neuroma or vestibular schwannoma or leukemi* or leukaemi*).ti,ab,kw. | (489779) |
| 8 | or/5-7 | (615688) |
| 9 | (pilot* adj3 (ship* or marine or maritime or coastal or sea or ocean or waterway* or naval or navy)).ti,ab,kw. | (158) |
| 10 | (prison* adj2 (guard* or warden* or jailer* or gaoler* or officer*)).ti,ab,kw. | (277) |
| 11 | ((police adj2 (officer* or man or men or woman or women or constable*)) or policeman or policemen or policewoman or policewomen).ti,ab,kw. | (3839) |
| 12 | ((fire adj2 (fighter* or man or men or woman or women or officer*)) or fireman or firemen or firewoman or firewomen).ti,ab,kw. | (993) |
| 13 | (((ship* or sea or deck or naval or navy or maritime) adj2 (officer* or captain*)) or sailor*).ti,ab,kw. | (1233) |
| 14 | (air adj2 ("traffic controller*" or "traffic operator*")).ti,ab,kw. | (312) |
| 15 | ((physical adj2 therapist*) or physiotherapist*).ti,ab,kw. | (25845) |
| 16 | ((broadcast* or audiovisual or "audio visual" or av or "information and communication* technology" or ICT or television* or tv or radio) adj3 (technician* or installer* or servic*)).ti,ab,kw. | (489) |
| 17 | fire fighter/ or police/ or physiotherapist/ or physiotherapist assistant/ or sailor/ or air traffic controller/ | (38351) |
| 18 | or/9-17 | (50965) |
| **19** | **4 and 8 and 18** | **(110)** |

1. **References**

AGNIR. Health effects from radiofrequency electromagnetic fields. London: Health Protection Agency; 2012.

ANSES. Radiofréquences et santé. Mise à jour de l’expertise. Maisons-Alfort: Agence nationale de sécurité sanitaire de l’alimentation de l’environnement et du travail; 2013.

ANSES. Exposition aux radiofréquences et santé des enfants. Maisons-Alfort: Agence nationale de sécurité sanitaire de l’alimentation de l’environnement et du travail; 2016.

ARPANSA. Review of radiofrequency health effects research: Scientific literature 2000-2012. in: Radiofrequency Expert Panel, ed. Technical Report Series No 164. Yallambie: Australian Radiation Protection and Nuclear Safety Agency; 2014.

CCARS. Informe sobre Radiofrecuencia y Salud (2013-2016). Madrid: Comité Científico Asesor en Radiofrecuencias y Salud. Colegio Oficial de Ingenieros de Telecomunicación (COIT); 2017.

Demers, P., Findlay, R., Foster, K.R., Kolb, B., Moulder, J., Nicol, A.M., Prato, F., Stam, R., Royal Society of Canada Expert Panel. Expert Panel Report on a review of Safety Code 6 (2013): Health Canada’s safety limits for exposure to radiofrequency fields. Ottawa: Royal Society of Canada; 2014.

FDA. Review of published literature between 2008 and 2018 of relevance to radiofrequency radiation and cancer. Food and Drug Administration - Center for Devices and Radiological Health; 2020.

Hausner, E., Waffenschmidt, S., Kaiser, T., Simon, M. Routine development of objectively derived search strategies. Syst Rev 2012;1:19.

HCN. Mobile phones and cancer Part 3. Update and overall conclusions from epidemiological and animal studies. The Hague: Health Council of the Netherlands; 2016.

IARC. Non-ionizing radiation, Part 2: Radiofrequency electromagnetic fields. IARC Monogr Eval Carcinog Risks Hum, vol 102. Lyon: IARC Press; 2013.

ICHENF. Interagency Committee on the Health Effects of Non-Ionising Fields - Report to Ministers 2018. Wellington: Ministry of Health; 2018.

ICNIRP. Guidelines for Limiting Exposure to Electromagnetic Fields (100 kHz to 300 GHz). Health Phys 2020;118:483-524.

SCENIHR. Potential health effects of exposure to electromagnetic fields (EMF). Luxembourg: European Commission's Scientific Committee on Emerging and Newly Identified Health Risks; 2015.

SSM. Recent Research on EMF and Health Risk: Eighth report from SSM's Scientific Council on Electromagnetic Fields. Stockholm: Swedish Radiation Safety Authority; 2013.

SSM. Recent Research on EMF and Health Risk: Ninth report from SSM’s Scientific Council on Electromagnetic Fields, 2014. Stockholm: Swedish Radiation Safety Authority; 2014.

SSM. Recent Research on EMF and Health Risk: Tenth report from SSM’s Scientific Council on Electromagnetic Fields, 2015. Stockholm: Swedish Radiation Safety Authority; 2015.

SSM. Recent Research on EMF and Health Risk: Eleventh report from SSM's Scientific Council on Electromagnetic Fields, 2016. Stockholm: Swedish Radiation Safety Authority; 2016.

SSM. Recent Research on EMF and Health Risk: Twelfth report from SSM's Scientific Council on Electromagnetic Fields, 2017. Stockholm: Swedish Radiation Safety Authority; 2018.

SSM. Recent Research on EMF and Health Risk: Thirteenth report from SSM's Scientific Council on Electromagnetic Fields, 2018. Stockholm: Swedish Radiation Safety Authority; 2019.

WHO. Radio Frequency fields: Environmental Health Criteria Monograph. Consultation on the scientific review for the upcoming WHO Environmental Health Criteria (30 September -15 December 2014). Chapter 12 Cancer. 2014.
