# Supplementary material for: The effect of exposure to radiofrequency fields on cancer risk in the general and working population: A protocol for a systematic review of human observational studies
Source: Environ Int. 2021 Dec;157:106828. doi: 10.1016/j.envint.2021.106828 (PMC8484862; doi:10.1016/j.envint.2021.106828)
Supplement: Supplementary data 4 [file mmc4.docx]

Systematic review of human observational studies on the effect of exposure
to radiofrequency fields and cancer risk in the general and working population

**Protocol for the assessment of risk of bias**

**Table of content**

[INTRODUCTION 2](#_Toc74474048)

[Methods 2](#_Toc74474049)

[I.1. Bias domains and questions 2](#_Toc74474050)

[I.2. Rating levels and criteria 3](#_Toc74474051)

[I.3. Direction and magnitude of bias 3](#_Toc74474052)

[I.4. Issues not considered as bias elements 4](#_Toc74474053)

[I.5. Customization of the RoB assessment tool 5](#_Toc74474054)

[I.6. Pilot-testing of the RoB assessment tool 5](#_Toc74474055)

[I.7. Conduct and management of RoB assessment process 8](#_Toc74474056)

[TAILORED RATING INSTRUCTIONS AND ANSWER OPTION FORMS 8](#_Toc74474057)

[II.1. Confounding 8](#_Toc74474058)

[II.2. Selection biases 11](#_Toc74474059)

[II.3. Information biases 20](#_Toc74474060)

[II.4. Confidence in exposure characterization 20](#_Toc74474061)

[II.5. Confidence in outcome assessment 32](#_Toc74474062)

[II.6. Selective reporting 35](#_Toc74474063)

[II.7. Appropriateness of statistical methods 36](#_Toc74474064)

[APPENDIX 36](#_Toc74474065)

[III.1. Analytical framework used to identify critical potential confounders 36](#_Toc74474066)

[REFERENCES 40](#_Toc74474067)

# INTRODUCTION

To assess the study’s internal validity we will use the OHAT Risk of Bias Rating Tool for Human and Animal Studies (NTP-OHAT 2015), following the methodological indications provided by the Handbook for Conducting a Literature-Based Health Assessment Using OHAT Approach for Systematic Review and Evidence Integration [(NTP-OHAT 2019), pp. 33-43]. The choice of the method was based on the considerations below.

The WHO recommended a harmonized approach to risk-of-bias assessment (RoB) in the commissioned systematic reviews of the effect of exposure to radiofrequency electromagnetic field (RF-EMF) on selected health outcomes (Verbeek et al. 2021), especially in those addressing the evidence provided by human observational studies on risks of cancer, adverse reproductive effects, impaired cognition, and symptoms.

Out of 62 tools for assessing methodological quality of human observational studies identified in a recent systematic review (Wang et al. 2020), almost half calculate a quality score, which is a practice not recommended for systematic reviews of observational studies of interventions, aetiology, or environmental health topics (Dekkers et al. 2019; Sterne et al. 2019; Whaley et al. 2020). Among the 25 tools aimed at assessing the study’s potential for bias, only four were specifically developed for the environmental research area.^[[1]](#footnote-1)^ Of these, two focus on single outcomes (NTP-ORoC 2015; Roth and Wilks 2014). The other two share a cohesive approach to RoB assessment across multiple evidence streams and topics (NTP-OHAT 2015; Woodruff and Sutton 2014), but only one of them (NTP-OHAT 2015) is accompanied by a detailed guidance to all stages of a systematic review (NTP-OHAT 2019). A tool with similar features, ROBIN-E^[[2]](#footnote-2)^, is under development (Steenland et al. 2020).

The OHAT RoB assessment method has already been adopted by different international entities, and used in several systematic reviews. Here we present our adaption of the OHAT RoB method to the assessment of the internal validity of human observational studies investigating the effect of exposure to RF-EMF on cancer risk in the general and working population.

# Methods

1. **Bias domains and questions**

In line with Cochrane’s approach (Boutron et al. 2019), OHAT defines bias as a systematic error (or deviation from the truth) in results or inferences (NTP-OHAT 2019).

The purpose of the OHAT RoB assessment is to evaluate whether the design and conduct of a study compromised the credibility of the link between exposure and outcome (NTP-OHAT 2015).

The OHAT RoB rating tool uses a single set of questions to coherently assess risk-of-bias across various evidence streams (human studies, studies on animal models, and mechanistic studies). There are 11 risk-of-bias questions under six bias domains. Individual questions are applicable to one to six study designs (experimental animal studies; human controlled-exposure studies; cohort studies; case-control studies, cross-sectional/ecological studies; case series), with four questions applicable to any design (NTP-OHAT 2015; 2019).

Six bias questions, plus a seventh item allowing the possibility to consider “Other threats to validity”, apply to cohort and case-control studies, eligible for inclusion in our systematic review (Table 1).

**Table 1. Bias domains and questions applicable to cohort and case-control studies** (NTP-OHAT 2015; 2019)

| **Bias domain** | **Question** | |
| --- | --- | --- |
| Selection | Q3 | Did selection of study participants result in appropriate comparison groups? |
| Confounding | Q4 | Did the study design or analysis account for important confounding and modifying variables? |
| Attrition^†^ | Q7 | Were outcome data complete without attrition or exclusion from analysis? |
| Detection^‡^ | Q8 | Can we be confident in the exposure characterization? |
|  | Q9 | Can we be confident in the outcome assessment? |
| Selective reporting | Q10 | Were all measured outcomes reported? |
| Others | Q11 | Were there no other potential threats to internal validity? |
| Alternative labels: ^†^Exclusion/Missing data; ^‡^Detection/Information/Measurement [(NTP-OHAT 2019), p. 34]. | | |

1. **Rating levels and criteria**

The response options for each RoB question foresee a four-level rating of the potential for bias (Table 2).

**Table 2. Answer options** [(NTP-OHAT 2015), p. 4; (NTP-OHAT 2019), p. 36]

| **Definitely Low** | There is direct evidence of low risk of bias practices.  (May include specific examples of relevant low risk of bias practices). |
| --- | --- |
| **Probably Low** | There is indirect evidence of low risk of bias practices OR it is deemed that deviations from low risk of bias practices for these criteria during the study would not appreciably bias results, including consideration of direction and magnitude of bias. |
| **Probably High** | There is indirect evidence of high risk of bias practices OR there is insufficient information (e.g., not reported or “NR”) provided about relevant risk of bias practices. |
| **Definitely High** | There is direct evidence of high risk of bias practice.  (May include specific examples of relevant high risk of bias practices). |

The input from *direct* *vs* *indirect* evidence (Table 2) is the discriminating criterion between adjacent categories (definitely *vs* probably) of low or high risk of bias. Guidance on what to consider as direct and indirect evidence is included in our tailored instructions to rating and/or in the customized answer options.

1. **Direction and magnitude of bias**

According to OHAT, the form used by the assessors during the RoB process should include an option to judge the direction of putative bias for each question or domain. The classification of the bias direction will depend on the type of bias. For some questions or domains, the bias is most easily thought of as directional towards or away from the null, and for others (confounding, selection bias, and forms of measurement bias such as differential misclassification), the bias is thought of as an increase or decrease in the effect estimate independent of the null (NTP-OHAT 2019).

When the bias rating is probably high (-) or definitely high (++), we will indicate whether the bias direction is:

- *Upward,* resulting in apparent or overestimated effects of the exposure;
- *Towards null*, resulting in undetected or underestimated effects of the exposure;
- *Uncertain,* due to lack of ad hoc bias studies or generalizable external evidence.

The incomplete bias direction labelling (with missing “downward” or “away from null” categories) is intentional, and reflects the aim of a systematic review conducted in the framework of a hazard assessment.

Predicting the bias direction may be impossible in the lack of ad hoc bias studies. This is especially the case for selection bias (where the direction could be anticipated, pending the availability of required data; see § II.2). Moreover, the study findings can be affected by multiple competing biases, whose net effect would be difficult to anticipate in the lack of comprehensive bias modelling studies.

In the following instances, the bias direction will be easier to predict:

- Nondifferential and nondependent misclassification of the exposure or the outcome, generally towards null (or no bias in the scenario of a true null association);
- The healthy worker effect, usually downward;
- Exposure misclassification dependent on the outcome (*recall bias*), usually upward for self-reported data;
- Confounding variables acting in a predictable direction. Based on a simple rule, only valid for dichotomous variables, uncontrolled confounding will result in an upward bias when the associations of a confounder (C) with the exposure (E) and the outcome (D) have the same sign (either both positive, or both negative); and in a downward bias when the C-E and C-D associations are in opposite directions (Hernán and Robin 2020);
- Detection bias in studies of mobile phone use and acoustic neuroma (upward).

In line with OHAT’s indication and COSTER recommendation 5.5, the RoB assessors will not attempt to guess the direction of bias in the absence of a clear rationale with scientific support (NTP-OHAT 2019; Whaley et al. 2020).

During the RoB pilot study (see § 1.6), we will try to develop a rule for defaulting to the “Uncertain” category unless an evidence threshold has been met for a judgement of direction.

The rating criteria for the “probably low” RoB level include consideration of the bias magnitude (Table 2).

OHAT underlines the relevance of the issue, but notes that “it is usually impossible to know to what extent biases have affected the results of a particular study” [(NTP-OHAT 2019), p. 33]. When the amount of bias can be confidently anticipated [e.g., from bias studies or sensitivity analyses conducted in the framework of a given study, or from other relevant validation studies (van Smeden et al. 2020)], we will use this information to modify a rating of probably high risk of bias based on main criteria, according to the rules below.

| **Rating based on main criteria** | **Presumed bias magnitude based on ancillary evidence** | |
| --- | --- | --- |
|  | **Modest** | **Substantial** |
| Probably High Risk of Bias | Rate Probably Low | Rate Definitely High |
| *Modest =* the bias factor cannot explain out the observed exposure-disease association, or it cannot change a null association into a positive/negative one; *Substantial* = the bias factor can explain out the observed exposure-disease association, or change a null association into a positive/negative one. | | |

The assessors will record and justify consideration of bias direction and magnitude in the RoB assessment form.

1. **Issues not considered as bias elements**

Similarly to Cochrane’s approach (Boutron et al. 2019), the OHAT’s practice is not to consider funding source and related financial conflicts of interests (COI) as a specific risk of bias element. An important caveat in assessing the impact of COI on the reliability of individual study’s findings and/or systematic review’s conclusions, is that newer studies may appear to be at greater risk than older studies because of changes in journal reporting standards [(NTP-OHAT 2019), p. 40]. We will collect information about funding source during data extraction and consider it at the stage of evidence appraisal, as a potential source of publication bias (see Annex 6).

Study sensitivity (the ability to detect an effect of the exposure if present) is an item of the RoB tool developed by the Office of the Report on Carcinogens (NTP-ORoC 2015). The underlying rationale is that the utility of a study for cancer evaluation may be compromised by several factors (low statistical power; inconsequential exposure in terms of frequency, level, and/or duration; exposure outside the appropriate time window; limited range of exposure levels and duration; length of follow-up shorter than the average induction period) requiring an integrated assessment. OHAT acknowledges that a limited exposure duration or a too short interval between exposure and outcome assessment would be inappropriate for evaluating the association with a chronic disease. However, unless attempts to harmonize methods with other organizations indicate preference for a different strategy, OHAT will consider these issues in the PECO statement (excluding studies where the timing of the exposure or outcome assessment are clearly inappropriate for consideration in an evaluation), or during evidence appraisal as part of indirectness [(NTP-OHAT 2019), p. 40]. We will conform to this approach.

1. **Customization of the RoB assessment tool**

Versatility is a distinctive feature of the OHAT’s RoB tool. It can be applied to many research questions, but in all cases must be tailored to the specific topic of a given systematic review [(NTP-OHAT 2015), p. 3].

As foreseen by OHAT [(NTP-OHAT 2019), p. 40], the key-elements requiring customized rating instructions and answer option forms were: confounding, selection bias, and confidence in exposure and outcome assessment.

We structured the customized answer option forms according to the most relevant features to consider for each type of bias, that is, study design for selection and outcome-information biases (Q3, Q7, Q9), and exposure-type for the exposure-information bias (Q8). For confounding (Q4), we envisaged a single form, with indications about critical confounders for each exposure-outcome pair of primary interest provided in the rating instructions. For the remaining questions (Q10, Q11), the tailored answer options apply to any study design and topic. The answer option form relating to exposure assessment (Q8) include appendices where the assessors are requested to specify to which exposure-disease contrast, and to what type of measurement error, the rating refers to.

Under “Other sources of bias” (Q11), we consider the *appropriateness of statistical methods* (Yes/No format, with details to be provided if the answer is “No”). We do not address *adherence to study protocol*, as protocol registration/publication is still uncommon practice in the environmental research area. Post-hoc analyses, when identifiable as such, are considered under the domain of selective reporting (Q10) in our customized RoB tool.

1. **Pilot-testing of the RoB assessment tool**

To check and improve the clarity of the rating guidance and consistency in judgment among assessors, OHAT envisages that RoB assessors be trained using project-specific instructions in an initial pilot-testing phase, undertaken on a small subset of the included studies [(NTP-OHAT 2019), pp. 42-43].

We will carry out a proper pilot study of the customized RoB tool early during the systematic review. The underlying reason is that only after completion of study selection it will be possible to appreciate the main features of the included studies, and perform the pilot-testing on a study sample representative of the datasets pertaining to each component of our systematic review (SR-A, SR-B, SR-C).

However, we did test a preliminary version of the tailored RoB tool. All team members entrusted to perform the RoB assessment evaluated two studies (one cohort and one case-control study). The results of this pre-pilot (Table 3) were discussed in a web meeting, from which valuable insights emerged on how to correct the identified shortcomings in the rating instructions, answer option forms, and the provisional assessment form (Table 4).

**Table 3. Results of the pre-pilot study of the customized RoB tool**

| **Bias Question** | | **Cohort Study** | | | | | | | |
| --- | --- | --- | --- | --- | --- | --- | --- | --- | --- |
|  |  | **Rater1** | | **Rater2** | **Rater3** | **Rater4** | | **Rater5** | **Rater6** |
| Q3 | Selection | (++) | | (+) | (+) | (++) | | (+) | (++) |
| Q4 | Confounding | (++) | | (+) | (+) | (++) | | (++) | (++) |
| Q7 | Attrition | (+) | | (NR) | (++) | (++) | | (+) | (++) |
| Q8 | Exposure | (+) | | (+) | (--) | (++)^†^ | (-)^‡^ | (+) | (++) |
| Q9 | Outcome | (++) | | (++) | (++) | (++) | | (++) | (+) |
| Q10 | SelectiveReporting | (++) | | (+) | (++) | (++) | | (++) | (++) |
| Q11_a_ | RangeVariation | (++) | (+) | (-) | NA | (-) | (++)^§^ | (+) | (++) |
| Q11_b_ | InternalConsistency | (++) | | NA | NA | (++) | | (++) | (++) |
| Q11_c_ | StatisticalMethods | YES | | YES | YES | YES | | YES | (++) |
| **Bias Question** | | **Case-Control Study** | | | | | | | |
|  |  | **Rater1** | | **Rater2** | **Rater3** | **Rater4** | | **Rater5** | **Rater6** |
| Q3 | Selection | n.g. | | (-) | (++) | (+) U | | (+) | (+) |
| Q4 | Confounding | (++) | | (-) | (++) | (++) | | (++) | (++) |
| Q7 | Attrition | (++) | | (++) | n.g. | (++) | | (++) | (++) |
| Q8 | Exposure | (+) | | (-) | (-) | (-) | | (+) | (+) |
| Q9 | Outcome | (++) | | (++) | (+) | (+) | | (++) | (++) |
| Q10 | SelectiveReporting | (++) | | (++) | (++) | (++) | | (++) | (++) |
| Q11_a_ | RangeVariation | (++) | | (++) | (+) | (++) | | (++) | (++) |
| Q11_b_ | InternalConsistency | (--) | | (++) | (++)? | (++) | | (--) | NA |
| Q11_c_ | StatisticalMethods | YES | | YES | YES | YES | | YES | YES |
| **(+ +)** = Definitely high risk of bias; **(+)** = Probably low risk of bias; **(-)** = Probably high risk of bias **(- -); (NR)** = Probably high risk of bias due to insufficient information provided about relevant risk of bias practices; **(- -)** = Definitely high risk of bias.  **Reasons behind the double rating, or missing rating**: ^†^Applies to differential exposure misclassification; ^‡^Applies to random exposure measurement errors; ^§^Low range of variation does not imply bias; NA = question considered not applicable; n.g. = not given, due to uncertainties on how to interpret rating instructions. | | | | | | | | | |

**Table 4. Provisional RoB assessment form used in the pre-pilot study**

| **Study ID ……………**  **SR** A □ B □ C □ | | **Reference** | **Design**  Cohort □ Case-Control □ | | **Assessor** | **Date** |
| --- | --- | --- | --- | --- | --- | --- |
|  | | | | | | |
| **Question** | **Any ambiguities?** | | **RoB Rating** | **Bias direction** | **Justify rating** | |
| Q3 Selection |  | | (++) | - |  | |
|  |  |  | (+) | - |  |  |
|  |  |  | (-) | Upward □ |  |  |
|  |  |  | (NR) | Downward □ |  |  |
|  |  |  | (--) | Uncertain □ |  |  |
| Q4 Confounding |  | | (++) | - |  | |
|  |  |  | (+) | - |  |  |
|  |  |  | (-) | Upward □ |  |  |
|  |  |  | (NR) | Downward □ |  |  |
|  |  |  | (--) | Uncertain □ |  |  |
| Q7 Attrition |  | | (++) | - |  | |
|  |  |  | (+) | - |  |  |
|  |  |  | (-) | Upward □ |  |  |
|  |  |  | (NR) | Downward □ |  |  |
|  |  |  | (--) | Uncertain □ |  |  |
| Q8 Exposure |  | | (++) | - |  | |
|  |  |  | (+) | - |  |  |
|  |  |  | (-) | Upward □ |  |  |
|  |  |  | (NR) | Downward □ |  |  |
|  |  |  | (--) | Uncertain □ |  |  |
| Q9 Outcome |  | | (++) | - |  | |
|  |  |  | (+) | - |  |  |
|  |  |  | (-) | Upward □ |  |  |
|  |  |  | (NR) | Downward □ |  |  |
|  |  |  | (--) | Uncertain □ |  |  |
| Q10 Selective reporting |  | | (++) | - |  | |
|  |  |  | (+) | - |  |  |
|  |  |  | (-) | Upward □ |  |  |
|  |  |  | (NR) | Downward □ |  |  |
|  |  |  | (--) | Uncertain □ |  |  |
| Q11_a_ Range of variation |  | | (++) | NA |  | |
|  |  |  | (+) |  |  |  |
|  |  |  | (-) |  |  |  |
|  |  |  | (--) |  |  |  |
| Q11_b_ Internal consistency |  | | (++) | NA |  | |
|  |  |  | (+) |  |  |  |
|  |  |  | (-) |  |  |  |
|  |  |  | (--) |  |  |  |
| Q11_c_ Statistical Methods |  | | Yes | NA | If “No”, provide details | |
|  |  |  | No |  |  |  |
| **Any ambiguities?** = Report any ambiguities in criteria for RoB rating. **RoB rating** = Definitely low (++); Probably low (+); Probably high (specify: - or NR); Definitely high (--). **Bias direction** = Rate when RoB judged as “Probably high” or “Definitely high”.  **Instructions for bias direction rating**: *Upward* = resulting in apparent or increased adverse effects; *Downward* = resulting in reduced/undetected adverse effects, apparent beneficial effects); *Uncertain =* due to missing relevant information and/or lack of ancillary evidence. In the following instances, the bias direction will be easier to anticipate: (a) non-differential and nondependent misclassification of the exposure or the outcome, generally downward (or no bias, in the scenario of a true null association); (b) the healthy worker effect, usually downward; (c) exposure misclassification dependent on the outcome (recall bias), usually upward for self-reported data; (d) confounding variables acting in a predictable direction: based on a simple rule, only valid for dichotomous variables, uncontrolled confounding will result in upward bias when the associations of C with E and D have the same sign and in downward bias when the C-E and C-D associations are in opposite directions. | | | | | | |

Based on reported ambiguities and observed inconsistency in rating, the answer options’ structure was refined, and more effective rating instructions were developed for confounding, selection, and information biases. Improvements to the RoB assessment form to be used during the review were also planned (see § 1.7 below).

Two questions under the “Other bias” domain, characterized by very poor interrater agreement, were deleted.

Q11a concerned the adequacy of range of variation in exposure levels to detect potential exposure-disease association. It was cancelled due to the incomplete coverage of the multiple features affecting the study sensitivity, and for adherence to OHAT’s approach of not considering the study informativeness as a bias element (it will be covered at the stage of evidence appraisal, under indirectness).

Q11b addressed the internal consistency of exposure-response results. It was considered inapplicable to analyses by time since first use by the majority of assessors. When Q11b was applied to the analyses by cumulative exposure in the case-control study, resulted in substantial interrater disagreement, most likely due to lacking clues about the expected shape of the exposure-disease relationship.

1. **Conduct and management of RoB assessment process**

Depending on number of included studies, up to six team members will perform the RoB assessment. The potential for bias of each study and related exposure-outcome contrasts will be rated in duplicate by two assessors. No assessor will evaluate studies that they co-authored. Conflicts will be resolved by consensus, or arbitration by a third member of the review team.

We will use the HAWC platform (Shapiro et al. 2018) to manage the process. The advantages offered by HAWC consists in customizable OHAT-based RoB assessment forms, in-built routine for the assessment of interrater agreement, and predefined visualizations of the RoB assessment results.

The RoB assessment form will provide, for each bias question, separate spaces to record: (a) the endpoint(s) or the exposure-endpoint pair to which the rating applies; (b) the bias rating rationale; (c) the bias direction and relating supporting evidence; (d) potential ambiguities in criteria for assigning the RoB rating for any question.

As foreseen by OHAT, refinements to the RoB rating instructions might be made after the pilot study, or at later stage during the review, due to issues not anticipated during protocol development [(NTP-OHAT 2019), p. 43]. Major changes (e.g., those resulting in revision of response) will be documented with date and justification, and reported in the systematic review paper as changes in the RoB protocol.

# TAILORED RATING INSTRUCTIONS AND ANSWER OPTION FORMS

1. **Confounding**

Observational human studies are prone to confounding bias. Interpretation of study findings may be distorted by failure to consider the extent to which systematic differences in baseline characteristics risk factors, prognostic variables, or co-occurring exposures among comparison groups may reduce or increase the observed effect. Major issues to consider in the assessment of confounding bias are: (a) the appropriateness of methods used to control or adjust for confounding; (b) validity and reliability of methods used to measure confounders [(NTP-OHAT 2015), pp. 11-12].

Confounding is a bias of the estimated effect of an exposure on an outcome due to the presence of a common cause of the exposure and the outcome (Porta 2016). The necessary properties of a confounder are (1) to be associated with exposure in the source population, (2) to be associated with the outcome among the unexposed, and (3) not to be a consequence of either exposure or outcome (Savitz and Wellenius 2016).

The magnitude of confounding bias is a function of the strength of the confounder-disease association (conditional on the exposure) and of the confounder-exposure association in the study population; for discrete confounders, it also depends on the confounder prevalence (Hernán and Robin 2020).

It follows that confounder selection, whatever the strategy (VanderWeele 2019), is a study-specific issue. Therefore, we can only indicate a minimal set of *critical potential confounders* for the exposure-outcome relationships of primary interest for the three components of our systematic review (Table 5).

**Table 5. Critical potential confounders for the exposure-outcome associations relationships of primary interest**

| **Exposure source** | **Outcome** | **Critical potential confounders** |
| --- | --- | --- |
| Wireless phones | Central nervous system tumours and salivary gland tumours | Age, sex, SES, and country/region for multicentre case-control studies. |
| Fixed-site transmitters | Brain cancer or brain tumours and  leukaemias (in adults and children) | Age, sex, time period. |
| Occupational sources | Brain cancer | Age, sex, time period, SES, and co-exposure to IR. |
|  | Leukaemias | Age, sex, time period, SES, tobacco smoking, co-exposures to IR and leukaemogenic chemicals. |
| **SES** = indicators of socioeconomic status (e.g., educational level, household income (general population); salary/income, occupational level, type of employment (workers); **IR** = ionizing radiation. | | |

These variables are common antecedent of the investigated exposure and disease, and not intermediates in the path between the two, very likely to be unequally distributed between study groups (see § III.1 for details).

Studies that have not collected information on such variables, or not controlled them in the design or the analyses, should be considered at high risk of confounding bias. Note that lack of control for socioeconomic status is expected to bias the risk estimates for CNS tumours and leukemias upwards.

For other exposure-neoplasm pairs, potential confounders could not be identified at the stage of protocol development, due to lack of information about the tumour types investigated in the studies that will be included in the important/secondary outcome subsets of the systematic review. Additions to the rating instructions will be made after completing the identification of studies eligible for inclusion.

Beside confounders, Q4 also addresses modifying variables. Effect modification occurs when the magnitude of the investigated exposure-disease association depends on one or more personal characteristics, e.g., age at exposure, sex, genetic traits, ethnicity, socioeconomic status, place of residence, or co-exposures (Miettinen 2011). The great challenge in observational studies is to distinguish apparent effect modifications from consequences of selection or information biases, affecting subsets of the study population unevenly (Savitz and Wellenius 2016). Furthermore, the identification of critical modifiers of the effect of RF-EMF on cancer risk is hampered by the lack of a scientific rationale (usually available for established human carcinogens).

Therefore, the main focus of the tailored Q4 answer options is on confounding bias.

| Q4. Did study design or analysis account for important confounding and modifying variables? |
| --- |
| Cohort, Case-Control (SR-A\|SR-B\|SR-C) |
| Definitely Low Risk of Bias (++) |
| - There is direct evidence that appropriate adjustments or explicit considerations were made for primary covariates and confounders in the final analyses, through the use of statistical models to reduce research-specific bias including standardization, matched analysis, adjustment in multivariate model, stratification, propensity scoring, or other methods that were appropriately justified. Acceptable consideration of appropriate adjustment factors includes cases when the factor is not included in the final adjustment model because the author conducted analyses that indicated it did not need to be included, - **AND** there is direct evidence that primary covariates and confounders were assessed using valid and reliable measurements, - **AND** there is direct evidence that other exposures anticipated to bias results were not present or were appropriately measured and adjusted for. |
| Probably Low Risk of Bias (+) |
| - There is indirect evidence that appropriate adjustments were made, **OR** it is deemed that not considering or only considering a partial list of covariates or confounders in the final analyses would not appreciably bias results, - **AND** there is evidence (direct or indirect) that covariates and confounders considered were assessed using valid and reliable measurements, **OR** it is deemed that the measures used would not appreciably bias results (Note), - **AND** there is evidence (direct or indirect) that other co-exposures anticipated to bias results were not present or were appropriately adjusted for, **OR** it is deemed that co-exposures present would not appreciably bias results (Note). |
| Probably High Risk of Bias (-) or (NR) |
| - There is indirect evidence that the distribution of important covariates and known confounders differed between the groups and was not appropriately adjusted for in the final analyses, - **OR** there is indirect evidence that covariates and confounders considered were assessed using measurements of unknown validity, - **OR** there is indirect evidence that there was an unbalanced provision of additional co-exposures across the primary study groups, which were not appropriately adjusted for, - **OR** there is insufficient information provided about the distribution of known confounders, about the measurement techniques used to assess covariates and confounders considered, or about co-exposures in occupational studies where high exposures to other risk factors, such as chemical exposures or electromagnetic fields of other frequencies, would have been reasonably anticipated (record “NR” as basis for answer). |
| Definitely High Risk of Bias (--) |
| - There is direct evidence that the distribution of important covariates and known confounders differed between the groups, confounding was demonstrated, and was not appropriately adjusted for in the final analyses, - **OR** there is direct evidence that covariates and confounders considered were assessed using non-valid measurements, - **OR** there is direct evidence that there was an unbalanced provision of additional co-exposures across the primary study groups, which were not appropriately adjusted for. |
| **Note:** The authors or the reviewers justified the validity of the measures from previous research, and/or the RoB assessors come to similar conclusion based on currently available evidence. |

1. **Selection biases**

Under the selection bias domain, the OHAT’s RoB tool considers distortions arising at the time of subjects’ recruitment into the study. The question relevant to observational human studies (Q3 –*Did selection of study participants result in the appropriate comparison groups?*) addresses whether exposed and unexposed subjects were recruited from the same populations in cohort or cross-sectional studies, and consideration of appropriate selection of cases and controls in case-control studies [(NTP-OHAT 2015), p 9].

Distortions due to loss of subjects after enrolment or to missing data are considered under the performance bias domain, whereas Q7 addresses incomplete outcome data due to nonresponse, dropout, loss to follow-up, or exclusion from analyses [(NTP-OHAT 2015), p. 19]. The OHAT handbook acknowledges that missing data/attrition/exclusion bias is often referred to as selection bias in observational studies, but notes that recent efforts (Sterne et al. 2014; Viswanathan et al. 2012) encourage consideration of selection and missing data as distinct for observational studies [(NTP-OHAT 2019), Table 4, p. 35].

We encountered two difficulties in customizing these bias questions.

The formulation of Q3 does not allow distinguish selection bias from confounding (see § II.2.1).

The partition of selection biases in two broad categories based on the stage of the study in which the bias occurs has advantages and limitations:

- It fits well to cohort studies, while it is tricky to apply to case-control studies where loss of subjects, due to nonresponse or restrictions on inclusion, mostly occur at enrolment.
- Each category addresses several mechanisms through which selection bias can occur in observational studies. This results in rating a study twice for the same bias, without distinguishing the bias source.
- The relevance of the two questions is contingent on the study design, because each one addresses the main sources of selection bias in cohort studies (Q7) or in case-control studies (Q3).

The RoB tool developed by the Office of the Report on Carcinogens overcomes these problems, focusing on a single bias domain (“Selection and attrition bias”), with one core question (“Is there concern that selection into the study or out of the study was related to both exposure and to outcome?”), and design-specific signalling and follow-up questions [(NTP-ORoC 2015) pp. 28-31, Table D-2]. For consilience with the OHAT’s approach, we did not merge the selection and attrition bias questions, and developed alternative strategies to reduce the aforementioned drawbacks (see § II.2.2).

- 1. *Structure and mechanisms of selection bias*

Although lack of exchangeability between exposed and unexposed is a shared feature of confounding and selection bias, these biases have different structures (Hernán et al. 2004).

Confounding is due to a common cause of the disease and exposure, and uncontrolled confounding is expected to induce an exposure-disease association on all scales (risk ratio, risk difference, etc.), regardless of whether the exposure does or does not have an effect on the outcome.

Selection bias is due to an effect of the exposure and/or the disease on the probability of inclusion and retention in the analytic sample. The conditions for selection bias to occur depend on the underlying scenario (true null or non-null effect of the exposure). The scale of effect measure is relevant in the off null scenario.

Assuming no effect of the exposure on the outcome (*under the null*), selection bias arises from conditioning on the common effect of two variables, one of which is either the exposure or associated with the exposure, and the other is either the outcome or associated with the outcome (Hernán and Robin 2020). Due to *collider stratification bias*, a measure of association (whatever its scale) is a biased estimate of the effect of the exposure in both the target population and the analytical sample (Hernán 2017).

When the exposure has an effect on the outcome (*off the null*), selection bias may also occur when selection/censoring is not a collider but it is associated with a predictor of the disease. In this case, the association measure *may* be biased for the effect in the entire population, depending on the effect measure’s scale (Greenland 1977; Howe et al. 2016). The causal risk difference will be inaccurately estimated. However, for a bias to occur on the risk-ratio scale, an additional condition is required; the association between selection/attrition and the disease predictor must vary across exposure levels (i.e., there must be an interaction between exposure and susceptibility to the disease). If there is no heterogeneity of disease risk between censored and uncensored, there is no selection bias for the population risk ratio.

In summary, conditioning on a collider will always compromise the validity of the study findings, while conditioning on a noncollider may alter an existing exposure-disease association, preventing the generalizability of the measures of effect (Hernán 2017).

Selection bias can occur (a) at entry in the study, if recruitment or participation are simultaneously determined by the exposure and the disease, or by a cause of the exposure and the risk profile for the disease; (b) between entry and analysis, from differential informative censoring; (c) at the analysis stage: in regression analyses, by conditioning on time-dependent confounders that have also become colliders; in the development of the propensity score with conditioning on what is thought to be a pre-exposure confounder but is a collider; or in mediation analysis, by conditioning on a mediator that is also a collider (Infante-Rivard and Cusson 2018).

The direction of selection bias can be anticipated: usually, if the exposure and the outcome variables have an effect on the selection indicator (S) in same direction (either positive or negative), their spurious association will be negative; it will be positive if their effect on S is in opposite directions (Infante-Rivard and Cusson 2018).

The identification of the likely sources and mechanisms of selection bias is informed by issues relevant to the specific exposure-outcome relationship of interest.

An increased cancer risk at the population level usually becomes detectable only after a minimum latency period, and/or above a threshold level of exposure. The relevant exposure time-window is expected to be neoplasm-specific, and for specific tumours may vary according to individual characteristics (e.g., age at exposure).

RF-EMF might promote the growth of existing tumours and/or induce cancer. Considerations about the exposure source are also relevant. For example, the level of exposure to RF-EMF from mobile phone calls is a time-dependent variable, due to the decreasing trend in average transmission per call over calendar time, possibly modified (at least partially compensated) by an increasing amount of use over time. Selection bias might occur if inclusion in one or more of the exposure stratum of the analytical sample (enrolled and uncensored) depended jointly on the exposure at baseline and the risk profile for the index disease (including a different prevalence of unmeasured/not properly controlled predictors of disease risk across exposure strata).

Proneness to selection bias and mode of occurrence of this bias differ between cohort and case-control studies.

Cohort studies often deliberately select special populations; however, no selection bias will result if selection/participation/inclusion in the study or the analysis is not affected by correlates of disease risk (Checkoway et al. 1989; Savitz and Wellenius 2016).

The main mechanisms of selection bias in cohort studies is from loss to follow-up (or other reasons of missing outcome data) that is differential by exposure and disease status. Therefore, the key question is whether there is a preferential loss of diseased individuals that differs for the exposed and unexposed.

In general, selection bias is of low concern for cohort studies based on prospective recording of exposure (i.e., assessed before and independent of outcome occurrence/diagnosis/ascertainment), assessment of the exposure during the whole relevant time-window, and record-linkage to population-based registers for complete follow-up of cancer occurrence (Feychting 2014; Pinsky et al. 2016).

Assuming effective control of confounding and no exposure misclassification, selection bias (under the null) is of concern for cohort studies with incomplete enrolment of eligible exposed (and/or differential attrition), and recruitment (and/or missing data) possibly associated with the risk profile for the index neoplasms through factors other than the measured confounders adjusted for in the analyses. Restriction to subjects free of the index diseases at entry, and left-censoring of exposure (with lags accounting for the tumour-specific induction-latency period), would lessen concern for collider stratification bias.

Case-control studies are inherently susceptible to selection bias, because the association measure (the exposure-outcome odds ratio) is by definition conditional on having been selected into the study (Hernán and Robin 2020).

The major sources of selection bias in case-control studies are outlined below.

- Selection bias arises when cases and controls do not come from the same source population. The key concern is not a comparison between cases and controls, but a comparison between the controls and the source population that they are intended to represent. Moreover, case-control studies enrolling cases diagnosed over a period of time that includes the past, and investigating the effect of an exposure that changes over-time, are prone to selection bias from lack of temporal coherence between the source populations of cases and controls (Savitz and Wellenius 2016).
- Participation dependent on both the exposure and the disease (or their correlates) is another source of selection bias in case-control studies. Participation rates are not always correctly calculated and adequately reported. They must be calculated, separately for cases and controls, as the number of participants divided by the number of all eligible subjects (including those not contacted due to death, ill health or physician denying permission, the untraced, and those refusing participation), and reasons of nonresponse have to be reported in detail for both study groups (Elwood 2017).

The magnitude and direction of bias from differential participation depends on the selection bias factor [(P_11_/P_01_)/(P_10_/P_00_) = (P_11*_P_00_)/(P_10*_P_01_], that is the ratio of participation probabilities of exposed (P_11_) and unexposed (P_01_) cases to that of exposed (P_10_) and unexposed (P_00_) controls (Greenland and Lash 2012).

The information required to estimate the selection bias factor (i.e., the exposure prevalence among non-participant cases and controls) is rarely available. It is worth reminding, however, that low response rates (among cases and/or control) do not necessarily result in selection bias, and equal response rates in cases and controls offer no protection if the relevant correlates of nonparticipation are different in the sick and the healthy (Feychting 2014; Hartge 2006). Therefore, we are reluctant to provide numerical guidance to rating, in terms of either absolute or relative optimal/critical participation rates among cases and controls.

- Restrictions on inclusion (in the study or the analyses) based on health/vital status, can also result in selection bias if the exposure probability/duration/amount differ between included and excluded subjects.
- Selection bias may also arise at the analysis stage. Individually matched case-control studies should be analysed using conditional logistic regression. If matching is ignored, the matching variables included in the unconditional logistic regression model must be categorized in strata finer than the original matching criteria (e.g., 1-year age strata for a 5-year matching) in order to remove the selection bias introduced by matching (Greenland 2012). The sex variable obviously escapes this remedy. In a case-control study of CNS tumours (any subtype) and mobile phone use, with controls individually matched to cases on sex and age, most controls will be females, with a shorter duration of mobile phone use compared to males of similar age at diagnosis. That occurs because the most common subtype of CNS tumours is meningioma, whose incidence rates are over 3 times higher in females than males aged ≥35 years (Ostrom et al. 2020), and men started to use mobile phones earlier than women (SCENIHR 2009). If the whole series of controls is used in unconditional regression analyses restricted to cases of CNS subtypes with a m/f ratio ≥1 (such as glioma or acoustic neuroma), adjustment for sex might not offset the upward selection bias introduced by matching.
  1. *Customized approach to the assessment of selection and attrition biases*

Our tailored Q3 answers options for cohort studies avoid mixing up issues related to selection bias and to confounding. Differences in baseline characteristics between exposed or unexposed (or across exposure groups) are examined as indirect evidence of probably low/high risk of selection bias, without losing sight of the fundamental requirement for selection bias to occur.

In line with OHAT’s indications, we also consider two issues specific of occupational cohort studies, currently interpreted as a form of confounding by health status (Green-McKenzie 2017; Naimi et al. 2013):

- The selection of healthy workers into the workplace (healthy-worker hire effect, HWHE), of concern in occupational cohort studies using the general population as the reference group (i.e., reporting standardized incidence or mortality rate ratios as measures of effect);
- The selection of unhealthy workers out of the workplace (healthy worker survival effect, HWSE), a form of time-varying confounding, which may be an issue in cohort studies enrolling prevalent (as opposed to incident) exposed workers, regardless of the type of reference group.

However, the potentials for HWHE/HWSE and selection bias will be assessed separately.

For case-control studies, we address in Q3 issues related to the study base and to subjects’ participation.

The Q7 answer options, for both cohort and case-control studies, cover lack of any information required for inclusion in the analyses (i.e., missing data about the outcome, exposure details, confounders, and other covariates). For case-control studies, exclusions of subsets of cases based on severity of the disease or vital status at recruitment are addressed in Q7. This will only partially mitigate the predominant relevance of this question to the assessment of the potential for selection bias in cohort studies.

Therefore, to ensure coherence in assessing the potential for selection bias across study design, we will combine the Q3 and Q7 ratings. To this aim, we will adopt criteria similar to those envisaged by ROBIN-I to arrive at an overall risk-of-bias judgment (Sterne et al. 2019). The default rule will be a combined rating corresponding to the less favourable of the two ratings for selection bias (excluding judgments relating to HWHE and HWSE). This will always apply when any of the two ratings is “definitely high risk of bias”. Deviations are admissible for other combinations, depending on the rating values and the anticipated implications for the study results (i.e., accounting for the anticipated bias magnitude, as described in § I.3). When one rating is definitely or probably low risk, and the other probably high risk of bias, the more favourable rating may be chosen if the expected magnitude of bias from the source of greater concern is modest. When both ratings are probably high risk, a combined judgment of definitely high risk may be considered when the anticipated magnitude of bias from any of the two sources is substantial.

| Q3. Did selection of study participants result in the appropriate comparison groups? |
| --- |
| Cohort (SR-A\|SR-B\|SR-C) |
| Definitely Low Risk of Bias (++) |
| - There is direct evidence (Note 1) that exposed and non-exposed subjects (or subgroups by exposure level) were recruited from the same eligible population within the same time frame, with the same method of ascertainment, using the same inclusion and exclusion criteria, - **AND** probability of enrolment/participation did not depend on *both* exposure status and risk profile for the outcome(s) of interest. |
| Probably Low Risk of Bias (+) |
| - There is indirect evidence (Note 2) that exposed and non-exposed subjects (or subgroups by exposure level) were recruited from the same eligible population within the same time frame, with the same method of ascertainment, using the same inclusion and exclusion criteria, - **AND** probability of enrolment/participation did not depend on *both* exposure status and risk profile for the outcome(s) of interest, - **OR** differences between groups would not appreciably bias results. |
| Probably High Risk of Bias (-) or (NR) |
| - There is indirect evidence (Note 3) that exposed and non-exposed subjects (or subgroups by exposure level) were not recruited from the same eligible population, or were recruited within very different time frames, - **AND** probability of enrolment/participation depended on both exposure status and risk profile for the outcome of interest (selection bias), - **OR** there is insufficient information about the unexposed/comparison group, including a different rate of non-response without an explanation (record “NR” as basis for answer). |
| Definitely High Risk of Bias (--) |
| - There is direct evidence (Note 4) that exposed and non-exposed subjects (or subgroups by exposure level) were not recruited from the same eligible population, or were recruited within very different time frames, - **AND** probability of enrolment/participation depended on both exposure status and risk profile for the outcome(s) of interest (selection bias). |
| **Instruction reminder and notes**  Focus on whether enrolment/participation in the study is jointly affected by the exposure and the risk profile for the disease of interest. For studies investigating risk of multiple neoplasms, provide endpoint-specific ratings. For occupational studies, rate separately the potentials for selection bias, HWHE and HWSE.  **Note 1.** Consider compliance with all features below as direct evidence of low risk of selection bias:   - The cohort is clearly defined in space and time (it includes the exposed and non-exposed for a specific time period/location), AND inclusion is restricted to persons free of the index disease(s) at baseline; - All new exposed have been identified, OR inclusion was limited to a subset of incident exposed but predictors of the disease (others than the exposure) were controlled in the analyses, OR enrolment was voluntary and based on prevalent exposure at baseline, but past exposure histories were assessed at baseline; AND exposure assessment covers the whole time-window relevant for the index tumour(s); - Analyses are based on internal comparison.   **Note 2**. Examples of *indirect evidence* of low risk of selection bias include:   - - - Descriptive analyses showing that participants do not differ from eligible nonparticipants with respect to key-variables (e.g., age, sex, indicators of socio-economic status); prevalence and levels of exposure in the analytical sample are similar to those expected in the eligible population; rates of the disease(s) of interest in the unexposed portion of the cohort are similar to those expected in the eligible population.     - Sensitivity analyses (e.g., analyses restricted to subsets of the cohort less affected by the postulated source of selection bias), providing results in line with the main analyses.   **Note 3**. Examples of *indirect evidence* of high risk of selection bias include:   - - - Descriptive analyses showing that participants differ from eligible nonparticipants with respect to key-variables (e.g., age, sex, indicators of socio-economic status); prevalence and levels of exposure in analytical sample differ from those expected in the eligible population; rates of the diseases of interest in the unexposed portion of the cohort differ from those expected in the eligible population;     - Sensitivity analyses (e.g., analyses restricted to subsets of the cohort less affected by the postulated source of selection bias), providing results not in line with the main analyses.   **Note 4**. Consider departure from all features in Note 1 as *direct evidence* of high risk of selection bias. |

| Q3. Did selection of study participants result in the appropriate comparison groups? |
| --- |
| Case-Control (SR-A\|SR-B\|SR-C) |
| Definitely Low Risk of Bias (++) |
| - There is direct evidence (Note 1) that cases and controls were recruited from the same eligible population within the same time frame and continuously throughout the study period, using similar eligibility criteria (e.g., age, gender, ethnicity) other than the outcome of interest, and analogous enrolment strategies (tracing/contact), - **AND** probability of participation is unlikely to be associated with the exposure and the case/control status. |
| Probably Low Risk of Bias (+) |
| - There is indirect evidence (Note 2) that cases and controls were recruited from the same eligible population, within the same time frame and continuously throughout the study period, using similar eligibility criteria (e.g., age, gender, ethnicity) other than the outcome of interest, and analogous enrolment strategies (tracing/contact), - **AND** probability of participation is unlikely to be associated with the exposure and the case/control status. |
| Probably High Risk of Bias (-) or (NR) |
| - There is indirect evidence (Note 3) that controls were drawn from a very dissimilar population than cases or recruited within very different time frames, - **OR** probability of participation likely to be associated with the exposure, - **OR** there is insufficient information provided about either the appropriateness of controls, or participation in the study, including participation rates and/or distribution of non-participants by reason of nonresponse available for cases only (record “NR” as basis for answer). |
| Definitely High Risk of Bias (--) |
| - There is direct evidence (Note 4) that controls were drawn from a very dissimilar population than cases, or that were recruited within very different time frames, or that probability of participation depended on both the exposure and the case/control status. |
| **Instruction reminder and notes**  For studies investigating risk of multiple neoplasms, provide endpoint-specific ratings. Remember that exclusions of eligible subjects at recruitment or from the analyses is addressed in Q7 (not herein).  **Note** **1**: Compliance with all the design features and participation requirements indicated below will be considered *direct evidence* of low risk of selection bias:   - A source population (study base) clearly defined in space and time, either completely enumerated (i.e., case-control studies nested in a cohort), or fully identifiable (i.e., population-based case-control studies conducted in countries/regions with access to dynamic population registers for control selection); - Prospective and complete ascertainment of all newly diagnosed (incident) cases of the disease (as defined) occurring in the source population during the entire study period, through appropriate sources, such as good quality population-based cancer registries, or large hospital networks with an overall catchment area coincident with the study area; - Selection of controls from population based rosters and through sampling methods appropriate to obtain an unbiased sample of the source population-time experience (i.e., controls selected from those who would have become identified as cases in the study had they developed the disease of interest, and representative of the exposure distribution in the study base during the study time-frame), such as the dynamic population registries available in several European countries (either at the municipal/regional level, or nation-wide in the Nordic Countries), electoral rolls, or general practitioner lists (in countries with universal-coverage health systems)]; - Equivalent ratios of exposed to unexposed participation rates among cases and among controls. - Statistical models appropriate for the study design (e.g., conditional logistic regression for individually matched case-control studies).   **Note** **2**: Examples of *indirect evidence* of low risk of selection bias include:   - Comparison of exposure prevalence and pattern (e.g., distribution by calendar-time, and subjects’ demographics) measured among controls to an external population, showing correspondence between observed and expected values; - Analyses of the exposure-disease association stratified on markers of susceptibility to selection bias [e.g., by severity of the disease, presence/absence of specific symptoms, time-period of diagnosis, accessibility of health care based on geography or financial constraints, or type of control disease (the latter two limited to hospital-based case-control studies)], showing similarity of findings across strata; - Bias studies showing a *modest* impact of selection bias on the measure of effects, with modest meaning that the estimated bias factor cannot explain out the observed association or it cannot change a null association into a positive/negative one.   **Note** **3**: Examples of *indirect evidence* of high risk of selection bias include:   - Comparison of exposure prevalence and pattern (e.g., distribution by calendar-time, and subjects’ demographics) measured among controls to an external population, showing lack of correspondence between observed and expected values; - Analyses of the exposure-disease association stratified on markers of susceptibility to selection bias [e.g., by severity of the disease, presence/absence of specific symptoms, time-period of diagnosis, accessibility of health care based on geography or financial constraints, or type of control disease (the latter limited to hospital-based case-control studies)], showing dissimilarity of findings across strata; - Bias studies showing a *substantial* impact of selection bias on the measure of effects, with substantial meaning that the estimated bias factor can explain out the observed association or it can change a null association into a positive/negative one.   **Note 4**: Consider as *direct evidence* of high risk of selection bias: departure from all design features in Note 1. |

| **Q7. Were outcome data complete without attrition or exclusion from analysis?** |
| --- |
| **Cohort (SR-A\|SR-B\|SR-C)** |
| Definitely Low Risk of Bias (++) |
| - There is direct evidence that loss of subjects (i.e., incomplete outcome data) was adequately addressed (Note 1) and reasons were documented when human subjects were removed from a study. - **OR** missing data (on exposure details, confounders, or covariates) have been imputed using appropriate methods, and characteristics of subjects lost to follow-up or with unavailable records are described in identical way and are not significantly different from those of the study participants (or included in the analyses). |
| Probably Low Risk of Bias (+) |
| - There is indirect evidence that loss of subjects (i.e., incomplete outcome data) was adequately addressed (Note 1) and reasons were documented when human subjects were removed from a study, - **OR** it is deemed that the proportion lost to follow-up, or excluded from the analyses due to missing data, would not appreciably bias results (Note 2). |
| Probably High Risk of Bias (-) or (NR) |
| - There is indirect evidence that loss of subjects (i.e., incomplete outcome data, or missing information required for inclusion in the analyses) was unacceptably large (>20 %) and not adequately addressed, - **OR** there is insufficient information provided about numbers of subjects lost to follow-up (record “NR” as basis for answer). |
| Definitely High Risk of Bias (--) |
| - There is direct evidence that loss of subjects (i.e., incomplete outcome data, or missing information required for inclusion in the analyses) was unacceptably large (>20 %) and not adequately addressed (Note 3). |
| **Instruction reminder and notes**  For studies investigating risk of multiple neoplasms, provide endpoint-specific ratings.  **Note 1**: Acceptable handling of subject attrition includes: very little (≤20%) missing outcome data; reasons for missing subjects unlikely to be related to both outcome and exposure (for survival data, censoring unlikely to be introducing bias); balanced proportions of missing data across study groups, with similar reasons for missing data across groups.  **Note 2:** This would include reports of no statistical differences in characteristics of subjects lost to follow up or with unavailable records from those of the study participants, or included in the analyses. Generally, the higher the ratio of participants with missing data to participants with events, the greater potential there is for bias. For studies with a long duration of follow-up, some withdrawals are inevitable.  **Note 3**: Unacceptable handling of subject attrition includes: reason for missing outcome data (or missing information required for inclusion in the analyses) likely to be related to true outcome and exposure, with either imbalance in proportions or reasons for missing data across study groups, or potentially inappropriate application of imputation. |

| **Q7. Were outcome data complete without attrition or exclusion from analysis?** |
| --- |
| **Case-Control (SR-A\|SR-B\|SR-C)** |
| Definitely Low Risk of Bias (++) |
| - There is direct evidence that exclusion of subjects from the study/analysis was adequately addressed and reasons for exclusion were documented (Note 1). |
| Probably Low Risk of Bias (+) |
| - There is indirect evidence that exclusion of subjects from the study/analysis was adequately addressed and reasons for exclusion were documented (Note 1), - **OR** it is deemed that the proportions of excluded cases/controls would not appreciably bias results (Note 2). |
| Probably High Risk of Bias (-) or (NR) |
| - There is indirect evidence that exclusion of subjects from the analyses was not adequately addressed, - **OR** there is insufficient information provided about why subjects were removed from the study or excluded from analyses (record “NR” as basis for answer). |
| Definitely High Risk of Bias (--) |
| - There is direct evidence that exclusion of subjects from analyses was not adequately addressed (Note 3). |
| **Instruction reminder and notes**  For studies investigating risk of multiple neoplasms, provide endpoint-specific ratings if appropriate.  **Note 1**: Acceptable handling of subject exclusion include: reasons for exclusion unlikely to be related to both outcome and exposure; balanced proportions of missing data (on exposure details, confounders, or covariates required for inclusion in the analyses) in the case and control groups, with similar reasons for missing data across groups.  **Note 2**: This would include reports of no statistical differences in characteristics of cases and controls excluded from the analyses from those of included cases and controls.  **Note 3**: Unacceptable handling of subject exclusion includes: reasons for exclusion likely to be related to both outcome and exposure, with either imbalance in proportions or reasons for missing data across study groups. |

1. **Information biases**

Under the “Detection bias” domain, the OHAT’s RoB tool addresses systematic differences between study groups with regards to how outcomes and exposures are assessed and also considers validity and reliability of methods used to assess outcomes and exposures [(NTP-OHAT 2015), p. 22].

For this bias domain we prefer using the label “Information” bias, because epidemiologists regard detection bias as a specific type of outcome misclassification (see § II.5.1).

Observational studies try to estimate the effect of exposure on the outcome, using imperfectly measured variables. The exposure and outcome indicators are susceptible to four types of misclassification or measurement error, with reference to binary or continuous variables respectively (Hernán and Robin 2020):

1. *independent non-differential* (syn. random);
2. *dependent non-differential*: systematic errors whose extent varies with the actual value of the parameter;
3. *independent differential*: random errors whose amount varies between diseased and non-diseased (for exposure variables), or across exposure levels (for outcome indicators);
4. *dependent differential*: systematic errors whose amount varies across outcome and exposure groups.

Random misclassification/mismeasurement usually bias the study results towards the null. Noteworthy, if the exposure had no effect on the outcome (under the null), random errors result in loss of precision with no bias.

Systematic and differential misclassification/mismeasurement are sources of bias away from the null.

The timing of the exposure and outcome assessments affects the potential for different types of information errors (differential *vs* non-differential), as well as the amount of non-differential errors in self-reported variables. Susceptibility to differential misclassification of the exposure or the outcome may vary across tumours and types of RF exposure. On equals term, each operational variable has its own validity (sensitivity and specificity). Therefore, information bias must be assessed:

- taking into account the study design;
- at the level of specific exposure-outcome pairs;
- separately for distinct exposure/outcome indicators.

1. **Confidence in exposure characterization**

Confidence in the exposure requires valid, reliable, and sensitive methods to measure exposure applied consistently (i.e., under the same method and time-frame) across groups. Exposure is much more difficult to measure accurately for observational studies than for controlled exposure studies. Therefore, exposure measurement error and misclassification are more likely to contribute to risk of bias for observational studies. Acceptable methods for measuring exposure will be highly exposure dependent and therefore a specific list of acceptable, inaccurate, or potentially biased methods should be developed for each evaluation and will require subject-matter expertise [Excerpts from (NTP-OHAT 2015), pp. 22-23].

Critical issues in the assessment of exposure to RF-EMF in epidemiological studies, in general and with reference to specific exposure sources, are summarized in § 1.2 and § 3.2.2 of current review protocol. Additional topics relevant to the assessment of exposure-information bias are discussed below.

- 1. *Types of exposure misclassification and study design*

The potential for different types of exposure misclassification depends on whether the exposure is assessed prospectively (i.e., before and independently of the outcome) or retrospectively (after the outcome occurrence/diagnosis/ascertainment), which is a feature of the study design.

Non-differential exposure misclassification can affect studies of any design. That notwithstanding, as the memory of past events becomes less precise over time, self-reported exposure variables (e.g., date of start exposure) are expected to be less affected by random misclassification in cohort than in case-control studies, due to the shorter delay between the event and the interview in the former compared to the latter.

Case-control studies with retrospective exposure assessment based on self-reports, are distinctively susceptible to recall bias *(*see definition below). Cohort studies with prospective exposure assessment (as well as case-control analyses of cohort studies with the aforementioned features) are immune to recall bias, regardless the source of exposure data (independent records or self-reports), and no matter whether the information refers to current or past exposures. The same may apply to case-control studies relying on exposure data from independent sources, recorded before the disease occurrence/diagnosis/ascertainment, provided that data collection and availability are independent of the exposure and case-control status. Note the relevance of the latter condition.

- 1. *Recall bias, mode of self-reported data elicitation and interview timing*

The term *recall bias* is used with reference to differential measurement errors in self-reported exposure data collected after occurrence of the outcome (Savitz and Wellenius 2016).

It occurs when cases and controls do not remember/report previous events or experiences with the same accuracy or omit details at a different extent, and whenever the accuracy and volume of memories is influenced by the presence/absence of the disease (Catalogue of Bias Collaboration et al. 2017).

Bias in recall can be greater when events over a longer time interval are being asked about, and may increase with increasing time before the interview (Vrijheid et al. 2009).

Case-control studies of topics attracting considerable media attention are especially prone to recall bias. For example, in late 1990’s a paper reporting a postulated causal association between the measles/mumps/rubella (MMR) vaccine and autism (based on fraudulent data and subsequently retracted) was extensively publicized. Parents of autistic children diagnosed after the publicity tended to recall the start of autism soon after the MMR injection more often than parents of autistic children diagnosed prior to the publicity (Andrews et al. 2002).

Strategies to reduce the potential for recall bias in case-control studies include choosing an appropriate data collection methods, a careful selection of the research questions, and studying people with new-onset disease (Catalogue of Bias Collaboration et al. 2017).

- In-person interviews are preferred over phone interviews, and information obtained from the subject is preferred over information from proxy respondents (Hutter et al. 2012; NTP-ORoC 2015).
- Exposure-assessment investigators and interviewers should be blinded to the disease status of the study participants. Of these, the blinding of the investigators conducting exposure classification is considered the most important; blinding of in-person interviewers may not be feasible in case-control studies of subjects with cancer (NTP-ORoC 2015).
- Mailed questionnaires are particularly prone to recall bias from lack of blinding on health status, as no one is less blinded to the outcome than the subjects themselves (Bowling 2005).
- The questionnaire/interview format can affect the accuracy of self-reported data:
  - Providing categories instead of open questions to collect continuous data, is expected to improve data accuracy and remove extreme values (Boase and Ling 2013; Mireku et al. 2018).
  - For time-varying exposures, enquiring about changes in exposure frequency/intensity over time allows obtaining more reliable estimates of cumulative exposure, compared to tools eliciting information on the exposure level at a single/few point(s) in time (Pettersson et al. 2014).
- Discrepancies in interview timing across groups is another issue relevant for time-varying exposures: the larger the case-control differential in delay between the diagnosis/reference date and the interview, the higher the potential for recall bias.

In individually matched case-control studies, controls are often interviewed later than cases. In a situation where the exposure prevalence is rapidly increasing during the recruitment period, and/or the intensity of exposure at the time of interview influences recall of previous exposures, a longer delay in interview timing could explain an apparently greater amount of exposure reported by controls compared to cases. The occurrence of this differential exposure misclassification (potentially resulting in a downward bias), was assessed on a subset of data from the Interphone study, using a post-hoc matching process; there was some evidence of bias in the expected direction, although results were generally similar to findings from the original analyses (Turner et al. 2016).

In the same situation, if controls were selected/contacted/interviewed systematically before cases, the differential exposure error would result in an upward bias.

A short delay between diagnosis and interview is particularly important for studies of central nervous system tumours (see § II.5.2).

- 1. *Reverse causation*

In terms of structure, reverse causation is a form of confounding from the disease itself (Hernán and Robin 2020). It is a major issue in cross sectional studies, and can also arise in longitudinal studies investigating risk factors for long-latency diseases such as cancer.

It is addressed in Q8 because, especially in case-control studies with retrospective exposure assessment based on self-reports, reverse causation can result in differential exposure misclassification dependent on the outcome (Hernan and Robin 2020).

Reverse causation may also affect findings from cohort studies. For example, improvements in exposure assessment and statistical methods are being studied to clarify how much of the excess risk of brain cancer after CT scans in children is attributable to reverse causation rather than to the causal effect of ionizing radiation (Brady et al. 2020; Smoll et al. 2020).

In studies of mobile phone use and CNS tumours, pre-clinical or early symptoms of the disease can make cases less likely to take up regular mobile phone use close to the time of diagnosis, producing spurious inverse or reduced exposure-disease association, especially in the lowest categories of time since start exposure (Olsson et al. 2019; Schüz et al. 2009).

- 1. *Validity issues in the assessment of RF exposure from wireless phone use*

The exposure of interest for tumours in the head region consists of the RF-EMF energy emitted by handheld mobile or cordless phones during voice calls, with the device held next to the head. For use of any wireless phone, we will focus on the following exposure variables: ever *vs* never (regular) usage, time since start use, cumulative hours of use, and cumulative number of calls.

Using subscriber status as proxy for mobile phone use is prone to random exposure misclassification due to users not subscribing for a mobile phone and subscribers not using their phone (Schüz and Johansen 2007). The issue is also relevant to traffic data as indicators of amount of phone, or used as “gold standard” to validate self-reported data. Proneness of operators’ data to random exposure misclassification was likely less pronounced in early studies, when mobile phone use was very expensive and it was unlikely that parents would get a subscription for their children.

Self-reported mobile phone use is susceptible to random exposure error and, in studies based on retrospective exposure assessment, also to systematic and differential errors.

In examining findings from analyses by time since start of mobile phone use (*syn*. since first use), consider that:

- Subscription dates provided by network operators are less likely to be affected by random misclassification compared to self-reported information on date of start use; when collected prospectively (before and independently of outcome ascertainment), both exposure indicators are free from recall bias.
- In case-control studies relying on interview/questionnaire data, the variable time since first use is likely to be less affected by recall bias than indicators of cumulative exposure, because it is based on a self-reported event occurring once. No statistically significant case-control differences in accuracy of reported calendar year of start mobile phone use were detected in validation studies carried out among adults (Pettersson et al. 2015) or children (Aydin et al. 2011), although differential errors cannot be excluded (see next two items).
- A higher prevalence of cases than controls reporting start dates preceding the availability of handheld mobile phones in the study area (i.e., 1984 in the Unites States; 1987 in the Nordic Countries) was observed in several case-control studies [see for example (Hardell et al. 2013)]. Therefore, in evaluating case-control studies relying on self-reports, check the plausibility of the upper values of time since start/length of mobile phone use, and compare the frequency of implausible values between exposed cases and controls.
- Both cohort and case-control studies of CNS tumours (at variable extent, depending on the neoplasm) are susceptible to reverse causation. Left-censoring of exposure histories, using neoplasm-specific lags (i.e., appropriate for the assumed length of the latency period), may help reducing temporal bias.

In examining findings from analyses by amount of mobile phone use (cumulative call time, total number of call), consider the issues below.

- These analyses are currently available from case-control studies only. Cumulative call time was usually calculated multiplying self-reported number of calls, average call duration, and total period of use (accounting for use of hand-free kits when this information was available).
- Some studies collected separate information on amount of use per period of homogenous usage pattern, and the cumulative exposure indices were obtained summing up the period-specific amounts of use. In other studies, information on variations in frequency and duration of calls over time was not sought. Check the structure of the questionnaire/interview used to collect mobile phone histories: the cumulative exposure indices in the latter studies are likely to be less accurate than in the former, because past intensity of use is more difficult to recall than current use, as mobile phone use habits have changed considerably over time.
- The accuracy of self-reported amount of mobile phone use has been assessed using traffic data or software modified phones (able to record the phone output power and, more recently, the side of head in contact with the device). These validation studies have shown that recall of past use of mobile phone is affected by substantial random error, and that amount and laterality of use are susceptible to systematic error.
- Most often, number of calls were reported more accurately than call duration, and participants underestimated the number of calls, while overestimated call duration (Goedhart et al. 2015; Goedhart et al. 2018; Kiyohara et al. 2018; Mireku et al. 2018; Vrijheid et al. 2006). However, overestimation of both number and duration of call was observed in other studies (Aydin et al. 2011; Heinavaara et al. 2011; Kobayashi and Boase 2012), and a tendency for greater underestimation than overestimation of both variables was also reported (Toledano et al. 2018).
- Geometric mean ratios of self-reported *vs* recorded amount of use increased with level of reported use (i.e., evidence of systematic error), with high mobile phone users overestimating their use, and low phone users underestimating it (Goedhart et al. 2015; Goedhart et al. 2018; Vrijheid et al. 2006).
- Reporting error (both random and systematic) also increased with elapsed time between use and interview (Aydin et al. 2011; Kiyohara et al. 2018; Vrijheid et al. 2009).
- Differential errors in self-reported use (recall bias) have been assessed in a few studies. Brain tumour cases tended to over-report amount of mobile phone use more strongly than controls, and the extent of recall bias increased with increasing time before the interview (Vrijheid et al. 2009). People with symptoms were more likely to overestimate call duration compared to asymptomatic cohort members (Toledano et al. 2018).
- Note that recall bias may also occur in studies with objective traffic data, if more cases than controls provide the required consent and phone number [see (Aydin et al. 2011; Pettersson et al. 2015; Vrijheid et al. 2009)].
- First generation (1G) phones had constant average output power of 900 mW (NMT450) or 600 mW (NMT900, ETACS, TACS, NET-C, AMPS, NTT) (AGNIR 2012). The average transmission power of later generation phones in real conditions of use depends on service. Current knowledge would suggest that the phone average output power per call has been decreasing over the last two decades. Drawing upon available estimates (Joshi et al. 2020; Lauer et al. 2013; Persson et al. 2012; van Wel et al. 2021), a user of a 3G/4G phone would have to achieve a call time 50 to 150 times as long as a 2G phone user to get the same accumulated energy deposition. However, old and new networks coexist for some time, new generation devices can usually connect to previous generation base stations, and stopping use of mobile phone is uncommon.
- Operator data are indispensable to calculate the fraction of call time per mobile phone standard (Auvinen et al. 2019). Lack of these data (as it is usually the case for the studies on neoplasms conducted to date), prevents developing exposure indicators that properly account for this determinant of the exposure level.
- The phone make/model or the date of start use provide limited indications about the expected gradient of exposure level by standard (in descending order from 1G to 3G/4G). Multiple counting of individual data is an issue in subgroup analyses based on device features. Analyses by amount of use stratified by recency of start use may be informative for cohort studies with prospective exposure assessment, but less so for case-control studies, where proneness to information bias would hamper the interpretation of results. Remind that a higher prevalence of cases than controls reporting start dates preceding the introduction of handheld mobile phones was observed in several case-control [see for example (Hardell et al. 2013)].

If cancer incidence increased with increasing total absorbed RF energy, and the decreasing average exposure per call over time is compensated/offset by a time-related increase in frequency and duration of calls, the analyses by time since start use would be less informative (i.e., affected by random exposure misclassification to a greater extent, assuming absence of bias and confounding) than analyses by cumulative call time.

- National survey data may reduce this concern. In Denmark, early subscription holders (1987-1995) were heavier users than later subscription holders (1996-2002), with weekly average length of outgoing calls of 23 vs 17 minutes, respectively (National IT and Telecom Agency 2001).

Self-reported use of cordless phones (length or amount) is impossible to validate, due to lack of objective data.

- The average output power of DECT cordless phones is 10 mW circa (SCENIHR 2015), 1-2 orders of magnitude lower than that of 1G-2G mobile phones (Lauer et al. 2013). Check the internal coherence of findings from studies investigating the effect of mobile and cordless phones.
  1. *Validity issues in the assessment of RF exposure from transmitters*

Epidemiological studies of cancer risk in relation to far-field RF exposure from transmitters have usually relied on measured or modelled levels of electric or magnetic fields or power density (at the subjects’ residence, and less often also at schools), or on crude exposure proxies such as distance to the exposure source. Objectively recorded (e.g., derived from geocodes) distance to the source may be informative for antennas with a roughly isotropic transmission pattern. This is usually the case for large broadcast transmitters, although special care must be taken when different transmitters are included in the same study (Schmiedel et al. 2009). On the contrary, distance from a base station is a poor indicator of exposure to RF-EMF indoors, due to the complex propagation characteristics of emissions from base station antennas, including shielding effects and multiple reflections from house walls and other buildings (Frei et al. 2010). Self-reported distance to transmitters is strongly affected by risk perception (Martens et al. 2017), and cannot be considered a reliable exposure indicator.

- 1. *Validity issues in the assessment of occupational RF exposure*

Exposure to RF-EMF can occur in the workplace, often at much greater levels than those experienced by the general public in the everyday environment. Occupational activities known to entail RF exposure include: operation and maintenance of high power telecommunication antennas; work in close proximity to navigation radar; professional use of hand-held transmitters (e.g., police officers, prison guards, truck-drivers); jobs/tasks related to industrial heating applications (e.g., induction heating of metals, dielectric heating/thawing/drying in various industries, plastic welding/sealing), or to medical applications of RF-EMF (physiotherapists using diathermy equipment) (AGNIR 2012; Hareuveny et al. 2015). A worker’s exposure level depends on characteristics of the RF source (including output power, modulation and field pattern), as well as on work-specific characteristics (including duration, worker’s location, and adherence to proper safety practice).

In general, exposure is better characterized in most occupational cohort studies than in geographical or ecological cohort studies or population- or hospital-based case-control studies (NTP-ORoC 2015). This may not be the case, however, for cohort studies of workers exposed to RF-EMF (AGNIR 2012; IARC 2013).

Recall bias is less likely to be a concern in case-control studies in which occupational exposure is assigned based on indirect measures from independent sources, than in studies using self-assessment of exposures (e.g., questionnaires with exposure checklists); for self-reported exposure, the exposure misclassification is often differential and biases towards an overestimate of the effect (NTP-ORoC 2015). In studies with exposure assessment based on job- or source-exposure matrices (JEM, SEM), different types of error can affect the two components of the matrix [(a) measurement-based data on exposure level by job/task; (b) individual occupational history data]. The potential for recall bias depend on the study design. JEM/SEM-based studies with occupational histories from independent records (e.g., census-based occupational cohort studies, industry-based cohort studies, and case-control studies nested in these cohorts), are susceptible to random exposure misclassification due to the inaccuracy/uncertainty of the exposure level estimates. When a JEM/SEM is used in a case-control study with self-reported occupational histories, both random errors and recall bias are of concern.

Few validation studies of indicators of occupational exposure to RF-EMF have been performed to date. In a cohort of British police officers, self-reported data on TETRA use were compared with objective radio usage records; for weekly use, participants under-reported the number of calls and over-reported the duration of calls by a factor of around 4 and 1.6 respectively, and bias was higher for daily usage (Vergnaud et al. 2016). Few details are provided on a validation of an industry-based JEM in the cohort study of Motorola employees, mentioned in the paper reporting on cancer mortality (Morgan et al. 2000).

| *Q8. Can we be confident in the exposure characterization?* |
| --- |
| Cohort, Case-Control (SR-A: RF exposure from wireless phones) |
| Definitely Low Risk of Bias (++) |
| - The exposure was assessed *prospectively* (i.e., before and independent of outcome), and *consistently* across study groups, that is over the same time frame and using the same method, consisting of: - for mobile phones: information about start-year of use and yearly calling time *for all mobile phones used* by a subject, collected *from independent sources* such as mobile phone operator records, supplemented by historical information about mobile phone frequency band, output power, phone model, base station density (urban/rural location), use of hands-free devices, preferred side of the head for mobile phone use, and including changes over time in any of the parameters, - for cordless phones: see Note 1, - **AND** the exposure was assessed in a time-window relevant for outcome development (Notes 2-3). |
| Probably Low Risk of Bias (+) |
| - The exposure was assessed *prospectively* (i.e., before and independent of outcome), and *consistently* across study groups, that is over the same time frame and using the same method, consisting of: - for mobile phones: information about the subjects’ start-year of mobile phone use collected from *independent* sources such as mobile phone operator records, but there was no information about amount of mobile phone use and other details mentioned above, **with** evidence that lack of information about amount of phone use and other details did not appreciably affect risk estimates (e.g., low prevalence of mobile phone use in the population and mobile phone use occurred primarily at a time period when mobile phone output power was generally high [prior to 1995 (Cardis et al. 2011)], - for cordless phones: see Note 1, - **OR** *prospectively* collected *self-reported* information about start year of wireless (mobile or cordless) phone use and calling time (e.g., through questionnaire/interview prior to disease occurrence), **with** evidence/indication that calling time information is representative for the exposure time window of interest, - **OR** *retrospectively* collected *self-reported* information about start year of wireless phone use and calling time (e.g., through questionnaire/interview after disease occurrence), capturing changes in the amount of phone use over time, **validated** and convincingly showing that reporting was not affected by the outcome (no recall bias), - **AND** the exposure was assessed in a relevant time-window for development of the outcome (Notes 2-3). |
| Probably High Risk of Bias (-) or (NR) |
| - The exposure was assessed through *retrospectively* collected *self-reported* information about start year of wireless phone use and calling time (e.g., data collection through questionnaire/interview after disease occurrence), capturing changes in the amount of phone use over time, **with validation** showing that reporting was likely affected by the outcome (evidence of recall bias), - **OR** there is insufficient information provided about the exposure assessment, including validity and reliability (record “NR” as basis for answer). |
| Definitely High Risk of Bias (--) |
| - There is indirect evidence that the exposure was assessed using poorly validated methods (i.e., retrospective self-reported information about wireless phone use that does not capture any changes in amount of use over time), - **OR** there is evidence that exposure is indicated for years prior to widespread use of mobile phones for the area under study [e.g., prior to late 1980’s-early 1990’s (Note 5)] - **OR** there are concerns about the method used (Note 6). |
| **Instruction reminder and notes**  Focus on the implications of the exposure assessment method on the validity of each exposure contrast of interest for the review, and assess the potential for specific types of exposure misclassification (see the blue box below).  For studies investigating the effect of both mobile and cordless phone, rate the potential for exposure-information bias separately for each type of wireless phone.  **Note 1**: For cordless phones, there are no objective sources of information about start date of exposure and amount of use over time. Therefore, consider ratings (+) or (-) for self-reported data collected before outcome ascertainment, and consider ratings (-) or (--) for self-reported data collected after outcome ascertainment.  **Note 2** [*Applies to analyses by time since start exposure*]: As there is no known mechanism for a carcinogenic effect of RF-EMF, various time windows (intervals between exposure start and outcome occurrence) need to be assessed, i.e., short term, intermediate term, and longer term exposure (usually defined as <5 years, 5-9 years, and 10+ years, respectively). The relevant time window differs between tumour subtypes. For slow growing tumours, such as meningioma and especially acoustic neuroma, short term exposure (<5 years, or perhaps even <10 years) is not expected to have any effect on tumour risk, and there is potential for reverse causation. For glioma, phone use that began within one year of date of diagnosis should not be considered (potential for reverse causation), and short term exposure (<5 years) is unlikely to have an effect.  Leukaemias (AML, CML, and ALL) are characterized by short induction periods following exposure to established risk factors; exposure lagging might not be required, and possible risk increases could be detectable even at short term exposure (<5 years).  **Note 3** [*Applies to analyses by cumulative exposure*]: If a substantial amount of mobile phone use is required for an effect on disease risk to occur, detailed prospective information about amount of use and changes in phone use habits over time is necessary. This also applies to cordless phones.  **Note 5:** Bag phones were introduced in 1980, but handheld mobile phones were not available in Europe before 1987. GSM phones were introduced in 1992-1993, and since then prevalence of mobile phone use increased substantially.  **Note 6:** Concern is based on generalizable information from other relevant exposure validation studies. |
| **In case of (-) or (- -) rating, for each exposure contrast, specify whether concern is for**  (toggle more than one if appropriate):   1. **Ever vs never exposed**  - Random exposure misclassification □ - Differential exposure misclassification (recall bias) □  1. **Time since start use**  - Random exposure misclassification □ - Outcome-dependent exposure misclassification (reverse causation) □ - Differential exposure misclassification (recall bias) □  1. **Total number of calls**  - Random exposure misclassification □ - Misclassification dependent on true exposure (systematic error) □ - Misclassification dependent on outcome (reverse causation) □ - Differential misclassification (recall bias) □  1. **Cumulative call time**  - Random exposure misclasification □ - Misclassification dependent on true exposure (systematic error) □ - Misclassification dependent on outcome (reverse causation) □ - Differential misclassification (recall bias) □ |

| *Q8. Can we be confident in the exposure characterization?* |
| --- |
| Cohort, Case-Control (SR-B: RF exposure from transmitters) |
| Definitely Low Risk of Bias (++) |
| - The exposure was assessed *prospectively* (i.e., before and independent of the outcome), and *consistently* across groups, that is over the same time-frame and using the same method, consisting of: - *measured or modelled electric or magnetic field levels or power density* of RF signals emitted from specific types of environmental (fixed-site) sources, relating to all/dominant microenvironments in the subjects’ daily life (e.g., home, school, workplace), taking into account technical and operating characteristics of the relevant transmitters [frequency band, power, irradiation pattern, start and stop dates of operation, (plus geographical density and network features in the study area for base stations)], and including changes over time in any of the parameters. - **AND** exposure was assessed in a time-window relevant for outcome development (Notes 1-2). |
| Probably Low Risk of Bias (+) |
| - The exposure was assessed *prospectively* and *consistently* across groups, using - *measured or modelled* electric or magnetic field levels or power density of RF signals emitted from specific types of environmental (fixed-site) sources, in all/dominant microenvironments in subjects’ daily life (e.g., home, school, workplace), but there was no historical information about technical and operating characteristics of the relevant transmitters or other details, **with** evidence that lack of historical information about technical and operating characteristics of the relevant transmitters or other details would not appreciably affect risk estimates (e.g., little change in analogue radio and TV broadcasting over time), - **OR** [for broadcast transmitters, NOT for base stations] *geocoded distances* based on addresses collected from independent sources (e.g., population registries with residential histories), **with** evidence/indication that they are representative for the exposure time window of interest, - **OR** [for broadcast transmitters, NOT for base stations] *geocoded distances* from self-reported addresses, **validated** and convincingly showing that exposure misclassification is independent of the true exposure and/or of the outcome (i.e., no systematic or differential bias), and that the exposure variables are representative for the exposure time window of interest. - **AND** exposure was assessed in a relevant time-window for development of the outcome (Notes 1-2). |
| Probably High Risk of Bias (-) or (NR) |
| - The exposure was assessed through models with limited predictive performance or omitting relevant sources (i.e., evidence of information bias), - **OR** there is insufficient information provided about the exposure assessment, including validity and reliability (record “NR” as basis for answer). |
| Definitely High Risk of Bias (--) |
| - The exposure was assessed using poorly validated methods (i.e., retrospective self-reported information about distance to any types of transmitters, or geocoded distances to base stations), - **OR** there is evidence that exposure occurred outside the appropriate time window to potentially affect the outcome (Notes 2- 3), - **OR** there is evidence for concern about the method used (Note 3). |
| **Instruction reminder and notes**  Focus on the implications of the exposure assessment method on the validity of each exposure contrast of interest for the review, and assess the potential for specific types of exposure misclassification (see the blue box below).  For studies investigating the effect of more than one type of transmitters (e.g., broadcast antennas and base stations), rate the potential for exposure-information bias separately for each type of exposure source.  **Note 1** [*Applies to analyses by time since start exposure*]: As there is no known mechanism for a carcinogenic effect of RF-EMF, various time windows (intervals between exposure start and outcome occurrence) need to be assessed (i.e., short term, intermediate term, and longer term exposure, usually defined as <5 years, 5-9 years, and 10+ years), respectively). The relevant time window differs between tumour subtypes. For slow growing tumours, such as acoustic neuroma and meningioma, short term exposure (<5 years, or perhaps even <10 years) is not expected to have any effect on tumour risk (and there is potential for reverse causation). For glioma, a malignant solid tumour, phone use that began within one year of date of diagnosis should not be considered (potential for reverse causation), and short term exposure (<5 years) is unlikely to have an effect.  Leukaemias (AML, CML, and ALL) are characterized by short induction periods following exposure to known carcinogens such as ionising radiation at moderate/high doses (medical), and cytotoxic chemotherapy (Linet et al. 2018); exposure lagging might not be required, and possible risk increases could be detectable even at short term exposure (<5 years).  **Note 2** [*Applies to analyses by cumulative exposure*]: If a substantial amount of exposure is required for an effect on disease risk to occur, detailed prospective information about changes in exposure level over time is necessary.  **Note 3:** Concern is based on generalizable information from other relevant exposure validation studies. |
| **In case of (-) or (- -) rating, for each exposure contrast, specify whether concern is for** (toggle more than one if appropriate):   1. **Ever vs never exposed**  - Random exposure misclassification □ - Differential exposure misclassification (recall bias) □  1. **Time since start exposure or exposure duration**  - Random exposure misclassification □ - Outcome-dependent exposure misclassification (reverse causation) □ - Differential exposure misclassification (recall bias) □  1. **Average or cumulative exposure level**  - Random exposure misclassification □ - Misclassification dependent on true exposure (systematic error) □ - Misclassification dependent on outcome (reverse causation) □ - Differential misclassification (recall bias) □ |

| *Q8. Can we be confident in the exposure characterization?* |
| --- |
| Cohort, Case-Control (SR-C: Occupational RF exposure) |
| Definitely Low Risk of Bias (++) |
| - The exposure was assessed *prospectively*, *independent of the outcome*, and *consistently* across study groups, that is, over the same time-frame and using the same method, consisting of:   - For RF-exposure from professional use of hand-held transceivers: information about the subjects’ start-year of use and yearly amount (time) of use, collected from independent sources such as company records, supplemented by information about the device frequency band, operational characteristics, usage modalities, preferred side of the head for use, and including changes over time in any of the parameters.   - For other RF-emitting equipment in the workplace: measured electric or magnetic field levels or power density of RF signals at the worker location during relevant tasks, supplemented by historical information (at the individual level) on proximity to the equipment during relevant tasks, and task frequency and duration, including changes over time in any of the parameters. - **AND** exposure was assessed in a time-window relevant for outcome development (Notes 1-2). |
| Probably Low Risk of Bias (+) |
| - The exposure was assessed *prospectively* and *consistently* across groups, using the methods and metrics described above, but there was no information about (a) the amount of hand-held transceiver use, or (b) job/task characteristics or other details, **with** evidence that lack of information about (a) the amount of hand-held transceiver use, or (b) job/task characteristics, would not appreciably affect risk estimates (e.g., low prevalence of hand-held transceiver usage or no change in exposure level across different jobs/tasks over time), - **OR** exposure was assessed using *prospectively collected self-reported information* (e.g., through questionnaire/interview prior to disease occurrence) about (a) hand-held transceiver use, or (b) jobs/tasks involving exposure to RF-EMF including task frequency and duration, **with** evidence that information about hand-held transceiver use or jobs/tasks is representative for the exposure time window of interest, - **OR** estimates of cumulative exposure were obtained combining RF exposure levels from source- or job-exposure matrices (SEM or JEM) with *occupational histories from independent sources* (e.g., census data), or with *prospectively collected self-reported occupational histories* and jobs/tasks with or nearby RF-sources (e.g., through questionnaire/interview before disease occurrence), - **OR** exposure was assessed using *retrospectively collected self-reported information* (e.g., through questionnaire/interview after disease occurrence) about (a) hand-held transceiver use, or (b) jobs/tasks involving exposure to RF-EMF including task frequency and duration, **validated** and convincingly showing that reporting was not affected by the outcome (no recall bias), - **OR** estimates of cumulative exposure were obtained combining RF exposure levels from SEM/JEM with *retrospectively collected self-reported* occupational histories and jobs/tasks with or nearby RF-sources (e.g., through questionnaire/interview after disease occurrence), **validated** and convincingly showing that that reporting was not affected by the outcome (no recall bias), - **AND** exposure was assessed in a relevant time-window for development of the outcome (Notes 1-2). |
| Probably High Risk of Bias (-) or (NR) |
| - The exposure was assessed through retrospectively collected self-reported information about (a) hand-held transceiver use, or (b) jobs/tasks involving exposure to RF-EMF, including task frequency and duration, **with validation** showing that reporting was likely affected by the outcome (i.e., evidence of recall bias), - **OR** estimates of cumulative exposure were obtained combining RF exposure levels from SEM/JEM with retrospectively collected self-reported occupational histories and jobs/tasks with or nearby RF-sources (e.g., through questionnaire/interview after disease occurrence), **with validation** showing that the exposure variable was likely affected by the outcome (i.e., evidence of recall bias), - **OR** there is insufficient information provided about the exposure assessment, including validity and reliability (record “NR” as basis for answer). |
| Definitely High Risk of Bias (--) |
| - The exposure was assessed using poorly validated methods (i.e., retrospective self-reported information about (a) hand-held transceiver use, or (b) jobs/tasks involving exposure to RF-EMF, that does not capture any changes in exposure level over time), - **OR** there is evidence that exposure occurred outside the appropriate time window to potentially affect the outcome (Notes 1-2), **OR** there is evidence for concern about the method used (Note 3). |
| **Rating instruction reminder and notes**  Focus on the implications of the exposure assessment method on the validity of each exposure contrast of interest for the review, and assess the potential for specific types of exposure misclassification (see the blue box below).  For studies investigating the effect of more than one RF-emitting equipment or device, rate the potential for exposure-information bias separately for each type of exposure source.  **Note 1** [*Applies to analyses by time since start exposure*]: As there is no known mechanism for a carcinogenic effect of RF-EMF, various time windows (intervals between exposure start and outcome occurrence) need to be assessed (i.e., short term, intermediate term, and longer term exposure, usually defined as <5 years, 5-9 years, and 10+ years), respectively). The relevant time window differs between tumour subtypes. For slow growing tumours, such as acoustic neuroma and meningioma, short term exposure (<5 years, or perhaps even <10 years) is not expected to have any effect on tumour risk (and there is potential for reverse causation). For glioma, a malignant solid tumour, phone use that began within one year of date of diagnosis should not be considered (potential for reverse causation), and short term exposure (<5 years) is unlikely to have an effect.  Leukaemias (AML, CML, and ALL) are characterized by short induction periods following exposure to known carcinogens such as ionising radiation at moderate/high doses (medical), and cytotoxic chemotherapy (Linet et al. 2018); exposure lagging might not be required, and possible risk increases could be detectable even at short term exposure (<5 years).  **Note 2** [*Applies to analyses by cumulative exposure*]: If a substantial amount of mobile phone use is required for an effect on disease risk to occur, and it can reasonably be assumed that only a very small proportion of mobile phone users have used a mobile phone to this extent, detailed prospective information about amount of exposure and changes in exposure level over time is necessary.  **Note 3:** Concern is based on generalizable information from other relevant exposure validation studies. |
| **In case of (-) or (- -) rating, for each exposure contrast, specify whether concern is for** (toggle more than one if appropriate):   1. **Ever vs never exposed**  - Random exposure misclassification □ - Differential exposure misclassification (recall bias) □  1. **Time since start exposure or exposure duration**  - Random exposure misclassification □ - Outcome-dependent exposure misclassification (reverse causation) □ - Differential exposure misclassification (recall bias) □  1. **Average or cumulative exposure level**  - Random exposure misclassification □ - Misclassification dependent on true exposure (systematic error) □ - Misclassification dependent on outcome (reverse causation) □ - Differential misclassification (recall bias) □ |

1. **Confidence in outcome assessment**

The OHAT’s instructions highlight three important factors for assessing bias in the outcome assessment: the objectivity of the outcome assessment; consistency in measurement of outcomes; and blinding of the outcome assessors (for knowledge of the exposure). Objectivity of the outcome assessment and the need for blinding are two sides of the same issue. Blinding requires that outcome assessors do not know the study group or exposure level of the human subject when the outcome was assessed. The objectivity of procedures used for measuring and reporting an outcome will impact the degree to which outcome assessors could bias the reported results. Acceptable methods for measuring the outcomes of interest will be highly dependent on the outcome and therefore a specific list of acceptable, inaccurate, or potentially biased methods should be developed for each evaluation and will require subject-matter expertise [(NTP-OHAT 2015), pp. 25-26].

Incidence data from population-based cancer registries, hospital clinical records, and pathology data are generally more detailed and accurate than death certificate data. Cancer incidence data are considerably more informative than mortality data for cancers with long survival and good prognosis (such as benign CNS tumours, thyroid cancer, or cancer of the testis). The classification of subtypes of some tumours (e.g., neoplasms of haematopoietic or lymphoid tissues) has changed over time, and may present challenges if histological data are unavailable to confirm subtypes. Therefore, the potential for misclassification may vary over time, and is greater when case ascertainment is based on death certificates. For cancers with low survival (e.g., lung and pancreatic cancers) incidence and mortality data may be of similar utility (NTP-ORoC 2015). Mortality data are not contemplated in the Q9 answer options for case-control studies, because we restricted eligibility for inclusion to mortality-based cohort studies. Two issues relevant to our research topic are discussed below.

- 1. *Detection bias*

Detection bias is due to systematic differences between the study groups in ascertainment, assessment, diagnosis, or verification of outcomes (Porta 2016). In terms of structure, it is a form of dependent differential misclassification of the outcome. In studies of acoustic neuroma, mobile phone use can raise awareness about the unilateral hearing loss that is an early symptom of the disease, facilitating or anticipating the diseases diagnosis. Physicians or otorhinolaryngologists, suspecting that mobile phone use causes acoustic neuroma, may monitor patients using mobile phones (or heavy users) more closely than non-users (or low-users). Consequently, a differential measurement error of the outcome will occur, wherein the exposure affects the likelihood of (an early) diagnosis. Detection bias, whose upward direction is easily predictable, can affect risk estimates from both cohort and case-control studies. Together with the increasing accessibility of neuroimaging resulting in higher rates of incidentally diagnosed acoustic neuroma and other benign CNS tumours (Cote and Laws 2017), detection bias may have contributed to the increasing incidence rate of acoustic neuroma accompanied by a parallel decrease in tumour size at diagnosis, observed in a 40-year time trend study in Denmark (Reznitsky et al. 2019).

- 1. *Multiple validity issues in studies of central nervous system tumours*

Both malignant and benign CNS tumours can inhibit essential brain functions, and substantially change the lifestyle habits of patients. Pre-treatment impaired cognition is common in low-grade or high-grade gliomas, and meningioma (Irestorm et al. 2018; Meskal et al. 2016; van Kessel et al. 2017; van Loon et al. 2015). Most malignant CNS tumours are rapidly fatal, with an average 5-year survival rate of about 33% (Amirian et al. 2018). Due to these features, epidemiological studies of risk factors for CNS tumours, especially case-control studies (Johansen et al. 2017), are prone to multiple biases (selection, attrition/exclusion, exposure- and outcome-information biases) and rapid case ascertainment is essential for minimizing the potential for these distortions.

| *Q9. Can we be confident in the outcome assessment?* |
| --- |
| Cohort (SR-A\|SR-B\|SR-C) |
| Definitely Low Risk of Bias (++) |
| - Direct evidence that the outcome was assessed using objective diagnostic measures, and all eligible newly diagnosed (incident) cancer/tumour cases were identified through sources with complete coverage of all eligible subjects during the entire follow-up period (e.g., record-linkage to population based cancer registers of high quality, record-linkage to nationwide registers of hospital admissions with available information on date of diagnosis, and/or hospital based case recruitment with evidence that the hospital(s) had a well-defined catchment area, with exhaustive/high coverage of the source population during the entire follow-up period), - **AND** follow-up time was adequately recorded to allow calculation of person-years at risk, or subjects had been followed for the same length of time in all study groups, - **AND** there is direct evidence that the outcome assessors were unaware of the exposure at the time of diagnosis, and it is unlikely that they could have been influenced by the exposure when reporting outcomes (low risk of detection bias). |
| Probably Low Risk of Bias (+) |
| - Indirect evidence that the outcome was assessed using objective diagnostic measures and acceptable methods of case-ascertainment were used (e.g., hospital based case recruitment with evidence that the hospital had a well-defined catchment area with fair coverage of the source population during the entire follow-up period, and limited migration), **OR** it is deemed that the outcome assessment methods used would not appreciably bias results (e.g., death certificate data from mortality databases – option only applicable to malignancies with low survival/poor prognosis, such as lung cancer or pancreatic cancer, and not affected by coding changes over time), - **AND** follow-up time was adequately recorded to allow calculation of person-years at risk, or subjects had been followed for the same length of time in all study groups, - **AND** there is indirect evidence that the outcome assessors were unaware of the exposure at the time of diagnosis, and it is unlikely that they could have been influenced by the exposure when reporting outcomes (low risk of detection bias), **OR** it is deemed that lack of adequate blinding of outcome assessors would not appreciably bias results, which is more likely to apply to objective (opposed to self-reported) outcome measures (low risk of detection bias). |
| Probably High Risk of Bias (-) or (NR) |
| - Indirect evidence that, although the outcome was assessed using objective diagnostic measures, the case ascertainment method resulted in incomplete case recruitment (e.g., hospital based case identification with unknown coverage of the source population), - **OR** outcome assessment was based on cancer-specific mortality data from nationwide death registries for tumours with low fatality rate/long-survival and/or affected by coding changes over time, - **OR** the length of follow up differed by study group, - **OR** there is indirect evidence that it was possible for outcome assessors to be influenced by the exposure when assessing the outcome, including subjects’ own awareness of symptoms (high risk detection bias). |
| Definitely High Risk of Bias (--) |
| - Direct evidence that the outcome assessment method is an insensitive instrument. - **OR** the length of follow up differed by study group, - **OR** completeness of case recruitment varied over time or with vital status of the patients, - **OR** there is direct evidence that it was possible for outcome assessors to be influenced by the exposure when assessing the outcome, including subjects’ own awareness of symptoms (high risk of detection bias). |

| *Q9. Can we be confident in the outcome assessment?* |
| --- |
| Case-Control (SR-A\|SR-B\|SR-C) |
| Definitely Low Risk of Bias (++) |
| - Direct evidence that the outcome was assessed using objective diagnostic measures, and all newly diagnosed (incident) cancer/tumour cases were identified using sources with complete coverage of all eligible subjects during the entire study period (e.g., population based cancer registers of high quality, and/or hospital based case recruitment with evidence that the hospital(s) had a well-defined catchment area, with exhaustive/high coverage of the source population over the study timeframe (see Note), - **AND** subjects had been followed for the same length of time in all study groups, **OR** the time-window for case ascertainment (respect to the natural history of the disease) did not vary across study groups, - **AND** there is direct evidence that the outcome assessors were unaware of the exposure at the time of diagnosis, and it is unlikely that they could have been influenced by the exposure when reporting outcomes (low risk of detection bias). |
| Probably Low Risk of Bias (+) |
| - Indirect evidence that the outcome was assessed using objective diagnostic measures, and acceptable methods of case-ascertainment were used (e.g., hospital based case recruitment with evidence that the hospital had a well-defined catchment area, with fair coverage of the source population during the entire study period and limited migration (see Note), **OR** it is deemed that the outcome assessment methods used would not appreciably bias results, - **AND** subjects had been followed for the same length of time in all study groups, **OR** the time-window for case ascertainment (respect to the natural history of the disease) did not vary across study groups, - **AND** there is indirect evidence that the outcome assessors were unaware of the exposure at the time of diagnosis, and it is unlikely that they could have been influenced by the exposure when reporting outcomes (low risk of detection bias). |
| Probably High Risk of Bias (-) or (NR) |
| - Indirect evidence that, although the outcome was assessed using objective diagnostic measures, the case ascertainment method resulted in incomplete case recruitment (e.g., hospital based case identification with unknown coverage of the source population), - **OR** the length of the observation period differed by study group, - **OR** there is indirect evidence that it was possible for outcome assessors to be influenced by the exposure when assessing the outcome, including subjects’ own awareness of symptoms (high risk of detection bias). |
| Definitely High Risk of Bias (--) |
| - Direct evidence that the outcome assessment method is an insensitive instrument, - **OR** the length of observation period differed by study group, - **OR** completeness of case recruitment varied over time or with vital status of the patients, - **OR** there is direct evidence that it was possible for outcome assessors to be influenced by the exposure when assessing the outcome, including subjects’ own awareness of symptoms (high risk of detection bias). |
| **Note**: Rapid ascertainment in case-control studies of CNS tumours is essential for minimizing the potential for outcome-information bias and other biases (selection, data missingness, and exposure-information). |

1. **Selective reporting**

Selective reporting is present if pre-specified outcomes are not reported or incompletely reported. It is likely widespread and difficult to assess with confidence for most studies unless the study protocol is available. Selective reporting bias can be assessed by comparing the “methods” and “results” section of the paper, and by considering outcomes measured in the context of knowledge in the field. Abstracts of presentations relating to the study may contain information about outcomes not subsequently mentioned in publications. Selective reporting bias should be suspected if the study does not report outcomes in the results section that would have been expected based on the methods, or if a composite score is presented without the individual component outcomes [Excerpts from (NTP-OHAT 2015), p.30].

| *Q10. Were all measured outcomes reported?* |
| --- |
| Cohort, Case-Control (SR-A\|SR-B\|SR-C) |
| Definitely Low Risk of Bias (++) |
| - Direct evidence that all of the study’s measured outcomes (primary and secondary) outlined in the protocol, methods, abstract, and/or introduction, or mentioned in the discussion (that are relevant for the evaluation) have been reported. This would include: outcomes reported with sufficient detail to be included in meta-analysis or fully tabulated during data extraction and analyses had been planned in advance; reporting of exposure and outcome variable corresponding to the anticipated analysis plan (e.g. predefined cut-offs in case of categorization). |
| Probably Low Risk of Bias (+) |
| - Indirect evidence that all of the study’s measured outcomes (primary and secondary) outlined in the protocol, methods, abstract, and/or introduction, or mentioned in the discussion (that are relevant for the evaluation) have been reported, - **OR** analyses that had not been planned in advance (i.e., unplanned/post-hoc subgroup analyses) are clearly indicated as such, **AND** it is deemed that unplanned analyses were appropriate and selective reporting would not appreciably bias results (e.g., appropriate analyses of an unexpected effect). This would include: outcomes reported with insufficient detail, such as only reporting that results were statistically significant (or not); exposure-response modelling adhering to the anticipated analysis plan (e.g. predefined cut-offs in case of categorization). |
| Probably High Risk of Bias (-) or (NR) |
| - Indirect evidence that all of the study’s measured outcomes (primary and secondary) outlined in the protocol, methods, abstract, and/or introduction, or mentioned in the discussion (that are relevant for the evaluation) have not been reported, - **OR** there is indirect evidence that unplanned analyses, or unplanned exposure-response modelling (e.g., predefined cut-offs in case of categorization), were included that may appreciably bias results, - **OR** there is insufficient information provided about selective reporting (record “NR” as basis for answer). |
| Definitely High Risk of Bias (--) |
| - Direct evidence that all of the study’s measured outcomes (primary and secondary) outlined in the protocol, methods, abstract, and/or introduction (that are relevant for the evaluation) have not been reported, - **OR** Direct evidence of selective reporting. This would include: outcomes reported using measurements, analysis methods or subsets of the data that were not pre-specified, or reporting outcomes not pre-specified, or that unplanned analyses were included that would appreciably bias results. |

1. **Appropriateness of statistical methods**

| *Q11. Were appropriate statistical methods used?* | | |
| --- | --- | --- |
| Cohort, Case-Control (SR-A\|SR-B\|SR-C) | | |
| **Yes** | **No** | **If No, provide details** |
|  |  |  |

# APPENDIX

1. **Analytical framework used to identify critical potential confounders**

The identification of the critical potential confounders for the exposure-outcome associations of primary interest for our systematic review relied on the elements below.

We examined the available evidence on predictors of the outcomes (D_i_) that could also be antecedents of the exposures (E_i_); prevalence of exposure to these factors (C_i_) in the target population; and the plausibility and expected magnitude of the E_i_-C_i_ association. We also considered the implications of incomplete exposure assessment because, when potentially hazardous agents have multiple sources, the effects of exposure from one source can be confounded by exposure to other sources of the same agent (Loomis and Savitz 1994).

If E_i_ and C_i_ are unlikely to be associated, or the prevalence of C_i_ is predictably low in the study population, one can confidently exclude that confounding from C_i_ is of relevant concern in the assessment of the E_i_-D_i_ association (Savitz and Wellenius 2016; VanderWeele 2019).

- 1. *Predictors and risk factors for the neoplasms of primary interest*

The incidence rates of central nervous system (CNS) neoplasms, and their relative distribution by site and histology, vary notably by sex and age (Lapointe et al. 2018; Ostrom et al. 2020).

New cases of malignant tumours of the brain (*syn*. brain cancer) are recorded by all cancer registries. Brain cancer incidence rates vary across world regions (from 1-2 per 100,000 in eastern Asia and Africa to 6-7 per 100,000 in Europe), with a male to female (m/f) ratio always above one (Barnholtz-Sloan et al. 2018). Gliomas account for the majority (75-80%) of all malignant brain tumours in adults (Lapointe et al. 2018; Ostrom et al. 2020). Paediatric brain tumours include benign subtypes (e.g., pilocytic astrocytoma, embryonal tumours, and ependymal tumours) uncommon in adults (Udaka and Packer 2018), but the established risk factors for brain tumours do not differ by age at diagnosis (Ostrom et al. 2019).

There are wide variations across cancer registries in coverage of benign CNS tumours, as well as in accessibility to neuroimaging by geography, calendar time and age (Karipidis et al. 2018; 2019; Larjavaara et al. 2011; McCarthy et al. 2013; Tettamanti et al. 2019). This may result in heterogeneous case series (including variable proportions of cases at different stages of progression at diagnosis) in multicentre case-control studies.

Since 2003, the Central Brain Tumor Registry of the United States (CBRTUS) records all incident cases of benign and malignant CNS tumours. According to the CBRTUS’s report relating to the period 2013-2017 (Ostrom et al. 2020), non-malignant tumours represent 70% of all primary CNS tumours, and the most common subtypes are meningiomas (38%; incidence rate 8.7 per 100,000) and pituitary tumours (17%; 4.2 per 100,000), both of which occur more frequently in women than in men (m/f ratio of 0.4 and 0.8, respectively).

Acoustic neuroma (*syn*. vestibular schwannoma) account for about 8% of all CNS tumours, with an incidence rate of 1.2 per 100,000, and no sex differences in occurrence (Ostrom et al. 2020).

Salivary gland neoplasms most often arise in the parotid gland (70-80%). Benign histotypes (pleomorphic adenoma, and Warthin tumours) account for the majority of cases (Bradley 2016; Zhan et al. 2016), but are not recorded by cancer registries. Estimates of the overall incidence of salivary gland tumours vary between 5 and 8 per 100,000 (Andreasen et al. 2016; Bradley and McGurk 2013).

Ionizing radiation (IR) at moderate-to-high (medical) doses increases the risk of all types of CNS tumours (Amirian et al. 2018; Ostrom et al. 2019), as well as of salivary gland tumours (Hashibe et al. 2018). The median latency of radiation-induced CNS tumours varies across histology groups; it is 9 to 18 years (at least 5 years) for gliomas, and 17 to 23 years (min 10 years) for meningiomas (Lee and Wernicke 2016; McNeill 2016; Yamanaka et al. 2017; 2018). There is scanty information about latency of radiation-induced acoustic neuroma; it ranged from 20 to 55 years in children irradiated (1939-1962) to reduce the size of their tonsils and adenoids (Schneider et al. 2008).

Persons with a history of allergy or other atopic diseases are at decreased risk of CNS neoplasms, particularly of glioma (Ostrom et al. 2019). Smoking is not associated with the incidence of glioma (Shao et al. 2016), meningioma (Fan et al. 2013), or pituitary tumours (Benson et al. 2010). The reasons behind the reduced risks of acoustic neuroma in smokers, reported by several studies, are unclear (Ostrom et al. 2019). Socioeconomic status (SES) is positively correlated with incidence of CNS tumours; for malignant CNS neoplasms, the increased risk among individuals of high SES compared to those of low SES seems not entirely attributable to improved access to medical care (Ostrom et al. 2019).

Leukaemia incidence rates show large geographical variations (Bray et al. 2018). Different subtypes of leukaemia dominate at different ages. The most common subtype in children is acute B-lymphoblastic leukaemia (B-ALL), while acute myeloid leukaemia (AML) and chronic lymphocytic leukaemia (CLL) are far more common at older age; almost every leukaemia subtype occurs more frequently in males than females (Roman and Smith 2020).

IR at moderate-to-high (medical) doses is an established risk factor for all leukaemias other than CLL (Berrington de Gonzalez et al. 2018; Linet et al. 2018). A recent systematic review provides evidence in favour of excess leukaemia risk (in adults and children) from exposure to low-dose (<100 mGy) IR (Hauptmann et al. 2020).

Benzene is an established cause of AML in adults, and consistent positive associations have also been observed for CLL and other leukaemia subtypes in adults, as well as for AML in children (IARC 2018). Other risk factors for adult AML include tobacco smoking, cytotoxic chemotherapy, occupational exposures to formaldehyde, styrene, 1,3-butadiene, and work in rubber manufacturing (Linet et al. 2018; World Cancer Report 2020).

Epidemiological and modelling studies endorse a dual role for common infections in the etiology of childhood ALL; microbial exposures earlier in life are protective but, in their absence, later infections trigger the critical secondary mutations (Greaves 2018). Other factors associated to childhood leukaemia with convincing level of evidence include Down syndrome, birth weight, parental age, and geography (Schüz and Erdmann 2016). The association of childhood leukaemia with place of residence (across or within countries) can be due to several contributing factors, including diagnostic and referral patterns, completeness of registration and documentation, and true geographic differences in incidence rates (Schüz and Erdmann 2016). Space-time clustering of childhood leukaemia have been observed in many studies, but the clusters are often limited to specific histologic or molecular subtypes, classes of age at diagnosis, or residence timing (at birth *vs* at diagnosis), and are not robust to control for multiple testing (Francis et al. 2020; Konstantinoudis et al. 2017; Kreis et al. 2017).

Despite considerable variations across leukemogens and leukaemia subtypes (Linet et al. 2018), it is widely accepted that leukaemia latency is short (Linet et al. 2020). Most studies investigating the effects of low-dose IR on non-CLL leukaemia risk have calculated cumulative exposure indices using a lag of 2 years (Gilbert et al. 2020).

- 1. *Shared antecedents of exposure and outcome*

At the time when first generation (1G) handheld mobile phones were made available (1984 in the United States; 1987 in the Nordic Countries), and up to early 1990s, the devices were very expensive, the cost for calls was very high, and only a small proportion of the population, mainly better off males aged 30-60 years (SCENIHR 2009), were using them. Following the introduction of 2G technology, mobile phone usage became less expensive, the number of mobile phone users increased dramatically, and the age, sex, and SES differentials dissolved. In Sweden, for example, the number of subscriptions/population size was <10% in 1993 and over 100% in 2005; similar time trend, although asynchronous across countries, have been documented worldwide (ITU 2020). With further decreases of costs for calls and the introduction of smartphones in late-2000s, the amount of mobile phone use (call minutes) also increased considerably. Therefore, the association between mobile phone ownership/use and SES varies over time, within and between countries, according to country-specific changes in use prevalence overall and by region, sex and age strata. Sex, age, and SES are common predictors of exposure to RF-EMF from mobile phone use and risk of CNS and salivary gland tumours. Due to geographic differences in registration of CNS tumours (especially benign subtypes), and in timing of mobile phone use spread, control/adjustment for country/region is required in multicentre case-control studies.

Environmental levels of RF-EMF from base stations increased during the 1990’s, while remained stable since the introduction of 3G networks in early 2000s (Jalilian et al. 2019; Rowley and Joyner 2012). Besides sex and age, control for time period is required in studies of cancer risk in relation to exposure from transmitters.

In the occupational setting, confounding from co-exposures to benzene, 1,3-butadiene, formaldehyde, work in rubber manufacturing, and tobacco smoking is of concern in the assessment of leukaemia risk, and exposure to IR is of concern in the assessment of both leukaemia and CNS tumour risks. Sex, age, and SES should also be controlled/adjusted for. Confounding from time period should also be taken into account even though, according to the authors of a large national survey spanning the decade 1995-2005, the variability in RF exposure over time was comparatively less than that observed across jobs, tasks and workplaces (Hareuveny et al. 2015).

- 1. *Factors unlikely to be strong confounders*

Diagnostic/therapeutic exposure to IR is unlikely to be strongly associated with mobile phone use; adjustment for these exposures was inconsequential in the Interphone case-control study (Blettner et al. 2007; Interphone Study Group et al. 2010). Control for SES in the analyses would suffice to address possible socio-economic differentials in access to CT or NMR scans. Even more unlikely is a correlation between medical exposure to IR, or residential exposure to radon, and exposure to RF-EMF from fixed-site transmitters. Furthermore, in case-control studies of cancer with self-reported information on medical exposure to IR, adjustment for imaging/radiotherapies undertaken for the diagnosis or treatment of the investigated tumour can introduce bias.

Exposures to most established risk factors for leukaemias are rare in the general population, and all (including benzene) are unlikely to be associated with RF exposure from transmitters.

We feel confident in excluding a correlation between other established risk factors for childhood leukaemia (infectious agents, Down syndrome, birth weight, and parental age) and RF-EMF from transmitters.

- 1. *Issues related to multiple exposure sources*

Exposure to environmental (fixed-site) sources of RF-EMF is unlikely to confound the associations between wireless phone use and risks of CNS or salivary gland tumours (of primary interest in SR-A). In fact, only when smartphones became common and 3G networks were fully deployed (around late 2000s in Europe), one can expect a substantial reduction in the dominant contribution of mobile phones to the estimated RF brain-dose, compared to far-field RF sources (Cabré-Riera et al. 2020; Lauer et al. 2013). However, the proportion of mobile phone users with exposure start dates after late-2000s was either null or very low in studies published up to 2017, as reviewed in (Röösli et al. 2019). For example, the exposure start dates among the exposed sub-groups of the Danish and UK cohort studies were, respectively, 1987-1995 (Frei et al. 2011) and prior or equal to 1999-2005 (Benson et al. 2013). As to the case-control studies, the upper limit of the diagnosis/reference dates was before 2007 in all studies, with the single exception of one Swedish study enrolling CNSn cases diagnosed in 2007-2009, where the proportion of all mobile phone user controls classified as “3G users” was about 11% (Carlberg et al. 2013; Hardell et al. 2013).

Exposure to near-field sources of RF-EMF is unlikely to be correlated with exposure from broadcast transmitters. Exposure from mobile phone use might be negatively correlated with exposure from base stations (Wall et al. 2019); however, the correlation would be limited to uplink and downlink RF signals from one’s own provider, lessening the importance of reciprocal confounding effects in studies of either near-field or far-field exposures.

Mobile phones are used for professional and/or private reasons. However, as no specific question on mobile usage reason/setting was included in the questionnaire/interview used by most studies on mobile phone use and cancer risk carried out so far, it is reasonable to assume that respondents reported about their overall use. Thus this potential source of confounding from incomplete exposure assessment may be a minor issue in SR-A.

Occupational exposure to RF-EMF is unlikely to confound the associations investigated in SR-A, based on available data (Berg et al. 2006; Coureau et al. 2014; Interphone Study Group et al. 2010).

Mobile phone use may vary across and within industrial sectors, occupations and type of employment, but ancillary evidence on this topic is sparse (Caban-Martinez et al. 2020). Control of confounding from mobile phone use was not deemed necessary in a recent case-control studies of occupational RF exposure and risk of brain tumours (Vila et al. 2018).

# REFERENCES

AGNIR. Health effects from radiofrequency electromagnetic fields. London: Health Protection Agency; 2012.

Amirian, E.S., Ostrom, Q.T., Liu, Y., Barnholtz-Sloan, J., Bondy, M.L. Nervous System. in: Thun M., Linet M.S., Cerhan J.R., Haiman C.A., Schottenfeld D., eds. Cancer Epidemiology and Prevention Fourth edition. Oxford: Oxford University Press; 2018.

Andreasen, S., Therkildsen, M.H., Bjorndal, K., Homoe, P. Pleomorphic adenoma of the parotid gland 1985-2010: A Danish nationwide study of incidence, recurrence rate, and malignant transformation. Head Neck 2016;38 Suppl 1:E1364-1369.

Andrews, N., Miller, E., Taylor, B., Lingam, R., Simmons, A., Stowe, J., Waight, P. Recall bias, MMR, and autism. Arch Dis Child 2002;87:493-494.

Auvinen, A., Feychting, M., Ahlbom, A., Hillert, L., Elliott, P., Schuz, J., Kromhout, H., Toledano, M.B., Johansen, C., Poulsen, A.H., Vermeulen, R., Heinavaara, S., Kojo, K., Tettamanti, G., Group, C.S. Headache, tinnitus and hearing loss in the international Cohort Study of Mobile Phone Use and Health (COSMOS) in Sweden and Finland. Int J Epidemiol 2019;

Aydin, D., Feychting, M., Schüz, J., Andersen, T.V., Poulsen, A.H., Prochazka, M., Klaeboe, L., Kuehni, C.E., Tynes, T., Röösli, M. Predictors and overestimation of recalled mobile phone use among children and adolescents. Prog Biophys Mol Biol 2011;107:356-361.

Barnholtz-Sloan, J.S., Ostrom, Q.T., Cote, D. Epidemiology of brain tumors. Neurol Clin 2018;36:395-419.

Benson, V.S., Green, J., Pirie, K., Beral, V. Cigarette smoking and risk of acoustic neuromas and pituitary tumours in the Million Women Study. Br J Cancer 2010;102:1654-1656.

Benson, V.S., Pirie, K., Schüz, J., Reeves, G.K., Beral, V., Green, J., Million Women Study, C. Mobile phone use and risk of brain neoplasms and other cancers: prospective study. Int J Epidemiol 2013;42:792-802.

Berg, G., Spallek, J., Schüz, J., Schlehofer, B., Bohler, E., Schlaefer, K., Hettinger, I., Kunna-Grass, K., Wahrendorf, J., Blettner, M., Interphone Study Group, G. Occupational exposure to radio frequency/microwave radiation and the risk of brain tumors: Interphone Study Group, Germany. Am J Epidemiol 2006;164:538-548.

Berrington de Gonzalez, A., Bouville, A., Rajaraman, P., Schubauer-Benigan, M. Ionizing radiation. in: Thun M., Linet M.S., Cerhan J.R., Haiman C.A., Schottenfeld D., eds. Cancer Epidemiology and Prevention Fourth edition. Oxford: Oxford University Press; 2018.

Blettner, M., Schlehofer, B., Samkange-Zeeb, F., Berg, G., Schlaefer, K., Schuz, J. Medical exposure to ionising radiation and the risk of brain tumours: Interphone study group, Germany. Eur J Cancer 2007;43:1990-1998.

Boase, J., Ling, R. Measuring mobile phone use: Self-report versus log data. J Comput-Mediat Comm 2013;18:508-519.

Boutron, I., Page, M.J., Higgins, J.P.T., Altman, D.G., Lundh, A., Hróbjartsson, A. Chapter 7: Considering bias and conflicts of interest among the included studies. in: Higgins J.P.T., Thomas J., Chandler J., Cumpston M., Li T., Page M.J., Welch V.A., eds. Cochrane Handbook for Systematic Reviews of Interventions version 6 (updated July 2019): Cochrane; 2019.

Bowling, A. Mode of questionnaire administration can have serious effects on data quality. J Public Health (Oxf) 2005;27:281-291.

Bradley, P.J. Frequency and histopathology by site, major pathologies, symptoms and signs of salivary gland neoplasms. in: Bradley P.J., Eisele D.W., eds. Salivary Gland Neoplasms. Basel: Karger; 2016.

Bradley, P.J., McGurk, M. Incidence of salivary gland neoplasms in a defined UK population. Br J Oral Maxillofac Surg 2013;51:399-403.

Brady, Z., Forsythe, A., McBain-Miller, J., Scurrah, K.J., Smoll, N., Lin, Y., Lee, C., Berrington de Gonzalez, A., Roberts, L.J., Mathews, J.D. CT dosimetry for the Australian Cohort Data Linkage study. Radiat Prot Dosimetry 2020;191:423-438.

Bray, F., Ferlay, J., Soerjomataram, I., Siegel, R.L., Torre, L.A., Jemal, A. Global cancer statistics 2018: GLOBOCAN estimates of incidence and mortality worldwide for 36 cancers in 185 countries. CA Cancer J Clin 2018;68:394-424.

Cabré-Riera, A., Marroun, H.E., Muetzel, R., van Wel, L., Liorni, I., Thielens, A., Birks, L.E., Pierotti, L., Huss, A., Joseph, W., Wiart, J., Capstick, M., Hillegers, M., Vermeulen, R., Cardis, E., Vrijheid, M., White, T., Roosli, M., Tiemeier, H., Guxens, M. Estimated whole-brain and lobe-specific radiofrequency electromagnetic fields doses and brain volumes in preadolescents. Environ Int 2020;142:105808.

Cardis, E., Varsier, N., Bowman, J.D., Deltour, I., Figuerola, J., Mann, S., Moissonnier, M., Taki, M., Vecchia, P., Villegas, R., Vrijheid, M., Wake, K., Wiart, J. Estimation of RF energy absorbed in the brain from mobile phones in the Interphone Study. Occup Environ Med 2011;68:686-693.

Carlberg, M., Söderqvist, F., Hansson Mild, K., Hardell, L. Meningioma patients diagnosed 2007-2009 and the association with use of mobile and cordless phones: a case-control study. Environ Health 2013;12:60.

Catalogue of Bias Collaboration, Spencer, E.A., Brassey, J., Mahtani, K. Recall bias. in: Center for Evidence Based Medicine (CEMB), ed. Catalogue Of Bias (<https://wwwcatalogueofbiasesorg/biases/recall-bias>); 2017.

Checkoway, H., Pearce, N., Crawford-Brown, D.J. eds. Research methods in occupational epidemiology. New York: Oxford University Press; 1989.

Coureau, G., Bouvier, G., Lebailly, P., Fabbro-Peray, P., Gruber, A., Leffondre, K., Guillamo, J.S., Loiseau, H., Mathoulin-Pelissier, S., Salamon, R., Baldi, I. Mobile phone use and brain tumours in the CERENAT case-control study. Occup Environ Med 2014;71:514-522.

Dekkers, O.M., Vandenbroucke, J.P., Cevallos, M., Renehan, A.G., Altman, D.G., Egger, M. COSMOS-E: Guidance on conducting systematic reviews and meta-analyses of observational studies of etiology. PLoS Med 2019;16:e1002742.

Elwood, M. ed. Critical appraisal of epidemiological studies and clinical trials. Oxford: Oxford University Press; 2017.

Fan, Z., Ji, T., Wan, S., Wu, Y., Zhu, Y., Xiao, F., Zhan, R. Smoking and risk of meningioma: a meta-analysis. Cancer Epidemiol 2013;37:39-45.

Feychting, M. Selection and detection bias. in: Röösli M., ed. Epidemiology of Electromagnetic Fields. Boca Raton: CRC Press - Taylor & Francis Group; 2014.

Francis, S.S., Enders, C., Hyde, R., Gao, X., Wang, R., Ma, X., Wiemels, J.L., Selvin, S., Metayer, C. Spatial-temporal cluster analysis of childhood cancer in California. Epidemiology 2020;31:214-223.

Frei, P., Mohler, E., Burgi, A., Frohlich, J., Neubauer, G., Braun-Fahrlander, C., Röösli, M., Qualifex Team. Classification of personal exposure to radio frequency electromagnetic fields (RF-EMF) for epidemiological research: Evaluation of different exposure assessment methods. Environ Int 2010;36:714-720.

Frei, P., Poulsen, A.H., Johansen, C., Olsen, J.H., Steding-Jessen, M., Schuz, J. Use of mobile phones and risk of brain tumours: update of Danish cohort study. BMJ 2011;343:d6387.

Gilbert, E.S., Little, M.P., Preston, D.L., Stram, D.O. Issues in Interpreting Epidemiologic Studies of Populations Exposed to Low-Dose, High-Energy Photon Radiation. J Natl Cancer Inst Monogr 2020;2020:176-187.

Goedhart, G., Kromhout, H., Wiart, J., Vermeulen, R. Validating self-reported mobile phone use in adults using a newly developed smartphone application. Occup Environ Med 2015;72:812-818.

Goedhart, G., van Wel, L., Langer, C.E., de Llobet Viladoms, P., Wiart, J., Hours, M., Kromhout, H., Benke, G., Bouka, E., Bruchim, R., Choi, K.H., Eng, A., Ha, M., Huss, A., Kiyohara, K., Kojimahara, N., Krewski, D., Lacour, B., t Mannetje, A., Maule, M., Migliore, E., Mohipp, C., Momoli, F., Petridou, E.T., Radon, K., Remen, T., Sadetzki, S., Sim, M., Weinmann, T., Cardis, E., Vrijheid, M., Vermeulen, R. Recall of mobile phone usage and laterality in young people: The multinational Mobi-Expo study. Environ Res 2018;165:150-157.

Greaves, M. A causal mechanism for childhood acute lymphoblastic leukaemia. Nat Rev Cancer 2018;18:471-484.

Green-McKenzie, J. Commentary for the Then and Now Forum: The Healthy Worker Effect. J Occup Environ Med 2017;59:335-346.

Greenland, S. Response and follow-up bias in cohort studies. Am J Epidemiol 1977;106:184-187.

Greenland, S. Applications of stratified analysis methods. in: Rothman K.J., Lash T.L., Greenland S., eds. Modern epidemiology. Philadelphia: Lippincot Williams & Wilkins, Wolters Kluwer Health; 2012.

Greenland, S., Lash, T.L. Bias analysis. in: Rothman K.J., Lash T.L., Greenland S., eds. Modern epidemiology. Philadelphia: Lippincot Williams & Wilkins, Wolters Kluwer Health; 2012.

Hardell, L., Carlberg, M., Söderqvist, F., Mild, K.H. Case-control study of the association between malignant brain tumours diagnosed between 2007 and 2009 and mobile and cordless phone use. Int J Oncol 2013;43:1833-1845.

Hareuveny, R., Kavet, R., Shachar, A., Margaliot, M., Kheifets, L. Occupational exposures to radiofrequency fields: results of an Israeli national survey. J Radiol Prot 2015;35:429-445.

Hartge, P. Participation in population studies. Epidemiology 2006;17:252-254.

Hashibe, M., Sturgis, E.M., Ferlay, J., D.M., W. Oral cavity, oropharynx, lip, and salivary glands. in: Thun M., Linet M.S., Cerhan J.R., Haiman C.A., Schottenfeld D., eds. Cancer Epidemiology and Prevention Fourth edition. Oxford: Oxford University Press; 2018.

Heinavaara, S., Tokola, K., Kurttio, P., Auvinen, A. Validation of exposure assessment and assessment of recruitment methods for a prospective cohort study of mobile phone users (COSMOS) in Finland: a pilot study. Environ Health 2011;10:14.

Hernán, M.A. Invited Commentary: Selection bias without colliders. Am J Epidemiol 2017;185:1048-1050.

Hernán, M.A., Hernandez-Diaz, S., Robins, J.M. A structural approach to selection bias. Epidemiology 2004;15:615-625.

Hernán, M.A., Robin, J.R. Causal Inference: What If. Boca Raton: CRC Press; 2020.

Howe, C.J., Cole, S.R., Lau, B., Napravnik, S., Eron, J.J., Jr. Selection Bias Due to Loss to Follow Up in Cohort Studies. Epidemiology 2016;27:91-97.

Hutter, H.P., Ehrenhofer, L., Freuis, E., Hartl, P., Kundi, M. Poor-to-moderate agreement between self and proxy interviews of mobile phone use. Bioelectromagnetics 2012;33:561-567.

IARC. Non-ionizing radiation, Part 2: Radiofrequency electromagnetic fields. IARC Monogr Eval Carcinog Risks Hum, vol 102. Lyon: IARC Press; 2013.

IARC. Benzene. IARC Monogr Eval Carcinog Risks Hum, vol 120. Lyon: IARC Press; 2018.

Infante-Rivard, C., Cusson, A. Reflection on modern methods: selection bias-a review of recent developments. Int J Epidemiol 2018;47:1714-1722.

Interphone Study Group, Cardis, E., Deltour, I., Vrijheid, M., Combalot, E., Moissonnier, M., Tardy, H., Armstrong, B., Giles, G., Brown, J., Siemiatycki, J., Parent, M.E., Nadon, L., Krewski, D., McBride, M.L., Johansen, C., Collatz, C.H., Auvinen, A., Kurttio, P., Lahkola, A., Salminen, T., Hours, M., Bernard, M., Montestruq, L., Schuez, J., Berg-Beckhoff, G., Schlehofer, B., Blettner, M., Sadetzki, S., Chetrit, A., Jarus-Hakak, A., Lagorio, S., Iavarone, I., Takebayashi, T., Yamaguchi, N., Woodward, A., Cook, A., Pearce, N., Tynes, T., Blaasaas, K.G., Klaeboe, L., Feychting, M., Loenn, S., Ahlbom, A., McKinney, P.A., Hepworth, S.J., Muir, K.R., Swerdlow, A.J., Schoemaker, M.J. Brain tumour risk in relation to mobile telephone use: results of the INTERPHONE international case-control study. Int J Epidemiol 2010;39:675-694.

Irestorm, E., Perrin, S., Tonning Olsson, I. Pretreatment cognition in patients diagnosed with pediatric brain tumors. Pediatr Neurol 2018;79:28-33.

ITU. ICT Statistics. Mobile-cellular subscriptions (1980-2018). ICT-Eye website: International Telecommunication Union (<https://www.itu.int/net4/ITU-D/icteye/#/>),; 2020.

Jalilian, H., Eeftens, M., Ziaei, M., Roosli, M. Public exposure to radiofrequency electromagnetic fields in everyday microenvironments: An updated systematic review for Europe. Environ Res 2019;176:108517.

Johansen, C., Schüz, J., Andreasen, A.S., Dalton, S.O. Study designs may influence results: the problems with questionnaire-based case-control studies on the epidemiology of glioma. Br J Cancer 2017;116:841-848.

Joshi, P., Ghasemifard, F., Colombi, D., Tornevik, C. Actual Output Power Levels of User Equipment in 5G Commercial Networks and Implications on Realistic RF EMF Exposure Assessment. IEEE Access 2020;8:204068-204075.

Karipidis, K., Elwood, M., Benke, G., Sanagou, M., Tjong, L., Croft, R.J. Mobile phone use and incidence of brain tumour histological types, grading or anatomical location: a population-based ecological study. BMJ Open 2018;8:e024489.

Karipidis, K., Elwood, M., Benke, G., Sanagou, M., Tjong, L., Croft, R.J. Correction: Mobile phone use and incidence of brain tumour histological types, grading or anatomical location: a population-based ecological study. BMJ Open 2019;9:e024489corr024481.

Kiyohara, K., Wake, K., Watanabe, S., Arima, T., Sato, Y., Kojimahara, N., Taki, M., Cardis, E., Yamaguchi, N. Long-term recall accuracy for mobile phone calls in young Japanese people: A follow-up validation study using software-modified phones. J Expo Sci Environ Epidemiol 2018;28:166-172.

Kobayashi, T., Boase, J. No such effect? The implications of measurement error in self-report measures of mobile communication use. Commun Methods Meas 2012;6:126-143.

Konstantinoudis, G., Kreis, C., Ammann, R.A., Niggli, F., Kuehni, C.E., Spycher, B.D., Swiss Paediatric Oncology, G., the Swiss National Cohort Study, G. Spatial clustering of childhood leukaemia in Switzerland: A nationwide study. Int J Cancer 2017;141:1324-1332.

Kreis, C., Lupatsch, J.E., Niggli, F., Egger, M., Kuehni, C.E., Spycher, B.D., Swiss Paediatric Oncology, G., the Swiss National Cohort Study, G. Space-time clustering of childhood leukemia: Evidence of an association with ETV6-RUNX1 (TEL-AML1) fusion. PLoS One 2017;12:e0170020.

Lapointe, S., Perry, A., Butowski, N.A. Primary brain tumours in adults. Lancet 2018;392:432-446.

Larjavaara, S., Feychting, M., Sankila, R., Johansen, C., Klaeboe, L., Schüz, J., Auvinen, A. Incidence trends of vestibular schwannomas in Denmark, Finland, Norway and Sweden in 1987-2007. Br J Cancer 2011;105:1069-1075.

Lauer, O., Frei, P., Gosselin, M.C., Joseph, W., Roosli, M., Frohlich, J. Combining near- and far-field exposure for an organ-specific and whole-body RF-EMF proxy for epidemiological research: a reference case. Bioelectromagnetics 2013;34:366-374.

Lee, J.W., Wernicke, A.G. Risk and survival outcomes of radiation-induced CNS tumors. J Neurooncol 2016;129:15-22.

Linet, M.S., Morton, L.M., Devesa, S.S., Dores, G.M. Leukemias. in: Thun M., Linet M.S., Cerhan J.R., Haiman C.A., Schottenfeld D., eds. Cancer Epidemiology and Prevention Fourth edition. Oxford: Oxford University Press; 2018.

Linet, M.S., Schubauer-Berigan, M.K., Berrington de Gonzalez, A. Outcome Assessment in Epidemiological Studies of Low-Dose Radiation Exposure and Cancer Risks: Sources, Level of Ascertainment, and Misclassification. J Natl Cancer Inst Monogr 2020;2020:154-175.

Loomis, D.P., Savitz, D.A. Effect of incomplete exposure assessment on epidemiologic dose-response analyses. Scand J Work Environ Health 1994;20:200-205.

Martens, A.L., Slottje, P., Timmermans, D.R.M., Kromhout, H., Reedijk, M., Vermeulen, R.C.H., Smid, T. Modeled and perceived exposure to radiofrequency electromagnetic fields from mobile-phone base stations and the development of symptoms over time in a general population cohort. Am J Epidemiol 2017;186:210-219.

McCarthy, B.J., Kruchko, C., Dolecek, T.A. The impact of the Benign Brain Tumor Cancer Registries Amendment Act (Public Law 107-260) on non-malignant brain and central nervous system tumor incidence trends. J Registry Manag 2013;40:32-35.

McNeill, K.A. Epidemiology of Brain Tumors. Neurol Clin 2016;34:981-998.

Meskal, I., Gehring, K., Rutten, G.J., Sitskoorn, M.M. Cognitive functioning in meningioma patients: a systematic review. J Neurooncol 2016;128:195-205.

Miettinen, O.S. ed. Epidemiological research: terms and concepts: Springer; 2011.

Mireku, M.O., Mueller, W., Fleming, C., Chang, I., Dumontheil, I., Thomas, M.S.C., Eeftens, M., Elliott, P., Roosli, M., Toledano, M.B. Total recall in the SCAMP cohort: Validation of self-reported mobile phone use in the smartphone era. Environ Res 2018;161:1-8.

Morgan, R.W., Kelsh, M.A., Zhao, K., Exuzides, K.A., Heringer, S., Negrete, W. Radiofrequency exposure and mortality from cancer of the brain and lymphatic/hematopoietic systems. Epidemiology 2000;11:118-127.

Naimi, A.I., Richardson, D.B., Cole, S.R. Causal inference in occupational epidemiology: accounting for the healthy worker effect by using structural nested models. Am J Epidemiol 2013;178:1681-1686.

National IT and Telecom Agency. Tele yearbook. Copenhagen: National IT and Telecom Agency; 2001.

NTP-OHAT. OHAT Risk of Bias Rating Tool for Human and Animal Studies. National Toxicology Program - Office of Health Assessment and Translation; 2015.

NTP-OHAT. Handbook for Conducting a Literature-Based Health Assessment Using OHAT Approach for Systematic Review and Evidence Integration (March 4, 2019). National Toxicology Program - Office of Health Assessment and Translation; 2019.

NTP-ORoC. Handbook for Preparing Report on Carcinogens Monographs. Durham: National Toxicology Program - Office of the Report on Carcinogens; 2015.

Olsson, A., Bouaoun, L., Auvinen, A., Feychting, M., Johansen, C., Mathiesen, T., Melin, B., Lahkola, A., Larjavaara, S., Villegier, A.S., Byrnes, G., Deltour, I., Schuz, J. Survival of glioma patients in relation to mobile phone use in Denmark, Finland and Sweden. J Neurooncol 2019;141:139-149.

Ostrom, Q.T., Adel Fahmideh, M., Cote, D.J., Muskens, I.S., Schraw, J.M., Scheurer, M.E., Bondy, M.L. Risk factors for childhood and adult primary brain tumors. Neuro Oncol 2019;21:1357-1375.

Ostrom, Q.T., Patil, N., Cioffi, G., Waite, K., Kruchko, C., Barnholtz-Sloan, J.S. CBTRUS Statistical Report: Primary Brain and Other Central Nervous System Tumors Diagnosed in the United States in 2013-2017. Neuro Oncol 2020;22:iv1-iv96.

Persson, T., Tornevik, C., Larsson, L.E., Loven, J. Output power distributions of terminals in a 3G mobile communication network. Bioelectromagnetics 2012;33:320-325.

Pettersson, D., Mathiesen, T., Prochazka, M., Bergenheim, T., Florentzson, R., Harder, H., Nyberg, G., Siesjo, P., Feychting, M. Long-term mobile phone use and acoustic neuroma risk. Epidemiology 2014;25:233-241.

Pinsky, P.F., Yu, K., Black, A., Huang, W.Y., Prorok, P.C. Active follow-up versus passive linkage with cancer registries for case ascertainment in a cohort. Cancer Epidemiol 2016;45:26-31.

Porta, M. ed. A Dictionary of Epidemiology. Sixth edition, ebook. Oxford: Oxford University Press; 2016.

Reznitsky, M., Petersen, M., West, N., Stangerup, S.E., Caye-Thomasen, P. Epidemiology of vestibular schwannomas - prospective 40-year data from an unselected national cohort. Clin Epidemiol 2019;11:981-986.

Roman, E., Smith, A.G. Leukaemias. Understanding pathogenesis through similarities and differences. in: Wild C.P., Weiderpass E., B.W. S., eds. World Cancer Report: Cancer Research for Cancer Prevention. Lyon: IARC Press; 2020.

Röösli, M., Lagorio, S., Schoemaker, M.J., Schüz, J., Feychting, M. Brain and salivary gland tumors and mobile phone use: Evaluating the evidence from various epidemiological study designs. Annu Rev Public Health 2019;40:221-238.

Roth, N., Wilks, M.F. Neurodevelopmental and neurobehavioural effects of polybrominated and perfluorinated chemicals: a systematic review of the epidemiological literature using a quality assessment scheme. Toxicol Lett 2014;230:271-281.

Rowley, J.T., Joyner, K.H. Comparative international analysis of radiofrequency exposure surveys of mobile communication radio base stations. J Expo Sci Environ Epidemiol 2012;22:304-315.

Savitz, D.A., Wellenius, G.A. eds. Interpreting Epidemiologic Evidence: Connecting Research to Applications. Oxford: Oxford University Press; 2016.

SCENIHR ed. Health Effects of Exposure to EMF. Brussels: European Commission's Scientific Committee on Emerging and Newly Identified Health Risks; 2009.

SCENIHR. Potential health effects of exposure to electromagnetic fields (EMF). Luxembourg: European Commission's Scientific Committee on Emerging and Newly Identified Health Risks; 2015.

Schmiedel, S., Bruggemeyer, H., Philipp, J., Wendler, J., Merzenich, H., Schüz, J. An evaluation of exposure metrics in an epidemiologic study on radio and television broadcast transmitters and the risk of childhood leukemia. Bioelectromagnetics 2009;30:81-91.

Schneider, A.B., Ron, E., Lubin, J., Stovall, M., Shore-Freedman, E., Tolentino, J., Collins, B.J. Acoustic neuromas following childhood radiation treatment for benign conditions of the head and neck. Neuro Oncol 2008;10:73-78.

Schüz, J., Erdmann, F. Environmental exposure and risk of childhood leukemia: An overview. Arch Med Res 2016;47:607-614.

Schüz, J., Johansen, C. A comparison of self-reported cellular telephone use with subscriber data: agreement between the two methods and implications for risk estimation. Bioelectromagnetics 2007;28:130-136.

Schüz, J., Waldemar, G., Olsen, J.H., Johansen, C. Risks for central nervous system diseases among mobile phone subscribers: a Danish retrospective cohort study. PLoS One 2009;4:e4389.

Shao, C., Zhao, W., Qi, Z., He, J. Smoking and glioma risk: Evidence from a meta-analysis of 25 observational studies. Medicine (Baltimore) 2016;95:e2447.

Shapiro, A.J., Antoni, S., Guyton, K.Z., Lunn, R.M., Loomis, D., Rusyn, I., Jahnke, G.D., Schwingl, P.J., Mehta, S.S., Addington, J., Guha, N. Software tools to facilitate systematic review used for cancer hazard identification. Environ Health Perspect 2018;126:104501.

Smoll, N.R., Mathews, J.D., Scurrah, K.J. CT scans in childhood predict subsequent brain cancer: Finite mixture modelling can help separate reverse causation scans from those that may be causal. Cancer Epidemiol 2020;67:101732.

Steenland, K., Schubauer-Berigan, M.K., Vermeulen, R., Lunn, R.M., Straif, K., Zahm, S., Stewart, P., Arroyave, W.D., Mehta, S.S., Pearce, N. Risk of bias assessments and evidence syntheses for observational epidemiologic studies of environmental and occupational exposures: Strengths and limitations. Environ Health Perspect 2020;128:95002.

Sterne, J.A.C., Hernán, M.A., McAleenan, A., Reeves, B.C., Higgins, J.P.T. Chapter 25. Assessing risk of bias in a non-randomized study. in: Higgins J.P.T., Thomas J., Chandler J., Cumpston M., Li T., Page M.J., Welch V.A., eds. Cochrane Handbook for Systematic Reviews of Interventions version 6 (updated July 2019): Cochrane; 2019.

Sterne, J.A.C., Higgins, J.P.T., Reeves, B.C., on behalf of the development group for ACROBAT-NRSI. Cochrane Risk of Bias Assessment Tool: for Non-Randomized Studies of Interventions (ACROBAT-NRSI), Version 1.0.0. 2014.

Tettamanti, G., Ljung, R., Ahlbom, A., Talback, M., Lannering, B., Mathiesen, T., Segerlind, J.P., Feychting, M. Central nervous system tumor registration in the Swedish Cancer Register and Inpatient Register between 1990 and 2014. Clin Epidemiol 2019;11:81-92.

Toledano, M.B., Auvinen, A., Tettamanti, G., Cao, Y., Feychting, M., Ahlbom, A., Fremling, K., Heinavaara, S., Kojo, K., Knowles, G., Smith, R.B., Schuz, J., Johansen, C., Poulsen, A.H., Deltour, I., Vermeulen, R., Kromhout, H., Elliott, P., Hillert, L. An international prospective cohort study of mobile phone users and health (COSMOS): Factors affecting validity of self-reported mobile phone use. Int J Hyg Environ Health 2018;221:1-8.

Udaka, Y.T., Packer, R.J. Pediatric brain tumors. Neurol Clin 2018;36:533-556.

van Kessel, E., Baumfalk, A.E., van Zandvoort, M.J.E., Robe, P.A., Snijders, T.J. Tumor-related neurocognitive dysfunction in patients with diffuse glioma: a systematic review of neurocognitive functioning prior to anti-tumor treatment. J Neurooncol 2017;134:9-18.

van Loon, E.M., Heijenbrok-Kal, M.H., van Loon, W.S., van den Bent, M.J., Vincent, A.J., de Koning, I., Ribbers, G.M. Assessment methods and prevalence of cognitive dysfunction in patients with low-grade glioma: A systematic review. J Rehabil Med 2015;47:481-488.

van Smeden, M., Lash, T.L., Groenwold, R.H.H. Reflection on modern methods: five myths about measurement error in epidemiological research. Int J Epidemiol 2020;49:338-347.

van Wel, L., Liorni, I., Huss, A., Thielens, A., Wiart, J., Joseph, W., Roosli, M., Foerster, M., Massardier-Pilonchery, A., Capstick, M., Cardis, E., Vermeulen, R. Radio-frequency electromagnetic field exposure and contribution of sources in the general population: an organ-specific integrative exposure assessment. J Expo Sci Environ Epidemiol 2021;epub 2021/03/02

VanderWeele, T.J. Principles of confounder selection. Eur J Epidemiol 2019;34:211-219.

Verbeek, J., Oftedal, G., Feychting, M., van Rongen, E., Rosaria Scarfi, M., Mann, S., Wong, R., van Deventer, E. Prioritizing health outcomes when assessing the effects of exposure to radiofrequency electromagnetic fields: A survey among experts. Environ Int 2021;146:106300.

Vergnaud, A.C., Aresu, M., McRobie, D., Singh, D., Spear, J., Heard, A., Elliott, P. Validation of objective records and misreporting of personal radio use in a cohort of British Police forces (the Airwave Health Monitoring Study). Environ Res 2016;148:367-375.

Vila, J., Turner, M.C., Gracia-Lavedan, E., Figuerola, J., Bowman, J.D., Kincl, L., Richardson, L., Benke, G., Hours, M., Krewski, D., McLean, D., Parent, M.E., Sadetzki, S., Schlaefer, K., Schlehofer, B., Schuz, J., Siemiatycki, J., van Tongeren, M., Cardis, E., Group, I.S. Occupational exposure to high-frequency electromagnetic fields and brain tumor risk in the INTEROCC study: An individualized assessment approach. Environ Int 2018;119:353-365.

Viswanathan, M., Ansari, M.T., Berkman, N.D., Chang, S., Hartling, L., McPheeters, M., Santaguida, P.L., Shamliyan, T., Singh, K., Tsertsvadze, A., Treadwell, J.R. AHRQ Methods for Effective Health Care. Assessing the Risk of Bias of Individual Studies in Systematic Reviews of Health Care Interventions. AHRQ Publication No. 12-EHC047-EF. Methods Guide for Effectiveness and Comparative Effectiveness Reviews. Rockville: Agency for Healthcare Research and Quality; 2012.

Vrijheid, M., Armstrong, B.K., Bedard, D., Brown, J., Deltour, I., Iavarone, I., Krewski, D., Lagorio, S., Moore, S., Richardson, L., Giles, G.G., McBride, M., Parent, M.E., Siemiatycki, J., Cardis, E. Recall bias in the assessment of exposure to mobile phones. J Expo Sci Environ Epidemiol 2009;19:369-381.

Vrijheid, M., Cardis, E., Armstrong, B.K., Auvinen, A., Berg, G., Blaasaas, K.G., Brown, J., Carroll, M., Chetrit, A., Christensen, H.C., Deltour, I., Feychting, M., Giles, G.G., Hepworth, S.J., Hours, M., Iavarone, I., Johansen, C., Klaeboe, L., Kurttio, P., Lagorio, S., Lonn, S., McKinney, P.A., Montestrucq, L., Parslow, R.C., Richardson, L., Sadetzki, S., Salminen, T., Schuz, J., Tynes, T., Woodward, A., Interphone Study Group. Validation of short term recall of mobile phone use for the Interphone study. Occup Environ Med 2006;63:237-243.

Wall, S., Wang, Z.M., Kendig, T., Dobraca, D., Lipsett, M. Real-world cell phone radiofrequency electromagnetic field exposures. Environ Res 2019;171:581-592.

Wang, Z., Taylor, K., Allman-Farinelli, M., Armstrong, B., Askie, L., Ghersi, D., McKenzie, J., Norris, S.L., Page, M.J., Rooney, A., Woodruff, T., Bero, L.A. A systematic review: Tools for assessing methodological quality of human observational studies. . MetaArXiv. Created: May 22, 2019 | Last edited: July 18, 2020 NHMRC; 2020.

Whaley, P., Aiassa, E., Beausoleil, C., Beronius, A., Bilotta, G., Boobis, A., de Vries, R., Hanberg, A., Hoffmann, S., Hunt, N., Kwiatkowski, C.F., Lam, J., Lipworth, S., Martin, O., Randall, N., Rhomberg, L., Rooney, A.A., Schünemann, H.J., Wikoff, D., Wolffe, T., Halsall, C. Recommendations for the conduct of systematic reviews in toxicology and environmental health research (COSTER). Environ Int 2020;143

Woodruff, T.J., Sutton, P. The Navigation Guide systematic review methodology: a rigorous and transparent method for translating environmental health science into better health outcomes. Environ Health Perspect 2014;122:1007-1014.

World Cancer Report. Known causes of human cancer by organ site. in: Wild C.P., Weiderpass E., B.W. S., eds. World Cancer Report: Cancer Research for Cancer Prevention. Lyon: IARC Press; 2020.

Yamanaka, R., Hayano, A., Kanayama, T. Radiation-induced meningiomas: An exhaustive review of the literature. World Neurosurg 2017;97:635-644 e638.

Yamanaka, R., Hayano, A., Kanayama, T. Radiation-induced gliomas: A comprehensive review and meta-analysis. Neurosurg Rev 2018;41:719-731.

Zhan, K.Y., Khaja, S.F., Flack, A.B., Day, T.A. Benign Parotid Tumors. Otolaryngol Clin North Am 2016;49:327-342.

1. List obtained via the [interactive evidence map](https://ntp.niehs.nih.gov/whatwestudy/assessments/noncancer/tools/index1.html?utm_source=direct&utm_medium=prod&utm_campaign=ntpgolinks&utm_term=ohat_tools) of the risk-of-bias tools reviewed by Wang et al. (2019), available from the NTP web site (last accessed 2021/05/25). [↑](#footnote-ref-1)
2. Centre for Research Synthesis and Decision Analysis, University of Bristol, UK: [ROBINS-E](https://www.bristol.ac.uk/population-health-sciences/centres/cresyda/barr/riskofbias/robins-e/) (last accessed 2021/05/25) [↑](#footnote-ref-2)
