# Supplementary material for: The effect of exposure to radiofrequency fields on cancer risk in the general and working population: A protocol for a systematic review of human observational studies
Source: Environ Int. 2021 Dec;157:106828. doi: 10.1016/j.envint.2021.106828 (PMC8484862; doi:10.1016/j.envint.2021.106828)

**Table 1. Key Study Characteristics Table template for cohort studies**

**Table X_1-n_** (grouped and ordered by exposure source and neoplasm)

| **Reference** | **Country,**  **Time period**  **(enrolment)** | **Cohort definition (source population)** | **Reference**  **group** | **No. individuals (E^+^/E^-^), age range** | **Follow-up period, rates, duration (mean, range)** | **PYAR**  **(E^+^/E^-^)** | **Case ascertainment, Outcome definition (ICD-10 \| ICD-O)** | **Exposure assessment** | **Exposure metrics** |
| --- | --- | --- | --- | --- | --- | --- | --- | --- | --- |
|  |  |  |  |  |  |  |  |  |  |
|  |  |  |  |  |  |  |  |  |  |
|  |  |  |  |  |  |  |  |  |  |
|  |  |  |  |  |  |  |  |  |  |
|  |  |  |  |  |  |  |  |  |  |
|  |  |  |  |  |  |  |  |  |  |
|  |  |  |  |  |  |  |  |  |  |
|  |  |  |  |  |  |  |  |  |  |

**Table 2. Key Study Characteristics Table template for case-control studies**

**Table Y_1-n_** (grouped and ordered by exposure source and neoplasm): **Case-control studies**

| **Reference** | **Country,**  **Time period**  **(diagnosis)** | **No. cases/controls, Age range** | **Case ascertainment, Outcome definition**  **(ICD-10 \| ICD-O)** | **Control type, source, selection** | **Matching variables and procedure (if applicable)** | **Participation rates (cases and controls)** | **Exposure assessment** | **Exposure metrics** |
| --- | --- | --- | --- | --- | --- | --- | --- | --- |
|  |  |  |  |  |  |  |  |  |
|  |  |  |  |  |  |  |  |  |
|  |  |  |  |  |  |  |  |  |
|  |  |  |  |  |  |  |  |  |
|  |  |  |  |  |  |  |  |  |
|  |  |  |  |  |  |  |  |  |
|  |  |  |  |  |  |  |  |  |
|  |  |  |  |  |  |  |  |  |

**Table 3. Summary of Finding Table template**

**Table Z_1-n_** (grouped and ordered by exposure source, neoplasm, and study design)

| **Study ID (reference)** | **Design** | **Exposure** | | **No. E^+^ cases** | **RR ( 95% CI)** | **Statistical method** | **Covariates in the analyses** |
| --- | --- | --- | --- | --- | --- | --- | --- |
|  |  | **Metric** | **Level** |  |  |  |  |
|  |  |  |  |  |  |  |  |
|  |  |  |  |  |  |  |  |
|  |  |  |  |  |  |  |  |
|  |  |  |  |  |  |  |  |
|  |  |  |  |  |  |  |  |
|  |  |  |  |  |  |  |  |
|  |  |  |  |  |  |  |  |
|  |  |  |  |  |  |  |  |

**Figure 1. Example of an Effect Direction Plot: Findings from case-control studies of glioma risk by cumulative call time**


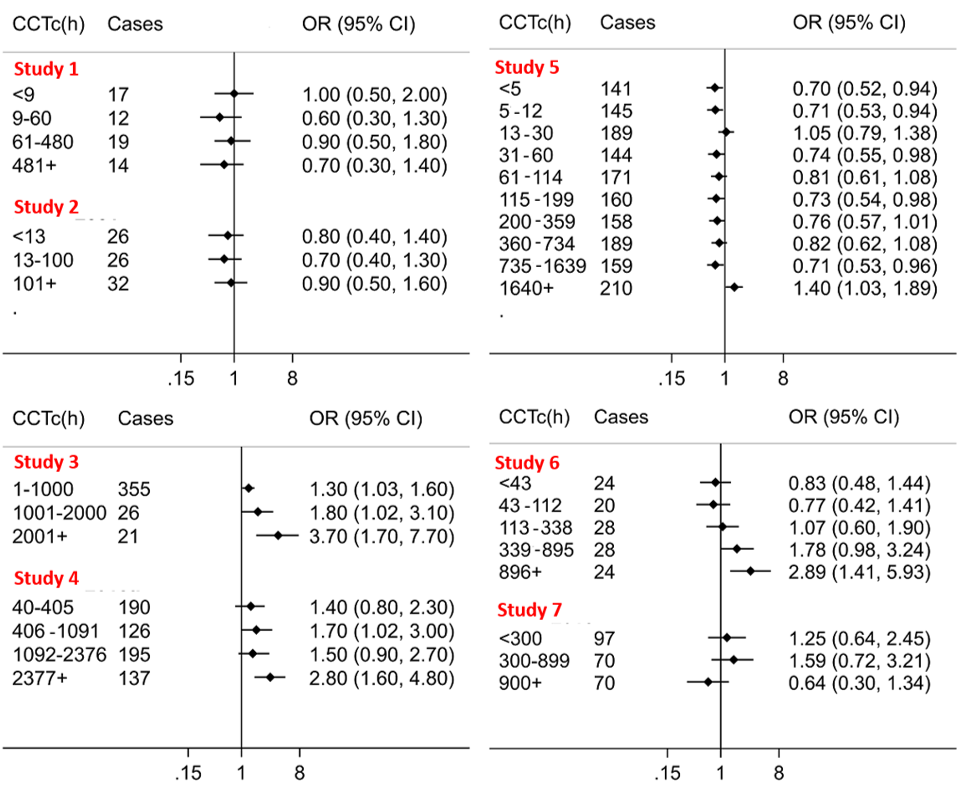

Supplement: Supplementary data 5 [file mmc5.docx]
