# Supplementary material for: The effect of exposure to radiofrequency fields on cancer risk in the general and working population: A protocol for a systematic review of human observational studies
Source: Environ Int. 2021 Dec;157:106828. doi: 10.1016/j.envint.2021.106828 (PMC8484862; doi:10.1016/j.envint.2021.106828)
Supplement: Supplementary data 6 [file mmc6.docx]

Systematic review of human observational studies on the effect of exposure
to radiofrequency fields and cancer risk in the general and working population

**Protocol for the assessment of confidence in the evidence**

**Introduction**

To assess confidence that the study findings accurately reflect the true exposure-effect association (conventionally referred to as certainty of the evidence or quality of the body of evidence), we will follow the approach developed by the National Toxicology Program - Office of Health Assessment and Translation (NTP-OHAT 2019; Rooney et al. 2014). The choice of the method was based on the considerations below.

The systematic review (SR) planned in the current protocol is one of a series commissioned by the World Health Organization (WHO) in the framework of an ongoing project to assess potential health effects of exposure to radiofrequency electromagnetic fields (RF-EMF) in the general and working population (Verbeek et al. 2021).

The WHO recommended a harmonized approach to the assessment of the quality of evidence, in line with the requirements formulated in the WHO Handbook for Guideline Development (WHO 2014), which endorses the Grading of Recommendations Assessment, Development and Evaluation (GRADE) method.

In the current version of GRADE, the bodies of evidence provided by randomized controlled trials (RCT) and non-randomized studies (NRS) of health care interventions are assigned an initial rating of high and low quality, respectively; the certainty of evidence can be lowered based on five domains: risk-of-bias (assessed using congruent tools, such as ROB 2 for RCT and ROBIN-I for NRS in Cochrane systematic reviews), inconsistency, indirectness (or inapplicability), imprecision, and publication bias; the evidence from NRS (rarely from RCT) can be upgraded for large magnitude of effect, dose-response gradient, and opposing residual bias and confounding (Schünemann et al. 2019; Schünemann et al. 2021).

We share the convincement that the strength of GRADE rests on the use of a structured and transparent framework for the assessment of the quality of the evidence (WHO 2014). We also note the importance of a common lexicon, used internationally by over 100 organizations, to classify the level of evidence and formulate conclusions about “what works in health care” (Eden et al. 2011). A similar alignment of currently disparate approaches to the assessment of the certainty and strength of evidence in the context of environmental hazard/risk assessments, would enhance risk communication and its impact on risk perception.

We echo WHO in noting that the GRADE approach for assessing the quality of evidence required to answer questions regarding environmental exposures is under development [(WHO 2014), p. 121; (Morgan et al. 2019; Morgan et al. 2016)], and no consensus on the topic has been reached to date.

The COSTER (Conduct of Systematic Reviews in Toxicology and Environmental Health Research) working group recommends that “the assessment of overall confidence in the evidence included in a SR cover seven characteristics: internal validity, consistency, precision, magnitude of effect, dose-response relationship, reporting and publication bias, and external validity”; it adds that “while these are the same broad characteristics as those utilised in the GRADE framework, COSTER makes no specific recommendations about which tool should be used for assessing these characteristics nor how they should be interpreted, except that the approach should be described in the SR protocol” (Whaley et al. 2020).

The authors of COSMOS-E (Guidance on conducting systematic reviews and meta-analyses of observational studies of etiology) acknowledge that the GRADE system can be helpful to formally judge the extent of confidence that the estimates of an effect are adequate to support a particular decision or recommendation, taking into account study design, risk of bias, degree of inconsistency, imprecision and indirectness (applicability) of results, and reporting bias (Dekkers et al. 2019). Concurrently, they underline the value of triangulation (Lawlor et al. 2016) in causal inference because, if different approaches all point to the same conclusion, this strengthens confidence that the finding may be causal (Dekkers et al. 2019).

A cohesive and non-algorithmic approach to the appraisal of evidence from epidemiological studies in environmental and occupational health is advocated by several scientists and entities (Arroyave et al. 2021; NTP-ORoC 2015; Samet et al. 2020; Savitz et al. 2019; Steenland et al. 2020). The a-priori downgrading of human observational studies is considered the most challenging feature of evidence assessement methods adapted from clinical epidemiology, because the cross-sectional, case-control, or cohort design may be the only feasible or ethical option to provide evidence on environmental health hazards and preventable causes of diseases (Arroyave et al. 2021; Morgan et al. 2016; Steenland et al. 2020).

On the other hand, there are also compelling reasons in favour of a ranking of epidemiological study designs for assessing cause-effect relationship. For example, in aetiological investigations of cancer (especially glioma and other central nervous system neoplasms), questionnaire-based case-control studies are more prone to exposure-information and selection biases, and therefore less informative, than cohort studies (Johansen et al. 2017).

Various adaptions of GRADE have been developed (Héroux and Verbeek 2018; Rooney et al. 2014; Thayer and Schünemann 2016; Woodruff and Sutton 2014) and tested in several SRs of epidemiological studies on environmental hazards (Clark and Paunovic 2018; Johnson et al. 2016; Johnson et al. 2014; Kempen et al. 2018; Lam et al. 2017; Nieuwenhuijsen et al. 2017; NTP 2016; 2019a; 2019b; 2019c; Vesterinen et al. 2017; WHO 2018).

In one approach, the starting point for the quality of evidence assessment was based on the study design most applicable and available from any specific research fields (Héroux and Verbeek 2018). This resulted in a different baseline rating assigned to the same study design, depending on the investigated effect (WHO 2018).

Relying on the best available or achievable rather than least biased evidence as a reference standard, would lead to differing certainty in decisions based on the questions asked (Katikireddi et al. 2015; Schünemann et al. 2019). We considered this adaptation of GRADE unsuitable to a systematic review of the evidence about multiple health hazards from several types/sources/settings of exposure to RF-EMF, because changes in the rating scale across lines of evidence would compromise the internal coherence of the appraisal and impair risk communication.

In the Navigation Guide’s approach (Woodruff and Sutton 2014), the body of human observational evidence is assigned an initial rating of “moderate” quality, independent of the specifics of the studies in the assessment. This appears an outward rather than a substantial change.

The evidence appraisal method developed by OHAT in the framework of a comprehensive approach to hazard assessments based on systematic reviews of the scientific literature (NTP-OHAT 2019; Rooney et al. 2014; Rooney et al. 2016), is free from the aforementioned drawbacks.

Compared to the original GRADE system, the major change introduced by OHAT is a four-level initial confidence rating (high, moderate, low, and very low), based on the number of favourable features in the study design (controlled exposure, exposure prior to outcome, individual outcome data, presence of a comparison group), consistently applied across evidence streams (human, animal and cell studies).

An additional difference is a fourth upgrading domain: consistency across study designs and populations (for human studies), or across multiple species and models (for animal studies). This makes it possible to fully exploit findings from analyses of the sources of heterogeneity in results across studies, lessening confidence in the evidence in the presence of substantial unexplained inconsistency, and increasing it for consistency not attributable to bias or other dissuading concerns (NTP 2019a; 2019c).

Our choice of the OHAT’s GRADE adaptation was also motivated by issues of methodological congruity between interrelated steps of the systematic review. The first domain considered in the GRADE framework is the risk-of-bias (RoB) across studies contributing to a body of evidence. This summary measure is obtained elaborating findings from the assessment of each study’s proneness to various sources of biased measures of effect. In our planned systematic review, the study’s internal validity will be assessed using a customized version of the OHAT RoB tool, and we deemed appropriate relying on a consistent method for the assessment of RoB across studies.

At the request of WHO, the OHAT’s extra-upgrading domain will not be considered in our assessment. For the same reason, we will not translate the confidence rating into level of evidence for health effect or for no health effect [Step 6 of the OHAT approach (NTP-OHAT 2019)].

**Methods**

We will assess confidence in the body of evidence per outcome, by category of exposure addressed in each component of our systematic review (SR-A, SR-B, SR-C), and across multiple exposure types and related endpoints. In line with the MECIR standard C14 (Higgins et al. 2020), the assessment will focus on six critical outcomes (see § 3.1.4 of the protocol): brain tumours (including gliomas and other histotypes); meningioma; acoustic neuroma; pituitary tumours; salivary gland tumours; and leukaemias (including several subtypes).

The level of confidence in the exposure-outcome association will be indicated according to four descriptors:

| **High Confidence (++++)**: | The true effect is highly likely to be reflected in the apparent relationship. |
| --- | --- |
| **Moderate Confidence (+++)**: | The true effect may be reflected in the apparent relationship. |
| **Low Confidence (++)**: | The true effect may be different from the apparent relationship. |
| **Very Low Confidence (+)**: | The true effect is highly likely to be different from the apparent relationship. |

The assessment process and related decision rules are described below.

1. **Initial rating by study design features**

All included studies will be given an initial confidence rating based on key study design features:

- *controlled exposure* (the exposure to the substance is experimentally controlled);
- *exposure prior to outcome* (the exposure assessment demonstrates that exposures occurred prior to the development of the outcome, or concurrent with aggravation/amplification of an existing condition);
- *individual outcome data* (the outcome is assessed on the individual level, i.e., not through population aggregate data);
- *comparison group used* (an appropriate comparison group is included in the study).

These study design features are distinct from the risk of bias across studies (addressed later on).

Compliance with all four requirements is necessary for an initial “high confidence” rating.

As there will be no controlled-exposure study in the review dataset, and all other requirements are in principle met by the study designs eligible for inclusion (cohort, case-control, and variants thereof), all included studies will receive an initial rating of “moderate” confidence.

1. **Confidence rating**
   1. *Downgrading and upgrading factors*

The initial rating may be downgraded for factors that decrease confidence in the results: summary risk of bias (across studies); unexplained inconsistency; indirectness or lack of applicability; imprecision; publication bias. The rating may be upgraded for factors that increase confidence in the results: large magnitude of effect; dose response; residual confounding or other factors counter to the observed effect.

Downgrading factors are assessed in degrees (not serious; serious; very serious), except for publication bias (detected or not detected). Upgrading factors are evaluated in terms of occurrence (yes/no), except for magnitude of effect (large or not large).

When a decision to downgrade is borderline for two domains, the body of evidence is downgraded once in a single domain; similarly, the body of evidence is not upgraded twice for the same asset eventually applicable to more than one domain (NTP-OHAT 2019). For example, wide confidence intervals of meta-risk estimates may be due to unexplained inconsistency (statistical heterogeneity of results across studies) or to imprecision (low power of the studies); downgrading twice will only be considered appropriate if the studies show a “very serious” degree of both inconsistency and imprecision.

- 1. *Decision rules*

We will apply the following criteria in deciding whether to downgrade or upgrade confidence in the evidence per outcome by exposure-specific line of evidence.

Risk of bias across studies

The relevant information consists of the visual summaries of the risk of bias (Figure 1), and the results of the 3-level tiering of study quality examined across studies (see § 3.5.2 of the SR protocol).

**Figure 1. Example of summary risk of bias visual display (RoB heat map)**


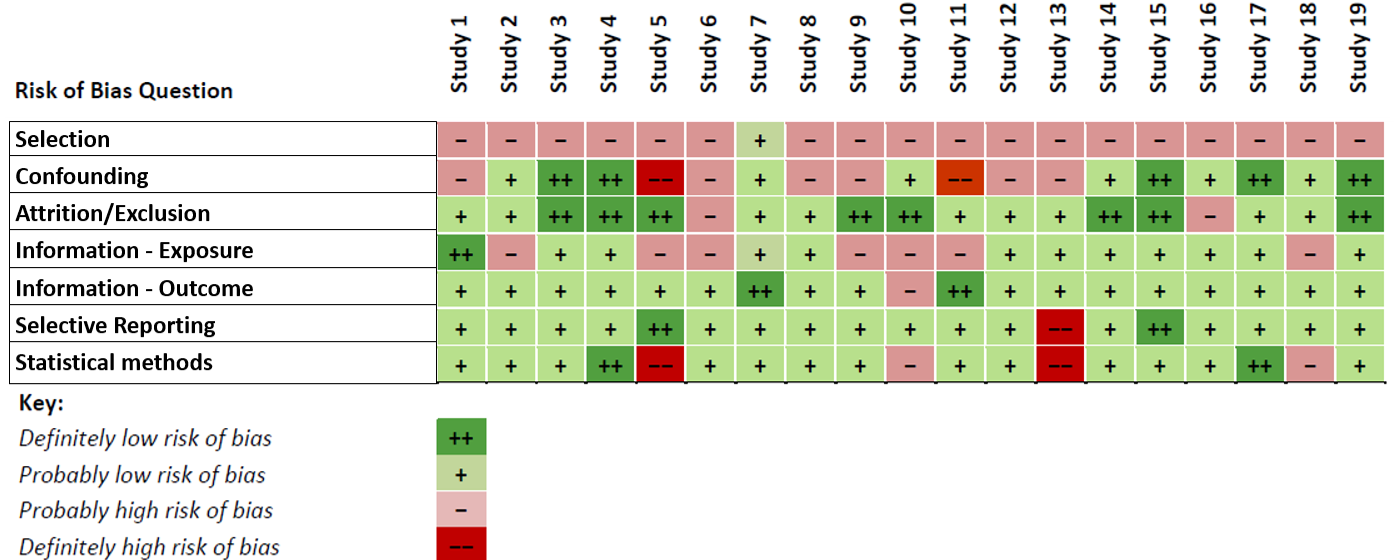


In deciding whether to downgrade for risk-of-bias in the dataset, we will apply the conservative criteria suggested by OHAT: no downgrade if most information is from tier-1 studies (plausible bias unlikely to seriously alter the results), or from tier-1 and tier-2 studies (plausible bias that raises some doubt about the results); downgrade 1 level if most information is from tier-3 studies (plausible bias that seriously weakens confidence in the results).

Unexplained inconsistency

Confidence in the body of evidence will be reduced in the presence of unexplained inconsistency, defined as a large variability in the direction or magnitude of individual study effect estimates for comparable measures of association, not attributable to study design features such as timing of exposure or health outcome assessment.

If differences in risk of bias explain the heterogeneity of findings, we will reconsider the decision on whether or not to downgrade for risk of bias in developing the confidence rating because, in such case, the evidence assessment may focus on the less biased body of evidence.

As no single measure of consistency is ideal, we will determine whether to downgrade for inconsistency taking into account all the following factors: (1) similarity of point estimates, (2) extent of overlap between confidence intervals and, if a meta-analysis is feasible, (3) results of statistical tests of heterogeneity (Cochran’s Q and I^2^).

Unexplained inconsistency will then be rated as:

- “Not serious”, when: point estimates are similar; confidence intervals overlap; statistical heterogeneity is non-significant (p ≥0.1); I^2^ ≤50%.
- “Serious”, when: point estimates vary; confidence intervals show minimal overlap; statistical heterogeneity has low p-value (p ≤0.1); I^2^ of >50% to 75%.
- “Very serious”, when: point estimates vary widely; confidence intervals show minimal or no overlap; statistical heterogeneity has low p-value (p ≤0.1); I^2^ > 75%.

Confidence in the body of evidence will be downgraded 1 level for serious/very serious inconsistency.

In cases where the evidence base consists of a single study, consistency will be rated as “unknown”, and documented as such in the summary of evidence table.

Indirectness

In line with OHAT guidance, we will not downgrade for indirectness the evidence from human observational studies (regardless of the exposure level or setting), unless the body of evidence mostly consists of studies of too short duration of exposure or follow-up to detect potential adverse effect (as presumed based on ancillary evidence about the natural history of the disease and latency estimates related to established risk factors).

Imprecision

We will use 95% confidence intervals as the primary method to assess imprecision, defined as the degree of certainty surrounding an effect estimate with respect to a given outcome. We will rate imprecision as “not serious” when, for most studies or the summary finding from a meta-analysis, the ratio of the upper to lower 95% confidence limit (CL ratio) of the relative risk (RR) estimate is <10. Imprecision will be rated as “very serious” when the CL ratio is ≥10. In the latter instance, confidence in the body of evidence will be downgraded 1 level.

Publication bias

We will rate publication bias as “non-detected” (no downgrade), or “strongly suspected”. Publication bias will be assessed based on funnel plot and Egger’s test (with the caveats noted in § 3.5.3 of the SR protocol). To assess small study effects often due to early positive studies, we will check the pattern of findings over time through the cumulative meta-analyses by publication date mentioned in § 3.7.2 of the SR protocol. We will also evaluate findings by funding sources, according to the level of concern for their influence on the study conduct and reporting. We will consider at *no-to-slight concern* studies financed by public funds or by a firewall model, including an explicit statement that the funder had no role in the design, conduct and reporting of the study. A high prevalence (tentatively >50%) of studies not complying with the above requirement might be considered indicative of publication bias from selective reporting due to the funding source. An important caveat is that newer studies may appear to be at greater risk than older studies because of changes in journal reporting standards on funding and conflicts of interests [(NTP-OHAT 2019), p. 40].

Downgrading (1 level) will be reserved for cases where the concern for publication bias is serious enough to significantly reduce confidence in the body of evidence.

Magnitude of effect

We will determine whether the magnitude of the effect is large based on the background incidence rate of each neoplasm of interest, and the exposure pattern in human studies (including intensity and duration).

We will not rate up for a large effect size if we have major concerns about other issues, especially risk of bias (with a focus on differential exposure measurement error for self-reported variables collected after diagnosis).

Dose-response

We will upgrade for evidence of a monotonic dose-response gradient, and also for evidence of a non-monotonic dose response when this is consistently observed in the evidence base, as there are no a priori hypotheses about the expected shape of the exposure-response relation investigated in our systematic reviews. When a quantitative synthesis of data is feasible, we will evaluate exposure-response gradients through dose-response meta-analysis (see § 3.6.1 of the SR protocol). Otherwise, the assessment will rely on visual inspection of data (summary of finding tables or effect direction plots) sorted by exposure levels. Again, we will not rate up for dose-response if we have major concerns about risk of bias (especially about systematic and differential exposure measurement errors affecting self-reported variables collected after diagnosis).

Residual confounding or other factors counter to the observed effect

Residual confounding refers to consideration of unmeasured determinants of an outcome unaccounted for in an adjusted analysis, that are likely to be distributed unequally across groups (Guyatt et al. 2011). Confidence in the association is increased when a study reports an exposure-disease association despite the presence of residual confounding and the expected impact of the unmeasured (or inappropriately measured) confounder is opposite to the observed effect. We will consider upgrading when there are indications that residual confounding or bias would underestimate an apparent association (i.e., bias towards the null), or would result in a spurious effect when results suggest no effect.

1. **Overall confidence conclusions**

The results of the quality of evidence assessment will be summarized in a separate Evidence Profile Table for each critical outcome, by line of evidence and specific exposure sources (Table 1).

Our overall conclusions will be primarily based on the line of evidence with the highest confidence when considered across the multiple exposure-neoplasm pairs examined.

- 1. *Confidence in evidence across multiple exposure types*

In developing the conclusions, we will assess the coherence of neoplasm-specific findings across the different types and sources of exposure to RF-EMF considered in the systematic review: mobile phones, cordless phones, broadcast transmitters, base stations, and several types of occupational exposures.

As reasons for variation in results, we will focus on differences in exposure levels by source (Birks et al. 2021; Cabré-Riera et al. 2020; Hareuveny et al. 2015; Lauer et al. 2013; Liorni et al. 2020; Migault et al. 2019; Roser et al. 2015; Roser et al. 2017; van Wel et al. 2021; Vila et al. 2016). Other characteristics of the exposure (e.g., frequency band, modulation, or body localisation) could also be relevant in principle, although current scientific evidence in support of such specific effects is weak (Sienkiewicz et al. 2017).

If the overall pattern of findings supports an exposure-outcome association, and provided that there are no relevant differences in the quality of evidence between exposure-specific datasets or other dissuading concerns, we will develop a combined confidence rating for any exposure to RF-EMF.

Confidence in the combined body of evidence for a given outcome would be enhanced by a pattern of effect magnitude in line with a ranking of the exposure sources based on their relative contribution to the absorption of RF energy at the target organ/tissue, or lessened by exposure-disease associations of similar strength across different levels of exposure.

- 1. *Confidence in evidence across multiple endpoints*

We will not combine findings from studies investigating risk of different neoplasms. However, we will examine results for related endpoints. For example, we will consider the evidence concerning the effect of near-field exposure to RF-EMF from mobile phones, cordless phones and other hand-held transceivers on risk of different tumours in the head region, and discuss it in the light of the quality of evidence of each exposure-outcome dataset, dosimetric considerations (Cabré-Riera et al. 2020; Cardis et al. 2008; Dimbylow and Mann 1999; Lauer et al. 2013; Liorni et al. 2020), the presumed tumour-specific induction-latency period (Lee and Wernicke 2016; McNeill 2016; Schneider et al. 2008; Yamanaka et al. 2017; 2018), and other relevant complementary evidence, such as findings from simulation studies that have assessed the external plausibility of the measures of effect for brain cancer, glioma or subtypes of central nervous system tumours by comparing predicted and observed incidence rates (Chapman et al. 2016; de Vocht 2016; 2019; Deltour et al. 2012; Karipidis et al. 2018; Little et al. 2012; Sato et al. 2019; Villeneuve et al. 2021).

**Table 1. Template of the Evidence Profile table (one critical outcome shown as example)**

| **Initial Confidence for Each Body of Evidence** (# of Studies by design)  Moderate (+++) | **Factors decreasing confidence** (" -" if no concern; "↓" if serious concern to downgrade confidence) | | | | **Factors increasing confidence** (" -" if not present; "↑" if sufficient to upgrade confidence) | | | | **Final Confidence Rating**  High (++++)  Moderate (+++)  Low (++)  Very Low (+) |
| --- | --- | --- | --- | --- | --- | --- | --- | --- | --- |
|  | Risk of Bias | Inconsistency | Indirectness | Imprecision | Publication Bias | Large Magnitude | Dose-Response | Confounding |  |
| **Outcome:** (e.g., Glioma/Brain Cancer) | | | | | | | | | |
| Near-field, head localized, exposure from wireless phones (SR-A): **Mobile phones** | | | | | | | | | |
|  | a | b | c | d | e | f | g | h |  |
| Near-field, head localized, exposure from wireless phones (SR-A): **Cordless phones** | | | | | | | | | |
|  | a | b | c | d | e | f | g | h |  |
| Far-field, whole body, exposure from environmental sources (SR-B): **Broadcast transmitters** | | | | | | | | | |
|  | a | b | c | d | e | f | g | h |  |
| Far-field, whole body, exposure from environmental sources (SR-B): **Base stations** | | | | | | | | | |
|  | a | b | c | d | e | f | g | h |  |
| Near-field, head localized, occupational exposure (SR-C): **Hand-held transceivers** | | | | | | | | | |
|  | a | b | c | d | e | f | g | h |  |
| Near/Far-field, whole body/localized, occupational exposure (SR-C): **RF-emitting equipment** | | | | | | | | | |
|  | a | b | c | d | e | f | g | h |  |
| **Exposure to RF-EMF** | | | | | | | | | |
|  | j | | | | | | | |  |
| **Note** - The following information will be outlined and discussed in the narrative summary of the evidence assessment: ^a^ Trend, key questions and issues; ^b^ Results in terms of consistency, and reasons for apparent inconsistency (if possible); ^c^ Indicators or populations with less relevance; ^d^ Confidence intervals, and other sensitivity issues; ^e^ Possible indications of publication bias; ^f^ Magnitude of response; ^g^ Evidence for or against dose response; ^h^ Evidence of confounding toward null; ^I^ Coherence between effect magnitude and source ranking by exposure level. | | | | | | | | | |

**References**

Arroyave WD, Mehta SS, Guha N, et al. Challenges and recommendations on the conduct of systematic reviews of observational epidemiologic studies in environmental and occupational health. J Expo Sci Environ Epidemiol 2021;31:21-30.

Birks LE, van Wel L, Liorni I, et al. Radiofrequency electromagnetic fields from mobile communication: Description of modeled dose in brain regions and the body in European children and adolescents. Environ Res 2021;193:110505.

Cabré-Riera A, Marroun HE, Muetzel R, et al. Estimated whole-brain and lobe-specific radiofrequency electromagnetic fields doses and brain volumes in preadolescents. Environ Int 2020;142:105808.

Cardis E, Deltour I, Mann S, et al. Distribution of RF energy emitted by mobile phones in anatomical structures of the brain. Phys Med Biol 2008;53:2771-2783.

Chapman S, Azizi L, Luo Q, et al. Has the incidence of brain cancer risen in Australia since the introduction of mobile phones 29 years ago? Cancer Epidemiol 2016;42:199-205.

Clark C, Paunovic K. WHO Environmental Noise Guidelines for the European Region: A Systematic Review on Environmental Noise and Cognition. Int J Environ Res Public Health 2018;15

de Vocht F. Inferring the 1985-2014 impact of mobile phone use on selected brain cancer subtypes using Bayesian structural time series and synthetic controls. Environ Int 2016;97:100-107.

de Vocht F. Analyses of temporal and spatial patterns of glioblastoma multiforme and other brain cancer subtypes in relation to mobile phones using synthetic counterfactuals. Environ Res 2019;168:329-335.

Dekkers OM, Vandenbroucke JP, Cevallos M, et al. COSMOS-E: Guidance on conducting systematic reviews and meta-analyses of observational studies of etiology. PLoS Med 2019;16:e1002742.

Deltour I, Auvinen A, Feychting M, et al. Mobile phone use and incidence of glioma in the Nordic countries 1979-2008: consistency check. Epidemiology 2012;23:301-307.

Dimbylow PJ, Mann SM. Characterisation of energy deposition in the head from cellular phones. Radiat Prot Dosimetry 1999;83:139-141.

Eden J, Levit L, Berg A, et al. eds. Finding what works in healthcare. Standards for systematic reviews. Washington, DC: National Academy of Sciences; 2011.

Guyatt GH, Oxman AD, Sultan S, et al. GRADE guidelines: 9. Rating up the quality of evidence. J Clin Epidemiol 2011;64:1311-1316.

Hareuveny R, Kavet R, Shachar A, et al. Occupational exposures to radiofrequency fields: results of an Israeli national survey. J Radiol Prot 2015;35:429-445.

Héroux M-È, Verbeek J. Methodology for systematic evidence reviews for WHO environmental noise guidelines for the European Region ed^eds. Copenhagen: WHO Regional Office for Europe; 2018.

Higgins JPT, Lasserson T, Chandler J, et al. eds. Methodological expectations of Cochrane intervention reviews (MECIR). London: Cochrane; 2020.

Johansen C, Schüz J, Andreasen AS, et al. Study designs may influence results: the problems with questionnaire-based case-control studies on the epidemiology of glioma. Br J Cancer 2017;116:841-848.

Johnson PI, Koustas E, Vesterinen HM, et al. Application of the Navigation Guide systematic review methodology to the evidence for developmental and reproductive toxicity of triclosan. Environ Int 2016;92-93:716-728.

Johnson PI, Sutton P, Atchley DS, et al. The Navigation Guide - evidence-based medicine meets environmental health: systematic review of human evidence for PFOA effects on fetal growth. Environ Health Perspect 2014;122:1028-1039.

Karipidis K, Elwood M, Benke G, et al. Mobile phone use and incidence of brain tumour histological types, grading or anatomical location: a population-based ecological study. BMJ Open 2018;8:e024489.

Katikireddi SV, Egan M, Petticrew M. How do systematic reviews incorporate risk of bias assessments into the synthesis of evidence? A methodological study. J Epidemiol Community Health 2015;69:189-195.

Kempen EV, Casas M, Pershagen G, et al. WHO Environmental Noise Guidelines for the European Region: A Systematic Review on Environmental Noise and Cardiovascular and Metabolic Effects: A Summary. Int J Environ Res Public Health 2018;15

Lam J, Lanphear BP, Bellinger D, et al. Developmental PBDE Exposure and IQ/ADHD in Childhood: A Systematic Review and Meta-analysis. Environmental Health Perspectives 2017;125:086001.

Lauer O, Frei P, Gosselin MC, et al. Combining near- and far-field exposure for an organ-specific and whole-body RF-EMF proxy for epidemiological research: a reference case. Bioelectromagnetics 2013;34:366-374.

Lawlor DA, Tilling K, Davey Smith G. Triangulation in aetiological epidemiology. Int J Epidemiol 2016;45:1866-1886.

Lee JW, Wernicke AG. Risk and survival outcomes of radiation-induced CNS tumors. J Neurooncol 2016;129:15-22.

Liorni I, Capstick M, van Wel L, et al. Evaluation of specific absorption rate in the far-field, near-to-far field and near-field regions for integrative radiofrequency exposure assessment. Radiat Prot Dosimetry 2020;190:459-472.

Little MP, Rajaraman P, Curtis RE, et al. Mobile phone use and glioma risk: Comparison of epidemiological study results with incidence trends in the United States. BMJ 2012;344:e1147.

McNeill KA. Epidemiology of Brain Tumors. Neurol Clin 2016;34:981-998.

Migault L, Bowman JD, Kromhout H, et al. Development of a job-exposure matrix for assessment of occupational exposure to high-frequency electromagnetic fields (3 kHz-300 GHz). Ann Work Expo Health 2019;63:1013-1028.

Morgan RL, Beverly B, Ghersi D, et al. GRADE guidelines for environmental and occupational health: A new series of articles in Environment International. Environ Int 2019;128:11-12.

Morgan RL, Thayer KA, Bero L, et al. GRADE: Assessing the quality of evidence in environmental and occupational health. Environ Int 2016;92-93:611-616.

Nieuwenhuijsen MJ, Ristovska G, Dadvand P. WHO Environmental Noise Guidelines for the European Region: A Systematic Review on Environmental Noise and Adverse Birth Outcomes. Int J Environ Res Public Health 2017;14

NTP-OHAT. Handbook for Conducting a Literature-Based Health Assessment Using OHAT Approach for Systematic Review and Evidence Integration (March 4, 2019). National Toxicology Program - Office of Health Assessment and Translation; 2019.

NTP-ORoC. Handbook for Preparing Report on Carcinogens Monographs. Durham: National Toxicology Program - Office of the Report on Carcinogens; 2015.

NTP. NTP Monograph on immunotoxicity associated with exposure to perfluorooctanoic acid or perfluorooctane sulfonate. Research Triangle Park: Office of Health Assessment and Translation; 2016.

NTP. NTP Monograph on the systematic review of long-term neurological effects following acute exposure to Sarin. Research Triangle Park: Office of Health Assessment and Translation; 2019a.

NTP. NTP Monograph on the systematic review of occupational exposure to cancer chemotherapy agents and adverse health outcomes. Research Triangle Park: Office of Health Assessment and Translation; 2019b.

NTP. NTP Monograph on the systematic review of traffic-related air pollution and hypertensive disorders of pregnancy. Research Triangle Park: Office of Health Assessment and Translation; 2019c.

Rooney AA, Boyles AL, Wolfe MS, et al. Systematic review and evidence integration for literature-based environmental health science assessments. Environ Health Perspect 2014;122:711-718.

Rooney AA, Cooper GS, Jahnke GD, et al. How credible are the study results? Evaluating and applying internal validity tools to literature-based assessments of environmental health hazards. Environ Int 2016;92-93:617-629.

Roser K, Schoeni A, Burgi A, et al. Development of an RF-EMF exposure surrogate for epidemiologic research. Int J Environ Res Public Health 2015;12:5634-5656.

Roser K, Schoeni A, Struchen B, et al. Personal radiofrequency electromagnetic field exposure measurements in Swiss adolescents. Environ Int 2017;99:303-314.

Samet JM, Chiu WA, Cogliano V, et al. The IARC Monographs: Updated Procedures for Modern and Transparent Evidence Synthesis in Cancer Hazard Identification. J Natl Cancer Inst 2020;112:30-37.

Sato Y, Kojimahara N, Yamaguchi N. Simulation of the incidence of malignant brain tumors in birth cohorts that started using mobile phones when they first became popular in Japan. Bioelectromagnetics 2019;40:143-149.

Savitz DA, Wellenius GA, Trikalinos TA. The problem with mechanistic risk of bias assessments in evidence synthesis of observational studies and a practical alternative: Assessing the impact of specific sources of potential bias. Am J Epidemiol 2019;188:1581-1585.

Schneider AB, Ron E, Lubin J, et al. Acoustic neuromas following childhood radiation treatment for benign conditions of the head and neck. Neuro Oncol 2008;10:73-78.

Schünemann HJ, Cuello C, Akl EA, et al. GRADE guidelines: 18. How ROBINS-I and other tools to assess risk of bias in nonrandomized studies should be used to rate the certainty of a body of evidence. J Clin Epidemiol 2019;111:105-114.

Schünemann HJ, Higgins JPT, Vist GE, et al. Chapter 14: Completing ‘Summary of findings’ tables and grading the certainty of the evidence. in: Higgins J.P.T., Thomas J., Chandler J., Cumpston M., Li T., Page M.J., Welch V.A., eds. Cochrane Handbook for Systematic Reviews of Interventions version 6-2 (updated February 2021): Cochrane; 2021.

Sienkiewicz Z, Calderon C, Broom KA, et al. Are exposures to multiple frequencies the key to future radiofrequency research? Front Public Health 2017;5:328.

Steenland K, Schubauer-Berigan MK, Vermeulen R, et al. Risk of bias assessments and evidence syntheses for observational epidemiologic studies of environmental and occupational exposures: Strengths and limitations. Environ Health Perspect 2020;128:95002.

Thayer KA, Schünemann HJ. Using GRADE to respond to health questions with different levels of urgency. Environ Int 2016;92-93:585-589.

van Wel L, Liorni I, Huss A, et al. Radio-frequency electromagnetic field exposure and contribution of sources in the general population: an organ-specific integrative exposure assessment. J Expo Sci Environ Epidemiol 2021;epub 2021/03/02

Verbeek J, Oftedal G, Feychting M, et al. Prioritizing health outcomes when assessing the effects of exposure to radiofrequency electromagnetic fields: A survey among experts. Environ Int 2021;146:106300.

Vesterinen HM, Morello-Frosch R, Sen S, et al. Cumulative effects of prenatal-exposure to exogenous chemicals and psychosocial stress on fetal growth: Systematic-review of the human and animal evidence. Plos One 2017;12:e0176331.

Vila J, Bowman JD, Richardson L, et al. A source-based measurement database for occupational exposure assessment of electromagnetic fields in the INTEROCC study: A literature review approach. Ann Occup Hyg 2016;60:184-204.

Villeneuve PJ, Momoli F, Parent ME, et al. Cell phone use and the risk of glioma: are case-control study findings consistent with Canadian time trends in cancer incidence? Environ Res 2021:111283.

Whaley P, Aiassa E, Beausoleil C, et al. Recommendations for the conduct of systematic reviews in toxicology and environmental health research (COSTER). Environ Int 2020;143

WHO. Handbook for Guideline Development. Second edition. Geneva: World Health Organization; 2014.

WHO. Environmental Noise Guidelines for the European Region ed^eds. Copenhagen: WHO Regional Office for Europe; 2018.

Woodruff TJ, Sutton P. The Navigation Guide systematic review methodology: a rigorous and transparent method for translating environmental health science into better health outcomes. Environ Health Perspect 2014;122:1007-1014.

Yamanaka R, Hayano A, Kanayama T. Radiation-induced meningiomas: An exhaustive review of the literature. World Neurosurg 2017;97:635-644 e638.

Yamanaka R, Hayano A, Kanayama T. Radiation-induced gliomas: A comprehensive review and meta-analysis. Neurosurg Rev 2018;41:719-731.
